# Metathesis between E−C(sp^*n*^) and H−C(sp^3^) σ‐Bonds (E=Si, Ge; *n*=2, 3) on an Osmium‐Polyhydride

**DOI:** 10.1002/anie.202204081

**Published:** 2022-05-31

**Authors:** Miguel A. Esteruelas, Ana M. López, Enrique Oñate, Esther Raga

**Affiliations:** ^1^ Departamento de Química Inorgánica Instituto de Síntesis Química y Catálisis Homogénea (ISQCH) Centro de Innovación en Química Avanzada (ORFEO-CINQA) Universidad de Zaragoza-CSIC 50009 Zaragoza Spain

**Keywords:** Germanes, Osmium, Polyhydrides, Silanes, σ-Bond Metathesis

## Abstract

The silylation of a phosphine of OsH_6_(P^i^Pr_3_)_2_ is performed via net‐metathesis between Si−C(sp^*n*^) and H−C(sp^3^) σ‐bonds (*n*=2, 3). Complex OsH_6_(P^i^Pr_3_)_2_ activates the Si−H bond of Et_3_SiH and Ph_3_SiH to give OsH_5_(SiR_3_)(P^i^Pr_3_)_2_, which yield OsH_4_{κ^1^‐P,η^2^‐SiH‐[^i^Pr_2_PCH(Me)CH_2_SiR_2_H]}(P^i^Pr_3_) and R−H (R=Et, Ph), by displacement of a silyl substituent with a methyl group of a phosphine. Such displacement is a first‐order process, with activation entropy consistent with a rate determining step occurring via a highly ordered transition state. It displays selectivity, releasing the hydrocarbon resulting from the rupture of the weakest Si‐substituent bond, when the silyl ligand bears different substituents. Accordingly, reactions of OsH_6_(P^i^Pr_3_)_2_ with dimethylphenylsilane, and 1,1,1,3,5,5,5‐heptamethyltrisiloxane afford OsH_5_(SiR_2_R′)(P^i^Pr_3_)_2_, which evolve into OsH_4_{κ^1^‐P,η^2^‐GeH‐[^i^Pr_2_PCH(Me)CH_2_SiR_2_H]}(P^i^Pr_3_) (R=Me, OSiMe_3_) and R′−H (R′=Ph, Me). Exchange reaction is extended to Et_3_GeH. The latter reacts with OsH_6_(P^i^Pr_3_)_2_ to give OsH_5_(GeEt_3_)(P^i^Pr_3_)_2_, which loses ethane to form OsH_4_{κ^1^‐P,η^2^‐GeH‐[^i^Pr_2_PCH(Me)CH_2_GeEt_2_H]}(P^i^Pr_3_).

## Graphical abstract

A series of pentahydride‐silyl/germyl‐osmium(VI) derivatives was prepared. These d^2^‐species of a late transition metal undergo a novel metathesis reaction between non‐activated E−C(sp^*n*^) and H−C(sp^3^) σ‐bonds (E=Si, Ge; *n*=2, 3) affording new osmium(IV) tetrahydride complexes bearing phosphino‐silane and phosphino‐germane chelate ligands.

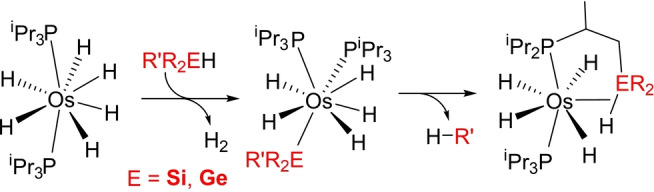

## Introduction

**Scheme 1:**
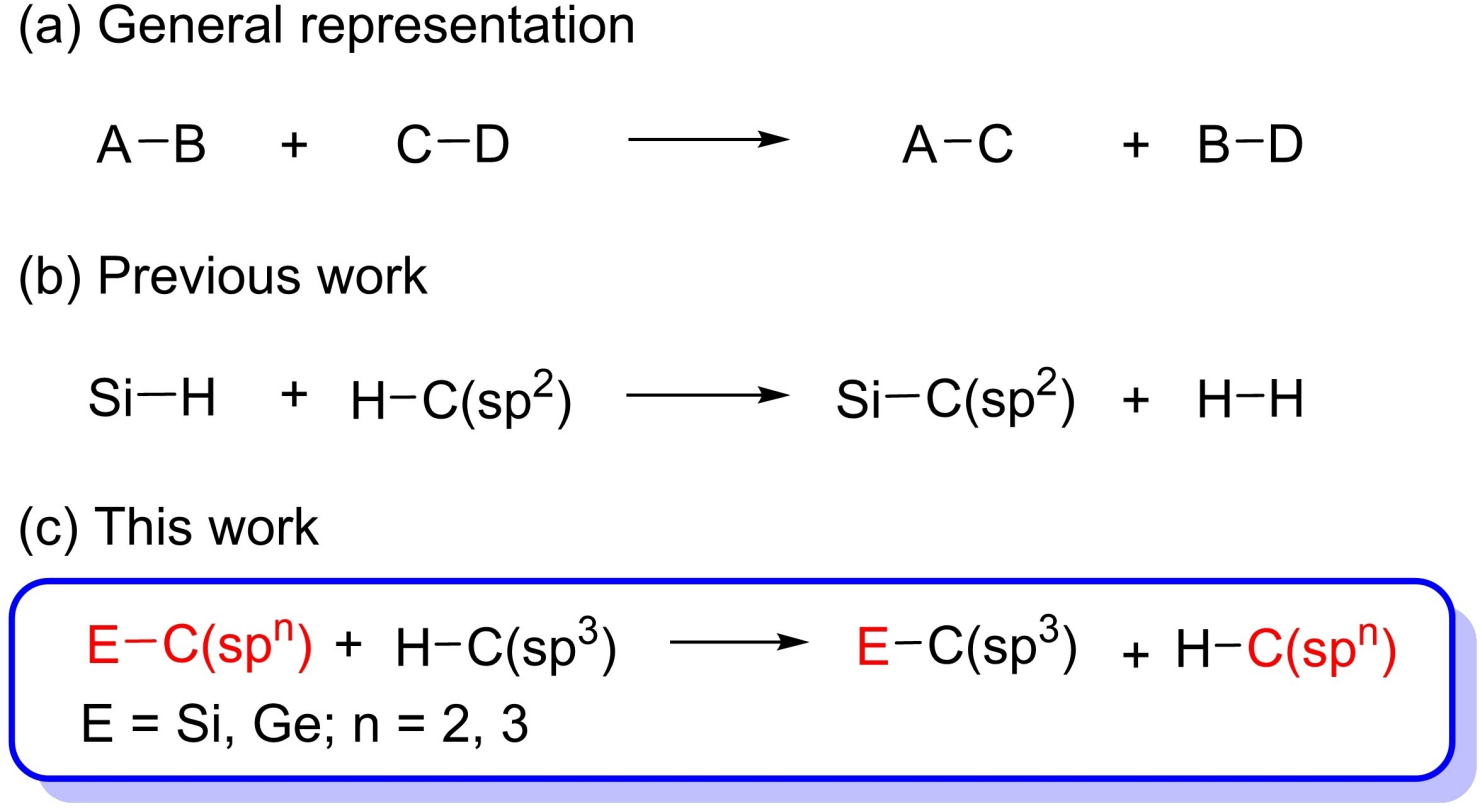
Metathesis reactions between σ‐bonds mediated by transition metal complexes.

Metathesis reactions between single nonpolar bonds mediated by transition metal complexes are challenging transformations from a conceptual point of view, which involve elemental steps of σ‐bond activation in both substrates and subsequent cross‐coupling of the resulting fragments. They can be described in a general manner according to Equation (a) (Scheme 1). The C−H silylation with hydrosilanes is a particular type of this class of reactions [Eq. (b) in Scheme 1],^[1]^ which displays high chemoselectivity.^[2]^ It is mainly centered on the functionalization of C(sp^2^)−H bonds,^[3]^ while the functionalization of C(sp^3^)−H bonds is limited to some particular cases and still remains a great challenge.^[4]^ As far as we know, C(sp^3^)−H silylation by σ‐activation/cross‐coupling metathesis involving Si−C cleavage instead of Si−H rupture has not been described [Eq. (c) in Scheme 1]. This is probably because the Si−H bond activation is kinetically and thermodynamically favored with respect to the Si−C bond rupture.^[5]^

The C(sp^3^)−H silylation by such metathesis involving Si−C cleavage needs sequencing the cleavage of the Si−C and C(sp^3^)−H σ‐bonds in the metal coordination sphere. The Si−C bond activation by transition metal complexes is difficult; exceptionally uncommon is the rupture of inactivated Si−C(sp^3^) bonds due to their high dissociation energy and low polarity. It requires strongly nucleophilic metal centers in low oxidation state. There are a few examples of oxidative additions promoted by the formation of pincer or chelating ligands^[6]^ and cases involving strained silicon‐containing rings.^[7]^ The simple intermolecular Si−C(sp^3^) oxidative addition of alkyl, alkoxy, and siloxysilanes has been only very recently achieved by Chapp and Schley, with a cationic pincer‐supported iridium system.^[8]^ Previously, Puddephatt had observed that the protonolysis of a (trimethylsilyl)methyl‐platinum bond led to a methyl‐platinum‐(trimethylsilyl) derivative.^[9]^ A successful access has been also the alkyl α‐elimination on a silyl ligand coordinated to unsaturated tungsten,^[10]^ ruthenium,^[11]^ or iridium^[12]^ centers, to afford silylene compounds. Classical metal‐promoted C(sp^3^)−H bond activation takes place via M(η^2^‐CH) intermediates, which evolve into alkyl derivatives by oxidative addition, heterolytic cleavage,^[13]^ or σ‐bond metathesis.^[14]^ A different approach involves C−H addition across a metal–ligand multiple bond. Such activation has been mainly performed employing imido complexes of early metals and electrophilic Schrock‐type carbene compounds.^[15]^ However, the 1,2‐additions of nonpolar C(sp^3^)−H bonds to metal‐silylenes are very rare.^[16]^ Sekiguchi has isolated a hafnium–silylene, which undergoes cyclometallation via 1,2‐CH addition of a silylene substituent across the Hf−Si double bond,^[17]^ whereas Tilley has observed a similar intramolecular C−H activation and addition of a flanking mesityl methyl group across a Fe−Si double bond.^[18]^

Polyhydrides of platinum group metals show a highly diverse reactivity, where the σ‐bond activation reactions are preponderate.^[19]^ The d^2^‐hexahydride complex OsH_6_(P^i^Pr_3_)_2_ (**1**) occupies a privileged position within this class of compounds. In addition to activation of σ‐bonds of a wide range of organic molecules,^[20]^ including β‐lactams^[21]^ and nucleosides,^[22]^ it shows interesting applications in catalysis^[23]^ and is the synthetic precursor to novel Os^II^ and Os^IV^ phosphorescent emitters.^[24]^ Although its chemistry is dominated by the reactions of C−H bond activation,[^20c^, ^20d^, ^20f^, ^25^] the Si−H bond rupture mediated by this polyhydride is unexplored. Such gap in the chemistry of one of most relevant polyhydrides of the platinum group metals prompted us to study its behavior towards R_3_SiH and R′R_2_SiH silanes. During the study, we discovered that one of the alkyl phosphines of **1** is able to undergo a surprising σ‐activation/cross‐coupling metathesis, which fits to the reaction summarized by Equation (c) of Scheme 1. This paper reports about this discovery.

## Results and Discussion

**Scheme 2:**
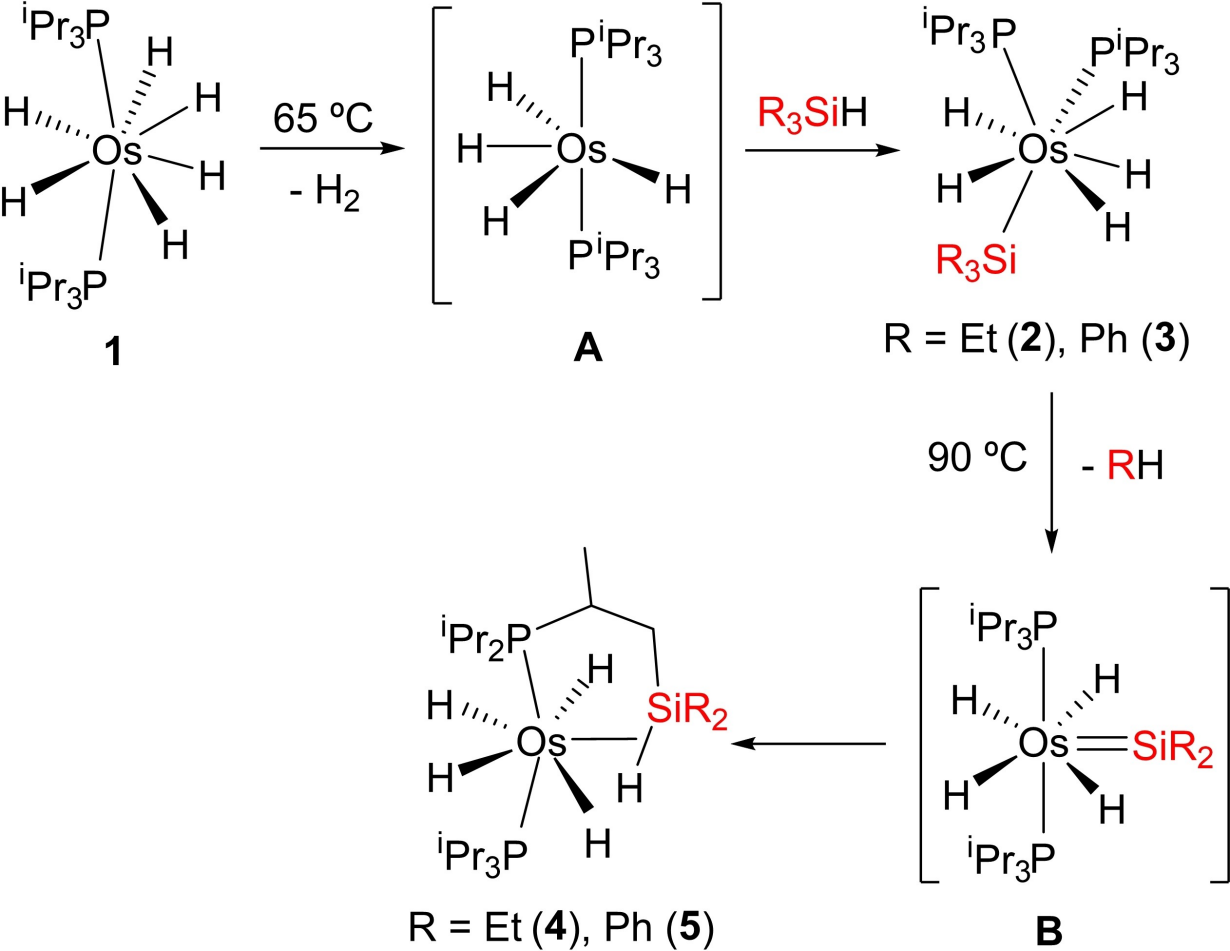
σ‐Activation/cross‐coupling metathesis according to Equation (c) applied to R_3_SiH: Preparation of complexes **2–4**.

Complex **1** activates the Si−H bond of R_3_SiH silanes. Treatment of solutions of the hexahydride complex in octane, with 1.0 equiv of triethylsilane and triphenylsilane, at 65 °C, for 4 h leads to the pentahydride‐osmium(VI)‐silyl derivatives OsH_5_(SiR_3_)(P^i^Pr_3_)_2_ (R=Et (**2**), Ph (**3**)), which are unstable in solution and evolve into OsH_4_{κ^1^‐P,η^2^‐SiH‐[^i^Pr_2_PCH(Me)CH_2_SiR_2_H]}(P^i^Pr_3_) (R=Et (**4**), Ph (**5**)). The transformation results from the displacement of one of the R substituents of the silyl ligand by a methyl group of an isopropyl substituent of a phosphine. At 90 °C, in toluene, these σ‐bond metathesis reactions [Eq. (c)] are quantitative after 6 h (Scheme 2).

**Figure 1:**
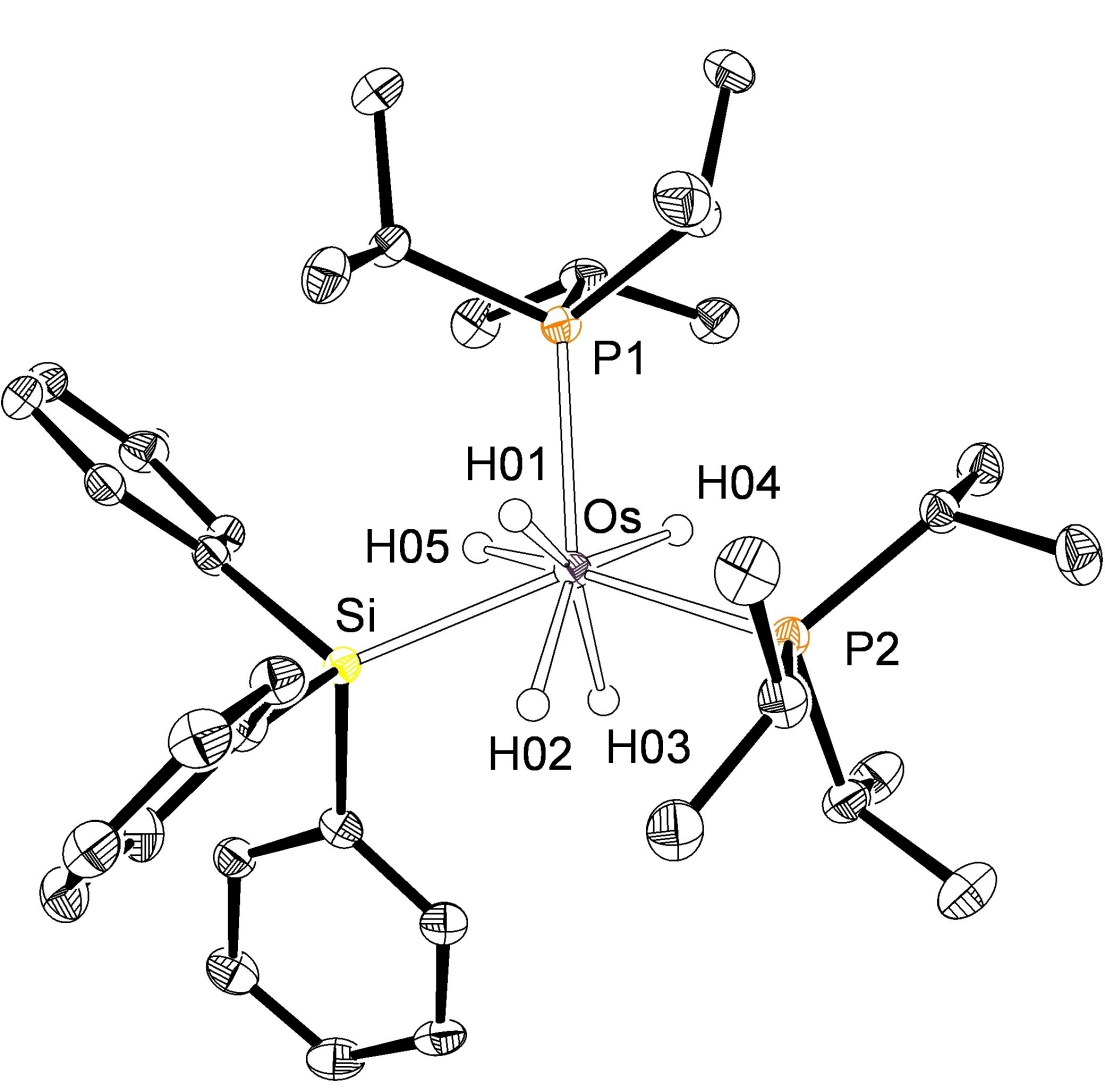
X‐ray structure of complex **3** with 50 % probability ellipsoids. Hydrogen atoms (except hydrides) are omitted for clarity. Selected bond lengths [Å] and angles [deg] for the X‐ray and DFT optimized (in square brackets) structures: Os−H(01) 1.581(10) [1.662], Os−H(02) 1.582(10) [1.635], Os−H(03) 1.593(10) [1.629], Os−H(04) 1.593(10) [1.657], Os−H(05) 1.582(10) [1.638], Os−P(1) 2.3996(8) [2.448], Os−P(2) 2.3965(8) [2.442], Os−Si 2.4312(9) [2.458], Si−H(02) [2.174], Si−H(05) [2.123]; P(1)‐Os‐P(2) 110.92(3) [109.84], Si‐Os‐P(1) 109.70(3) [106.95], Si‐Os‐P(2) 133.72(3) [134.04].

The generation rates of **2** and **4** are comparable. As a consequence, the transformation of **2** into **4** starts before the formation of **2** is complete. In contrast, the generation of **3** is significantly faster than the formation of **5** and therefore intermediate **3** can be isolated as analytically pure crystals suitable for X‐ray diffraction analysis.^[26]^ Figure 1 shows a view of its structure. The polyhedron around the metal center can be described as a dodecahedron, in agreement with the structures of OsH_6_P_2_‐species^[27]^ and related eight‐coordinate osmium‐polyhydrides.^[28]^ The dodecahedron is defined by two intersecting BAAB orthogonal (88(6)°) trapezoidal planes. One of them contains the atoms P(1), H(01), H(02), and H(03), whereas the other is formed by the atoms P(2), H(04), H(05), and Si. The angle between the heavy atoms lying in the same plane, P(2)‐Os‐Si, of 133.72(3)° is significantly more open than those between heavy atoms situated at different planes, P(1)‐Os‐P(2) and Si‐Os‐P(1), of 110.92(3) and 109.70(3)°, which strongly deviate of the ideal value of 90°. The classical nature of the hydride ligands was confirmed by the DFT optimized structure, which yields separations between these ligands longer than 1.80 Å. It also reveals relatively short separations between the silicon atom and the hydrides H(02) and H(05) of 2.174 and 2.123 Å, respectively, which are consistent with the existence of the denoted *“secondary interactions between silicon and hydrogen atoms (SISHA)*”.[^16a^, ^29^] However, Atoms in Molecules (AIM) calculations do not show any bond path running between the involved atoms. Thus, the proximity between them seems to be related to their sizes and the geometry of the complex, but not to the existence of any bonding interaction.

The structure of **2** as well as that of **3** are not rigid in hydrocarbon solutions. The hydride ligands of these compounds are involved in thermally activated position exchange processes, even at 153 K in methylcyclohexane‐*d*_14_. In accordance with this, their ^1^H‐NMR spectra at room temperature show in the high field region a triplet at −10.70 ppm (^2^ *J*_H,P_=6.4 Hz) for **2** and −9.67 ppm (^2^ *J*_H,P_=8.4 Hz) for **3**. Lowering temperature of the sample leads to a broadening of the resonance. However, decoalescence is not reached. The phosphine ligands also average their positions in solution. Thus, the ^31^P{^1^H} NMR spectra contain a singlet for the inequivalent phosphines, at about 39 ppm, in the temperature range 293–153 K. A triplet (^2^ *J*_Si,P_≈4 Hz) close to −2 ppm in the ^29^Si{^1^H} NMR spectra is also a characteristic feature of these polyhydrides.

**Figure 2:**
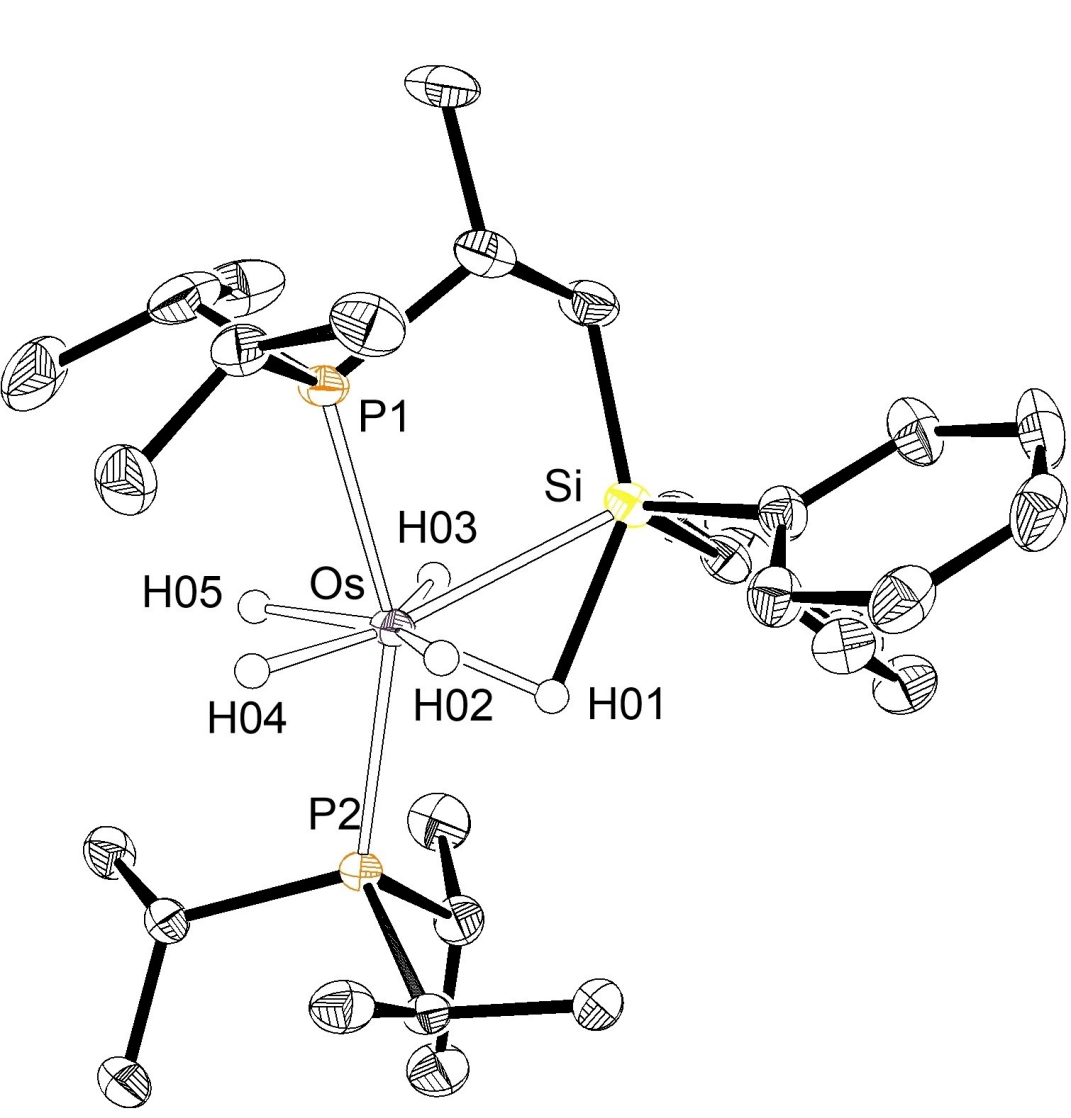
X‐ray structure of complex **5** with 50 % probability ellipsoids. Hydrogen atoms (except hydrides) are omitted for clarity. Selected bond lengths [Å] and angles [deg] for the X‐ray and DFT optimized (in square brackets) structures: Os−H(01) 1.584(9) [1.666], Os−H(02) 1.577(9) [1.667], Os−H(03) 1.580(9) [1.674], Os−H(04) 1.592(9) [1.648], Os−H(05) 1.590(9) [1.641], Os−P(1) 2.3512(6) [2.376], Os−P(2) 2.3449(6) [2.377], Os−Si 2.4645(6) [2.512], Si−H(01) 1.91(2) [1.847]; P(1)‐Os‐P(2) 157.56(2) [164.01], Si‐Os‐P(1) 80.98(2) [80.29], Si‐Os‐P(2) 121.302(19) [115.56].

The presence of a silylated phosphine in **4** and **5** was confirmed by means of the X‐ray diffraction structure of **5**.^[26]^ Figure 2 gives a view of the molecule. The polyhedron around the osmium atom can be described as a pentagonal bipyramid with the phosphorus atoms of the phosphines at the apical positions (P(1)‐Os‐P(2) 157.56(2)°), whereas the hydride ligands and the Si−H bond define the base of the polyhedron. The Si−H(01) distance of 1.91(2) Å lies within the range reported for the compounds, bearing a Si−H bonding interaction, which are called “*asymmetric oxidative addition products (ASOAP)*”.[^16a^, ^29b^] The DFT optimized structure corroborates a short Si−H(01) distance (1.847 Å) and points out a classical nature of the hydride ligands, which are separated by more than 1.85 Å. To gain information about the nature of the osmium‐hydrogen‐silicon interaction, we also performed DFT calculations using the AIM method. The complex exhibits significant Os−Si, Si−H, and H−Os interactions as revealed by the occurrence of bond critical points located between the respective atoms, which are associated with bond paths running between them. This triangular topology, which is characteristic of elongated σ‐bonds acting as 2e‐donor ligands,^[30]^ is complemented by a ring critical point (Figure S4).

The Si−H bond of **4** and **5** does not prevent a position exchange of low activation energy between such hydrogen atom and the hydride ligands, which are as well involved in the typical movements of this class of polyhydrides in solution. Thus, their ^1^H‐NMR spectra, in hydrocarbons, at room temperature show only one resonance, for the five hydrogen atoms coordinated to the metal center, which appears at about −10.6 ppm as a doublet of doublets with H−P coupling constants of around 14 and 10 Hz. Although a broadening of the signal is observed upon lowering the temperature of the sample, decoalescence does not take place even at 153 K, in methylcyclohexane‐*d*_14_. In contrast to the hydrides, the heavy atoms keep their positions in the structure. In the same temperature range, the ^31^P{^1^H} NMR spectra contain an AB spin system at 64.6 ppm (Δν=2541 Hz, *J*_A–B_=152 Hz) for **4** and 63.8 ppm (Δν=2693 Hz, *J*_A–B_=148 Hz) for **5**, whereas the ^29^Si{^1^H} NMR spectra show a doublet of doublets at 26.1 ppm (^2^ *J*_Si,P_=18.2 and 13.9 Hz) for **4** and 18.7 ppm (^2^ *J*_Si,P_=19.6 and 15.5 Hz) for **5**.

The formation of **2** and **3** takes place via the unsaturated species OsH_4_(P^i^Pr_3_)_2_ (**A** in Scheme 2), which undergoes the Si−H oxidative addition of the silanes. Tetrahydride **A** is generated by reductive elimination of molecular hydrogen from **1** and has been trapped with pyridines,^[23b]^ 2,6‐dimethylbenzonitrile,^[31]^ and boranes.^[30a]^ The coordination of the nitrogenous Lewis bases yields tetrahydride compounds of the class OsH_4_(P^i^Pr_3_)_2_L, whereas the boranes afford the trihydride‐dihydroborate complexes OsH_3_{κ^2^‐*H*,*H*‐(H_2_BR_2_)}(P^i^Pr_3_)_2_. In contrast to **A**, the related chloride OsH_3_Cl(PPh_3_)_3_ reacts with diphenylsilane to give OsH_3_(SiClPh_2_)(PPh_3_)_3_ and OsH_4_(SiClPh_2_)(SiHPh_2_)(PPh_3_)_2_ and with phenylsilane to afford OsH_4_(SiClHPh)(SiH_2_Ph)(PPh_3_)_2_.^[32]^

**Figure 3:**
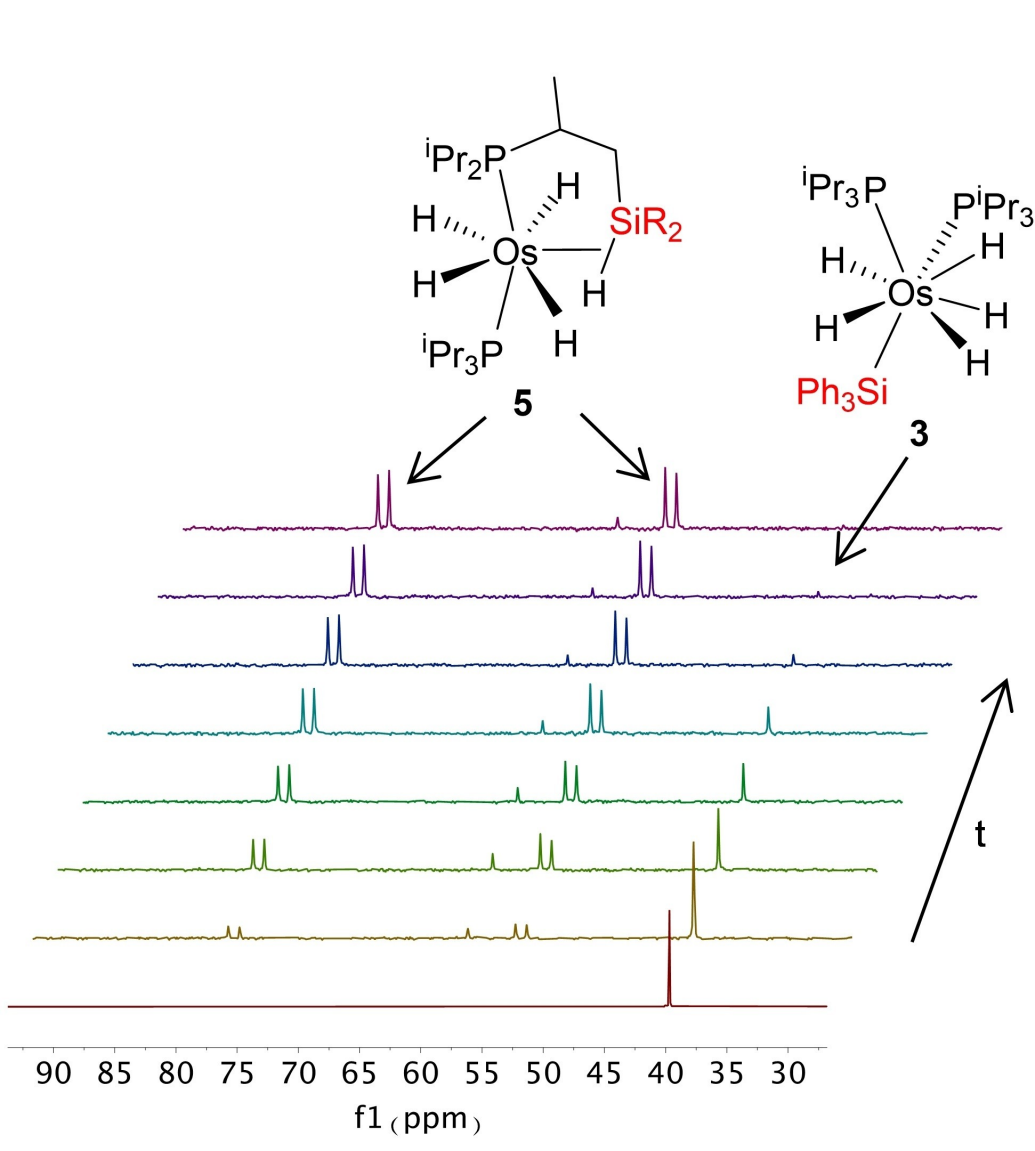
^31^P{^1^H} NMR spectra (161.98 MHz) showing the transformation of **3** into **5** in toluene at 378 K.

**Figure 4:**
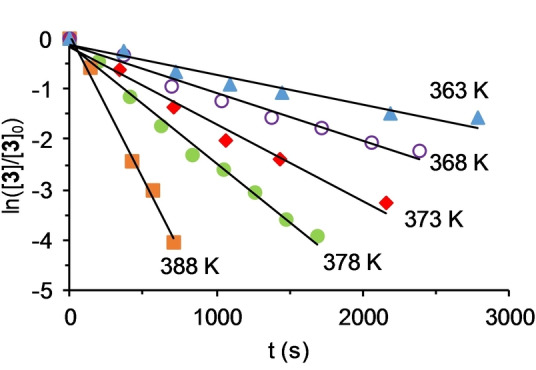
Plot of Equation (1) at different temperatures.

**Figure 5:**
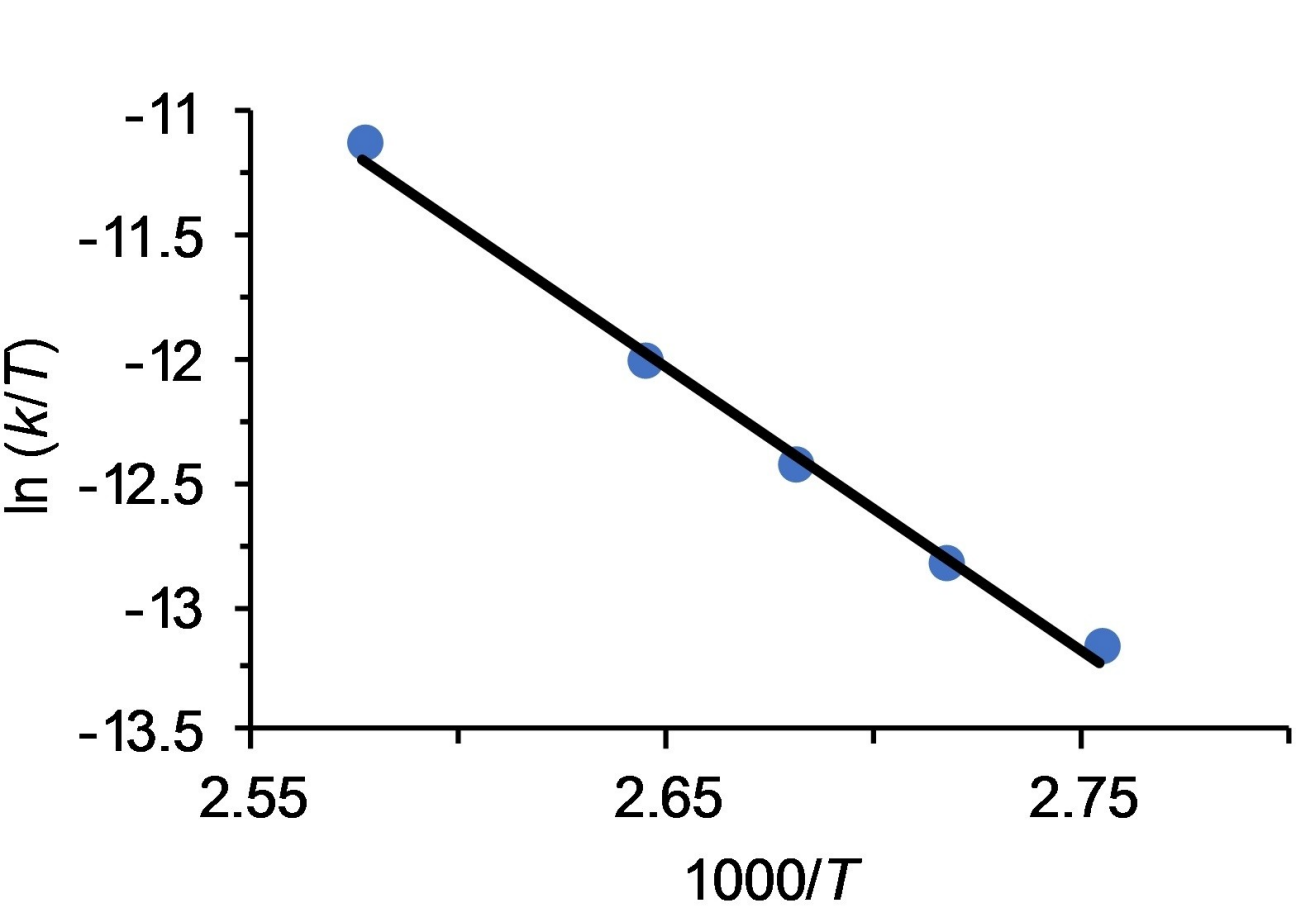
Eyring plot for the transformation of **3** into **5** in toluene.

The metathesis between the σ‐bonds Si−C(sp^*n*^) and C(sp^3^)−H of **2** (*n*=3) and **3** (*n*=2), to yield **4** and **5**, can be rationalized as a two‐step process. The first of them should involve the reductive elimination of R−H from **2** and **3**, to give tetrahydride‐osmium(IV)‐silylene intermediates OsH_4_(=SiR_2_)(P^i^Pr_3_)_2_ (**B** in Scheme 2). The reaction should be seen as the reverse process of a 1,2‐addition of ethane or benzene across the Os−Si bond of a silylene and could be favored by the great number of hydrides at the metal coordination sphere. A limited number of osmium(II)‐silylene compounds has been isolated,^[33]^ which were formed by α‐hydrogen elimination on secondary‐silyl intermediates. Once formed, the Os−Si double bond should then undergo the 1,2‐addition of a C(sp^3^)−H bond of a methyl group of an isopropyl substituent of one of the phosphine ligands, in the second step. To gain information about the metathesis, the conversion of **3** into **5** in toluene was followed by ^31^P{^1^H} NMR spectroscopy as a function of the time, between 363 and 388 K. In this temperature range, the silylene intermediate **B** was not detected suggesting that, for a mechanism as that shown in Scheme 2, the release of R−H should be the rate determining step of the σ‐bond metathesis, while the addition of the C(sp^3^)−H bond of the phosphine across the Os−Si double bond would be fast. Figure 3 shows the spectra of the transformation at 378 K. The consumption of **3** with the corresponding increase of the amount of **5** is an exponential function of time, which can be linearized (Figure 4) as a first‐order process, according to Equation 1:

(1) $$ln\frac{\left[3\right]}{{\left[3\right]}_{0}}=-kt$$

where [**3**]_0_ is the initial concentration of **3** and [**3**] is the concentration at time t. Values for the rate constant *k* in the temperature range studied are gathered in Table 1. The activation parameters obtained from the Eyring analysis (Figure 5) are Δ*H* ^≠^=22.8±3.3 kcal mol^−1^ and Δ*S* ^≠^=−14.2±8.8 cal K^−1^ mol^−1^, which combined give a Δ*G* ^≠^ value at 298 K of 27.1±5.9 kcal mol^−1^. The negative value of the activation entropy suggests an intramolecular process, which takes place via a highly ordered transition state, consistent with a direct elimination of R−H via a four‐center interaction.

**Table 1:** Rate constants for the transformation of **3** into **5** at different temperatures in toluene.

*T* [K]	[**3**]_0_ (M)	*k* [s^−1^]
363	0.032	(5.0± 0.5)×10^−4^
368	0.032	(1.0± 0.1)×10^−3^
373	0.032	(1.5± 0.2)×10^−3^
378	0.032	(2.3± 0.2)×10^−3^
388	0.032	(5.7± 0.6)×10^−3^

**Scheme 3:**
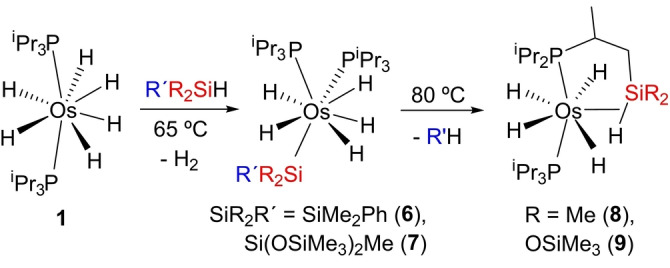
σ‐Activation/cross‐coupling metathesis according to Equation (c) extended to R'R_2_SiH: preparation of complexes **6–9**.

The metathesis between σ‐bonds summarized by Equation (c) can be extended to R′R_2_SiH tertiary silanes bearing two different substituents. The reactions show selectivity, which is controlled by the strength of the Si‐substituent bonds, releasing the hydrocarbon resulting from the rupture of the weakest Si‐substituent bond (Scheme 3), as expected for a process kinetically controlled by such release. According to this, complex **1** reacts with dimethylphenylsilane, and 1,1,1,3,5,5,5‐heptamethyltrisiloxane to initially give the pentahydride‐osmium(VI)‐silyl complexes OsH_5_(SiR_2_R′)(P^i^Pr_3_)_2_ (SiR_2_R′=SiMe_2_Ph (**6**), Si(OSiMe_3_)_2_Me (**7**)), which release benzene and methane to selectively and quantitatively afford the metathesis products, the osmium(IV)‐tetrahydride derivatives OsH_4_{κ^1^‐P,η^2^‐SiH‐[^i^Pr_2_PCH(Me)CH_2_SiR_2_H]}(P^i^Pr_3_) (R=Me (**8**), OSiMe_3_ (**9**)). NMR spectra of **6** and **7** agree well with those of **2** and **3**, whereas NMR spectra of **8** and **9** resemble those of **4** and **5** (see Experimental Section in Supporting Information).

**Scheme 4:**
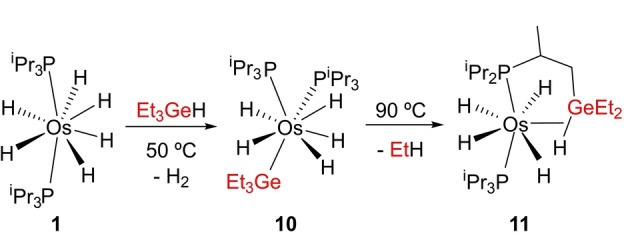
σ‐Activation/cross‐coupling metathesis according to Equation (c) extended to Et_3_GeH: preparation of complexes **10** and **11**.

Having demonstrated the generality of the σ‐ activation/cross‐coupling metathesis for silanes, we decided to investigate its extension to germanes. Treatment of solutions of **1** in toluene, at 50 °C, with 1.0 equiv of Et_3_GeH initially leads to complex OsH_5_(GeEt_3_)(P^i^Pr_3_)_2_ (**10**), the germyl counterpart of **2**, which as the latter loses ethane to give the σ‐bond metathesis product OsH_4_{κ^1^‐P,η^2^‐GeH‐[^i^Pr_2_PCH(Me)CH_2_GeEt_2_H]}(P^i^Pr_3_) (**11** in Scheme 4). NMR spectra of **10** in toluene‐*d*_8_ agree well with those of its silyl analogous compounds. Thus, the ^1^H spectrum at room temperature shows a hydride resonance at −10.40 (^2^ *J*_H,P_=9.0 Hz) ppm, which broadens as the sample temperature is lowered without undergoing decoalescence, whereas the ^31^P {^1^H} spectrum displays a singlet at 44.3 ppm. The NMR spectra of **11** in toluene‐*d*_8_ are similar to those of **4**, **5**, **8**, and **9**. In the ^1^H spectrum at room temperature, the most noticeable feature is a doublet of doublets at −11.0 ppm with H−P coupling constants of 14.5 and 9.3 Hz, whereas the ^31^P{^1^H} spectrum shows the characteristic AB spin system for this class of compounds at 69.2 ppm (Δ*ν*=3148 Hz and *J*_A–B_=138 Hz).

## Conclusion

This study shows the discovery of a new reaction, a metathesis between Si−C(sp^*n*^) and H−C(sp^3^) σ‐bonds (*n*=2, 3), which has taken place on the coordination sphere of the metal center of an osmium‐polyhydride. Osmium‐hexahydride complex OsH_6_(P^i^Pr_3_)_2_ reacts with R_3_SiH and R′R_2_SiH silanes to afford uncommon pentahydride‐osmium(VI)‐silyl intermediates, OsH_5_(SiR_3_)(P^i^Pr_3_)_2_ and OsH_5_(SiR_2_R′)(P^i^Pr_3_)_2_, which have been fully characterized. These d^2^‐species of a late transition metal evolve into the tetrahydride‐osmium(IV) derivatives OsH_4_{κ^1^‐P,η^2^‐SiH‐[^i^Pr_2_PCH(Me)CH_2_SiR_2_H]}, by releasing hydrocarbons R−H and R′−H, respectively. The release of the hydrocarbon is a consequence of the displacement of one of the substituents of the silyl ligand by a methyl group of an isopropyl substituent of a phosphine. The displacement displays selectivity when the silyl ligand bears different substituents, the hydrocarbon corresponding to the Si‐substituent bond weakest from the three being released. The reaction shows generality for silanes and has been extended to germanes.

## Conflict of interest

The authors declare no conflict of interest.

1

## Supporting information

As a service to our authors and readers, this journal provides supporting information supplied by the authors. Such materials are peer reviewed and may be re‐organized for online delivery, but are not copy‐edited or typeset. Technical support issues arising from supporting information (other than missing files) should be addressed to the authors.

Supporting Information

Supporting Information

Supporting Information

## Data Availability

The data that support the findings of this study are available in the Supporting Information of this article.
