# Supplementary material for: Metathesis between E−C(sp n ) and H−C(sp3) σ‐Bonds (E=Si, Ge; n=2, 3) on an Osmium‐Polyhydride
Source: Angew Chem Int Ed Engl. 2022 May 31;61(29):e202204081. doi: 10.1002/anie.202204081 (PMC9401005; doi:10.1002/anie.202204081)

checkCIF/PLATON report

Structure factors have been supplied for datablock(s) shelx

THIS REPORT IS FOR GUIDANCE ONLY. IF USED AS PART OF A REVIEW PROCEDURE FOR PUBLICATION, IT SHOULD NOT REPLACE THE EXPERTISE OF AN EXPERIENCED CRYSTALLOGRAPHIC REFEREE.

No syntax errors found.   CIF dictionary   Interpreting this report

Datablock: shelx

```
Bond precision:  C-C = 0.0050 A          Wavelength=0.71073

Cell:            a=8.9835(5)     b=21.1769(11)      c=19.054(1)
                 alpha=90        beta=95.848(1)     gamma=90
Temperature:     100 K

                Calculated                 Reported
Volume          3606.0(3)                  3606.0(3)
Space group     P 21/n                     P 21/n
Hall group      -P 2yn                     -P 2yn
Moiety formula  C36 H62 Os P2 Si           ?
Sum formula     C36 H62 Os P2 Si           C36 H62 Os P2 Si
Mr              775.12                     775.08
Dx,g cm-3       1.428                      1.428
Z               4                          4
Mu (mm-1)       3.681                      3.681
F000            1592.0                     1592.0
F000'           1588.73
h,k,lmax        12,28,25                   12,28,25
Nref            9425                       8965
Tmin,Tmax       0.579,0.629                0.727,0.862
Tmin'           0.568

Correction method= # Reported T Limits: Tmin=0.727 Tmax=0.862
AbsCorr = MULTI-SCAN

Data completeness= 0.951          Theta(max)= 28.808

R(reflections)= 0.0296( 6929)     wR2(reflections)= 0.0635( 8965)

S = 1.012                  Npar= 388
```

The following ALERTS were generated. Each ALERT has the format
test-name_ALERT_alert-type_alert-level.
Click on the hyperlinks for more details of the test.

Alert level C

```
PLAT971_ALERT_2_C Check Calcd Resid. Dens.  0.75A   From Os            2.27 eA-3
```

Alert level G

```
PLAT002_ALERT_2_G Number of Distance or Angle Restraints on AtSite         6 Note
PLAT083_ALERT_2_G SHELXL Second Parameter in WGHT  Unusually Large      5.34 Why ?
PLAT172_ALERT_4_G The CIF-Embedded .res File Contains DFIX Records         1 Report
PLAT720_ALERT_4_G Number of Unusual/Non-Standard Labels .........          5 Note
PLAT860_ALERT_3_G Number of Least-Squares Restraints .............         5 Note
PLAT883_ALERT_1_G No Info/Value for _atom_sites_solution_primary .    Please Do !
PLAT912_ALERT_4_G Missing # of FCF Reflections Above STh/L=  0.600       459 Note
PLAT965_ALERT_2_G The SHELXL WEIGHT Optimisation has not Converged    Please Check
PLAT978_ALERT_2_G Number C-C Bonds with Positive Residual Density.         3 Info
```

0 **ALERT level A** = Most likely a serious problem - resolve or explain
0 **ALERT level B** = A potentially serious problem, consider carefully
1 **ALERT level C** = Check. Ensure it is not caused by an omission or oversight
9 **ALERT level G** = General information/check it is not something unexpected

1 ALERT type 1 CIF construction/syntax error, inconsistent or missing data
5 ALERT type 2 Indicator that the structure model may be wrong or deficient
1 ALERT type 3 Indicator that the structure quality may be low
3 ALERT type 4 Improvement, methodology, query or suggestion
0 ALERT type 5 Informative message, check

It is advisable to attempt to resolve as many as possible of the alerts in all categories. Often the minor alerts point to easily fixed oversights, errors and omissions in your CIF or refinement strategy, so attention to these fine details can be worthwhile. In order to resolve some of the more serious problems it may be necessary to carry out additional measurements or structure refinements. However, the purpose of your study may justify the reported deviations and the more serious of these should normally be commented upon in the discussion or experimental section of a paper or in the "special_details" fields of the CIF. checkCIF was carefully designed to identify outliers and unusual parameters, but every test has its limitations and alerts that are not important in a particular case may appear. Conversely, the absence of alerts does not guarantee there are no aspects of the results needing attention. It is up to the individual to critically assess their own results and, if necessary, seek expert advice.

Publication of your CIF in IUCr journals

A basic structural check has been run on your CIF. These basic checks will be run on all CIFs submitted for publication in IUCr journals (*Acta Crystallographica*, *Journal of Applied Crystallography*, *Journal of Synchrotron Radiation*); however, if you intend to submit to *Acta Crystallographica Section C* or *E* or *IUCrData*, you should make sure that full publication checks are run on the final version of your CIF prior to submission.

Publication of your CIF in other journals

Please refer to the *Notes for Authors* of the relevant journal for any special instructions relating to CIF submission.

PLATON version of 16/05/2021; check.def file version of 13/05/2021

Datablock shelx - ellipsoid plot

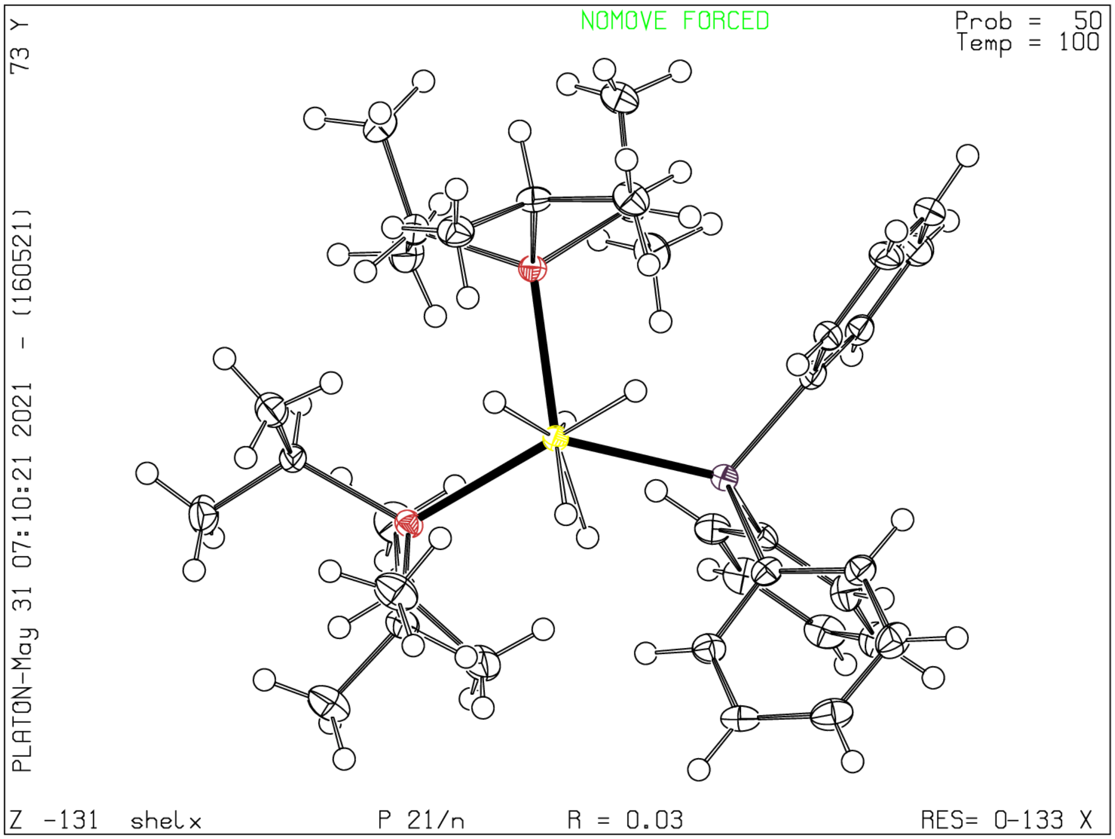

checkCIF/PLATON report

Structure factors have been supplied for datablock(s) shelx

THIS REPORT IS FOR GUIDANCE ONLY. IF USED AS PART OF A REVIEW PROCEDURE FOR PUBLICATION, IT SHOULD NOT REPLACE THE EXPERTISE OF AN EXPERIENCED CRYSTALLOGRAPHIC REFEREE.

No syntax errors found. CIF dictionary Interpreting this report

Datablock: shelx

```
Bond precision:  C-C = 0.0037 A          Wavelength=0.71073

Cell:           a=17.4758(15)    b=9.7446(8)        c=20.5035(18)
                alpha=90         beta=115.071(1)    gamma=90
Temperature:    120 K

                Calculated                  Reported
Volume          3162.7(5)                   3162.7(5)
Space group     P 21/n                      P 21/n
Hall group      -P 2yn                      -P 2yn
Moiety formula  C30 H56 Os P2 Si            ?
Sum formula     C30 H56 Os P2 Si            C30 H56 Os P2 Si
Mr              697.01                      696.97
Dx,g cm-3       1.464                       1.464
Z               4                           4
Mu (mm-1)       4.188                       4.188
F000            1424.0                      1424.0
F000'           1420.71
h,k,lmax        24,13,28                    24,13,28
Nref            9167                        8820
Tmin,Tmax       0.487,0.718                 0.742,0.862
Tmin'           0.462

Correction method= # Reported T Limits: Tmin=0.742 Tmax=0.862
AbsCorr = MULTI-SCAN

Data completeness= 0.962          Theta(max)= 29.945

R(reflections)= 0.0205( 7460)     wR2(reflections)= 0.0475( 8820)

S = 1.013                  Npar= 333
```

The following ALERTS were generated. Each ALERT has the format
test-name_ALERT_alert-type_alert-level.
Click on the hyperlinks for more details of the test.

Alert level C

```
PLAT094_ALERT_2_C Ratio of Maximum / Minimum Residual Density ....       3.24 Report
PLAT222_ALERT_3_C NonSolvent Resd 1  H   Uiso(max)/Uiso(min) Range        4.1 Ratio
PLAT977_ALERT_2_C Check Negative Difference Density on H01              -0.39 eA-3
PLAT977_ALERT_2_C Check Negative Difference Density on H02              -0.38 eA-3
```

Alert level G

```
PLAT002_ALERT_2_G Number of Distance or Angle Restraints on AtSite          6 Note
PLAT172_ALERT_4_G The CIF-Embedded .res File Contains DFIX Records          1 Report
PLAT232_ALERT_2_G Hirshfeld Test Diff (M-X)  Os       --P2       .        5.5 s.u.
PLAT303_ALERT_2_G Full Occupancy Atom H01       with # Connections       2.00 Check
PLAT720_ALERT_4_G Number of Unusual/Non-Standard Labels ..........          5 Note
PLAT779_ALERT_4_G Suspect or Irrelevant (Bond) Angle(s) in CIF ...      40.00 Deg.
              OS   -SI   -H01     1_555   1_555   1_555 ......     #   50 Check
PLAT793_ALERT_4_G Model has Chirality at C1          (Centro SPGR)          R Verify
PLAT860_ALERT_3_G Number of Least-Squares Restraints .............          5 Note
PLAT883_ALERT_1_G No Info/Value for _atom_sites_solution_primary .     Please Do !
PLAT912_ALERT_4_G Missing # of FCF Reflections Above STh/L=  0.600        348 Note
PLAT965_ALERT_2_G The SHELXL WEIGHT Optimisation has not Converged     Please Check
PLAT978_ALERT_2_G Number C-C Bonds with Positive Residual Density.          5 Info
```

0 **ALERT level A** = Most likely a serious problem - resolve or explain
0 **ALERT level B** = A potentially serious problem, consider carefully
4 **ALERT level C** = Check. Ensure it is not caused by an omission or oversight
12 **ALERT level G** = General information/check it is not something unexpected

1 ALERT type 1 CIF construction/syntax error, inconsistent or missing data
8 ALERT type 2 Indicator that the structure model may be wrong or deficient
2 ALERT type 3 Indicator that the structure quality may be low
5 ALERT type 4 Improvement, methodology, query or suggestion
0 ALERT type 5 Informative message, check

It is advisable to attempt to resolve as many as possible of the alerts in all categories. Often the minor alerts point to easily fixed oversights, errors and omissions in your CIF or refinement strategy, so attention to these fine details can be worthwhile. In order to resolve some of the more serious problems it may be necessary to carry out additional measurements or structure refinements. However, the purpose of your study may justify the reported deviations and the more serious of these should normally be commented upon in the discussion or experimental section of a paper or in the "special_details" fields of the CIF. checkCIF was carefully designed to identify outliers and unusual parameters, but every test has its limitations and alerts that are not important in a particular case may appear. Conversely, the absence of alerts does not guarantee there are no aspects of the results needing attention. It is up to the individual to critically assess their own results and, if necessary, seek expert advice.

Publication of your CIF in IUCr journals

A basic structural check has been run on your CIF. These basic checks will be run on all CIFs submitted for publication in IUCr journals (*Acta Crystallographica*, *Journal of Applied Crystallography*, *Journal of Synchrotron Radiation*); however, if you intend to submit to *Acta Crystallographica Section C* or *E* or *IUCrData*, you should make sure that full publication checks are run on the final version of your CIF prior to submission.

Publication of your CIF in other journals

Please refer to the *Notes for Authors* of the relevant journal for any special instructions relating to CIF submission.

PLATON version of 16/05/2021; check.def file version of 13/05/2021

Datablock shelx - ellipsoid plot

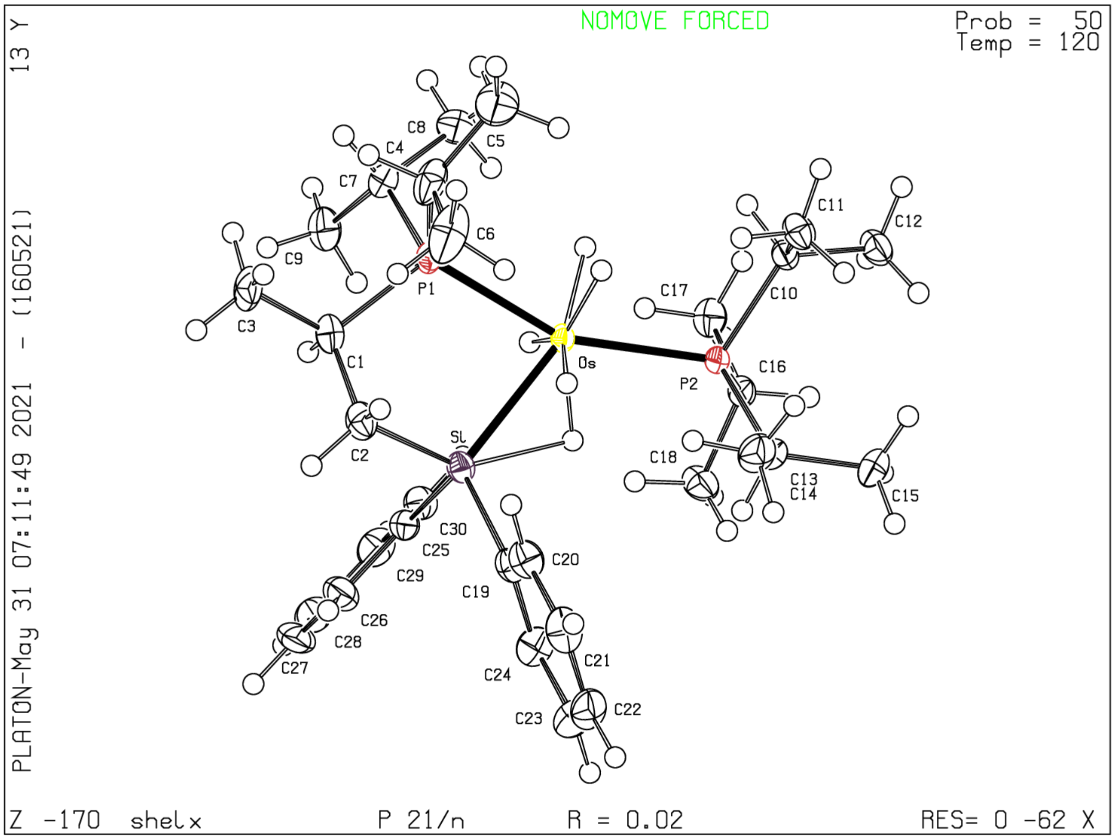

Supplement: Supplementary file 1 — Supporting Information [file ANIE-61-0-s003.pdf]
