# Supplementary material for: Metathesis between E−C(sp n ) and H−C(sp3) σ‐Bonds (E=Si, Ge; n=2, 3) on an Osmium‐Polyhydride
Source: Angew Chem Int Ed Engl. 2022 May 31;61(29):e202204081. doi: 10.1002/anie.202204081 (PMC9401005; doi:10.1002/anie.202204081)
Supplement: Supplementary file 3 — Supporting Information [file ANIE-61-0-s002.pdf]

Angewandte Chemie
Eine Zeitschrift der Gesellschaft Deutscher Chemiker

Supporting Information

Metathesis between E–C(sp^{n}) and H–C(sp^{3}) σ-Bonds (E = Si, Ge; n = 2, 3) on an Osmium-Polyhydride

M. A. Esteruelas, A. M. López, E. Oñate, E. Raga*

Supporting Information

Contents:

- Experimental Section:

General Information …………………………………………………………..…… S2
Preparation of Complexes **2**-**11** ………………………………………………… S2
NMR spectroscopy Study of the transformation of **3** into **5** ……………............ S9
Kinetic Plots (Figure S1) ……………………………………………...…….. S10

- Structural Analysis of Complexes $OsH_5(SiPh_3)(P^iPr_3)_2$ (**3**) and
$OsH_4\{\kappa^1\text{-}P,\eta^2\text{-}SiH\text{-}[^iPr_2PCH(Me)CH_2SiPh_2H]\}(P^iPr_3)$ (**5**) ……………………… S11

- Computational details ……………………………………………………...….... S12

- References ………………………………………………………………….…….. S13

- X-ray and DFT structures of **3** and **5** (Figures S2-S3) ……………………..……... S14

- Contour line diagram $\nabla^2\rho(r)$ for complex **5** (Figure S4) ……………………….… S15

- NMR Spectra (Figures S5-S48) ……………..…………..……………………...… S16

Experimental Section: General Information. All reactions were carried out with rigorous exclusion of air using Schlenk-tube or glovebox techniques. Solvents were dried by the usual procedures and distilled under argon prior to use or obtained oxygen- and water-free from an MBraun solvent purification apparatus. n-Octane was stored over P_2O_5 in the glovebox. C, H, and N analyses were carried out in a Perkin-Elmer 2400B SeriesII-Analyzer. High-resolution (HRMS) were acquired using a MicroTOF-Q hybrid quadrupole time-of-flight spectrometer (Bruker Daltonics, Bremen, Germany). IR spectra were measured using a PerkinElmer Spectrum 100 FT-IR spectrometer, equipped with an ATR accessory, as pure solids or oils. ^{1}H, ^{13}C{^{1}H}, and ^{31}P{^{1}H} NMR spectra were recorded on a Bruker Avance 300, 400, or 500 MHz instrument. ^{29}Si spectra were recorded on a Bruker Avance 300 MHz instrument. Chemical shifts (expressed in parts per million) are referenced to residual solvent peaks. Coupling constants, J and N ($N = {}^{3}J_{H,P} + {}^{5}J_{H,P'}$ for ^{1}H or ${}^{1}J_{C,P} + {}^{3}J_{C,P'}$ for ^{13}C), are given in Hertz. Complex $OsH_6(P^iPr_3)_2$ (**1**) was prepared according to the published method.[1]

Reaction of 1 with Et$_3$SiH: Formation of OsH$_5$(SiEt$_3$)(P^{i}Pr$_3$)$_2$ (2).

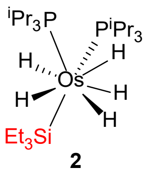

2

A solution of **1** (50 mg, 0.10 mmol) and Et_3SiH (16 μL, 0.10 mmol) in n-octane (4 mL) was heated at 65 ºC, for 4 h, in an Schlenk-tube attached to a condenser provided with a gas bubbler. Afterwards, the crude reaction was concentrated to dryness under reduced pressure, giving a yellow oil. The NMR spectra showed the formation of **2** and **6** in a 70:30 molar ratio. NMR data for **2**: ^{1}H NMR (300.13 MHz, C_6D_6, 298 K): δ 1.90 (m, 6H, PCH), 1.30 (t, $^{3}J_{H,H}$= 6.7, 9H, $SiEt_3$), 1,12 (m, 42H, $PCCH_3$ + $SiEt_3$), −10.70 (t, $^{2}J_{H,P}$ = 6.4, 5H, Os-H). ^{31}P{^{1}H} NMR (121.50 MHz, C_6D_6, 298 K): δ 38.7 (s). ^{13}C{^{1}H}-apt NMR (75 MHz, C_6D_6, 298 K): 29.5 (PCH partially overlapped with PCH of **6**), 20.2 (s, PC*C*H$_3$), 16.7 (s, Si*C*H$_2$CH$_3$), 9.9 (s, SiCH$_2$*C*H$_3$). ^{29}Si{^{1}H}NMR (59.63 MHz, C_6D_6, 298 K): δ -1.4 (t, $^{2}J_{Si,P}$ = 4.9). $T_{1(min)}$ (ms, OsH_5, 400 MHz, toluene-d_8, 228 K): 176 ± 18 (−10.70 ppm). HRMS (electrospray, *m/z*): calcd. for $C_{24}H_{62}NaOsP_2Si$ [M+Na]$^+$: 655.3603; found 655.3584.

Preparation of $OsH_5(SiPh_3)(P^iPr_3)_2$ (3).

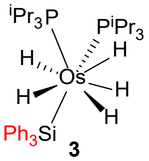

3

A solution of **1** (100 mg, 0.19 mmol) and Ph_3SiH (49.5 mg, 0.19 mmol) in n-octane (4 mL) was heated at 65 ºC, for 4 h, in an Schlenk-tube attached to a condenser provided with a gas bubbler. Afterwards, the resulting suspension was cooled to room temperature and the solvent was evaporated under reduced pressure until ca. 2 mL. A white solid was separated by decantation, washed with pentane at 0 ºC (3 x 2 mL), and dried in vacuo. Yield: 99 mg (66 %). Colorless single crystals suitable for XRD analysis were grown from a solution of **3** in acetone at −30 °C. ^{1}H NMR (300.13 MHz, C_6D_6, 298 K): δ 8.10 (dd, $^3J_{H,H}$ = 8.1, $^4J_{H,H}$ = 1.4, 6H, *o*-Ph), 7.24 (m, 6H, *m*-Ph), 7.12 (m, 3H, *p*-Ph), 1.59 (m, 6H, PCH), 0.99 (dvt, $^3J_{H,H}$ = 7.2, *N* = 13.5, 36H, $PCCH_3$), −9.67 (t, $^2J_{H,P}$ = 8.4, 5H, Os-H). ^{31}P{^{1}H} NMR (121.50 MHz, C_6D_6, 298 K): δ 39.4 (s). ^{13}C{^{1}H}-apt NMR (75 MHz, C_6D_6, 298 K): δ 148.2 (s, C_q Ph), 137.8 (s, CH Ph), 127.4 (s, *p*-CH Ph), 126.7 (s, CH Ph), 29.0 (vt, *N* = 28.0, PCH), 20.0 (s, $PC\mathit{C}H_3$). ^{29}Si{^{1}H}NMR (59.63 MHz, C_6D_6, 298 K): δ -2.4 (t, $^2J_{Si,P}$ = 3.9). $T_{1(min)}$ (ms, OsH_5, 300.13 MHz, toluene-d_8, 228 K): 138 ± 14 (−9.67 ppm). IR (cm^{-1}): ν(Os-H) 1972 (w). HRMS (electrospray, *m/z*): calcd. for $C_{36}H_{62}NaOsP_2Si$ $[M+Na]^+$: 799.3604; found 799.3531. Anal. Calcd. for $C_{36}H_{62}OsP_2Si$: C, 55.78; H, 8.06. Found: C, 56.03; H, 8.24.

Preparation of $OsH_4\{\kappa^1\text{-}P,\eta^2\text{-}SiH\text{-}[^iPr_2PCH(Me)CH_2SiEt_2H]\}(P^iPr_3)$ (4).

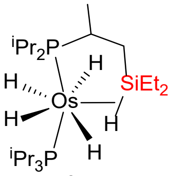

4

A solution of **1** (50 mg, 0.10 mmol) in toluene (4 mL) was treated with Et_3SiH (16 µL, 0.10 mmol). The resulting mixture was heated at 90 ºC for 6h, in an Schlenk-tube attached to a condenser provided with a gas bubbler. Afterwards, the reaction crude was concentrated to

dryness under reduced pressure, giving a yellow oil. The ^{1}H and ^{31}P NMR spectra showed the quantitative formation of **4**. ^{1}H NMR (500.12 MHz, C_6D_6, 298 K): 1.81 (m, 6H, PCH), 1.50 (ddd, $^{2}J_{H,H}$ = 13.5, $^{3}J_{H,H}$ = 6.2, $^{3}J_{H,P}$ = 49.4, 1H, PCCH_2Si), 1.30 (m, 10H, $SiEt_2$), 1.20 (m, 6H, $PCCH_3$), 1.10 (dd, $^{3}J_{H,H}$ = 7.1, $^{3}J_{H,P}$ = 13.7, 9H, $PCCH_3$ P^iPr_3), 1.07 (dd, $^{3}J_{H,H}$ = 7.1, $^{3}J_{H,P}$ = 13.7, 9H, $PCCH_3$ P^iPr_3), 1.05-0.93 (m, 9H, $PCCH_3$), 0.80 (ddd, $^{2}J_{H,H}$ = $^{3}J_{H,H}$ =13.5, $^{3}J_{H,P}$ = 5.6, 1H, PCCH_2Si), −10.85 (dd, $^{2}J_{H,P}$ = 13.8, 10.6, 5H, Os-H). ^{31}P{^{1}H} NMR (121.50 MHz, C_6D_6, 298 K): δ 64.6 (AB spin system, Δν = 2541 Hz, $J_{A\text{-}B}$ = 152.0). ^{13}C{^{1}H}-apt NMR (125.77 MHz, C_6D_6, 298 K): δ 32.6 (d, $J_{C,P}$ = 31.2, PCH P^iPr_2), 30.7 (dd, $J_{C,P}$ = 31.2, 1.6, PCH P^iPr_2), 30.3 (dd, $J_{C,P}$ = 30.1, 2.0, PCCH_2Si), 29.3 (dd, $J_{C,P}$ = 27.9, 1.7, PCH P^iPr_3), 26.6 (dd, $J_{C,P}$ = 27.3, 1.9, PCHCH_2Si), 21.6 (d, $^{2}J_{C,P}$ = 1.3, PCCH_3), 20.6 (d, $^{2}J_{C,P}$ = 1.4, PCCH_3), 20.6, 20.5 (both singlets, PCCH_3 P^iPr_3), 19.6 (s, PCCH_3), 18.5 (d, $^{2}J_{C,P}$= 1.7, PCCH_3), 18.4 (d, $^{2}J_{C,P}$ = 2.9, PCCH_3), 18.0, 17.6 (both s, SiCH_2CH_3), 10.3, 10.1 (both s, SiCH_2CH_3). ^{29}Si{^{1}H}NMR (59.63 MHz, C_6D_6, 298 K): δ 26.1 (dd, $J_{Si,P}$ = 18.2, 13.9). $T_{1(min)}$ (ms, OsH_5, 300.13 MHz, toluene-d_8, 203 K): 154 ± 15 (−10.85 ppm). IR (cm^{-1}): ν(Os-H) 1993, 1913 (w). HRMS (electrospray, *m/z*): calcd. for $C_{22}H_{56}NaOsP_2Si$ [M+Na]$^{+}$: 625.3133. Found: 625.3109.

Preparation of OsH_4\{κ^1-*P*,η^2-*SiH*-[$^iPr_2PCH(Me)CH_2SiPh_2H$]\}(P^iPr_3) (5).

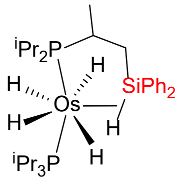

5

A solution of **3** (50 mg, 0.065 mmol) in toluene (4 mL) was stirred in a sealed tube at 90 ºC for 6 h. The crude reaction mixture was concentrated to dryness under reduced pressure, giving a yellow oil. The ^{1}H and ^{31}P{^{1}H} NMR spectra showed the quantitative formation of **5**. Colorless single crystals suitable for XRD analysis were grown from a solution of **5** in pentane at -30 ºC. Yield: 29 mg (65 %). ^{1}H NMR (300.13 MHz, C_6D_6, 298 K): δ 8.12 (m, 2H, *o*-Ph), 7.83 (m, 2H, *o*-Ph), 7.40 (m, 2H, *m*-Ph), 7.30 (m, 2H, *m*-Ph), 7.21 (m, 1H, *p*-Ph), 7.13 (m, 1H, *p*-Ph), 2.20 (ddd, $^{2}J_{H,H}$ = 13.6, $^{3}J_{H,H}$ = 5.8, $^{3}J_{H\text{-}P}$ = 50.6, 1H, PCCH_2Si), 1.83 (m, 3H, PCH), 1.70 (m, 3H, PCH P^iPr_3), 1.40 (ddd, $^{2}J_{H,H}$ = $^{3}J_{H,H}$ =13.6, $^{3}J_{H,P}$ = 4.8, 1H, PCCH_2Si),

1.10 (m, 12H, PCCH$_3$), 0.96 (dd, $^3J_{H,H}$ = 7.0, $^3J_{H,P}$ = 13.9, 9H, PCCH$_3$ P^{i}Pr$_3$), 0.95 (dd, $^3J_{H,H}$ = 7.0, $^3J_{H,P}$ = 13.9, 9H, PCCH$_3$ P^{i}Pr$_3$), 0.90 (m, 3H, PCCH$_3$), −10.18 (dd, $^2J_{H,P}$ = 13.4, 10.1, 5H, Os-H). ^{31}P{^{1}H} NMR (121.50 MHz, C_6D_6, 298 K): δ 63.8 (AB spin system, Δν = 2693 Hz, $J_{A\text{-}B}$ = 148 Hz). ^{13}C{^{1}H} NMR (75 MHz, C_6D_6, 298 K): δ 149.0, 146.3 (both s, C_q Ph), 136.6, 136.5, 135.3, 135.2, 127.3, 127.1 (all s, CH Ph), 35.3 (dd, $J_{C,P}$ = 28.3, 2.3, PC*C*H$_2$Si), 30.9 (dd, $J_{C,P}$ = 31.0, 1.7, PCH P^{i}Pr$_2$), 30.1 (d, $J_{C,P}$ = 25.5, PCH P^{i}Pr$_2$), 29.1 (dd, $J_{C,P}$ = 28.3, 1.8, PCH P^{i}Pr$_3$), 26.0 (dd, $J_{C,P}$ = 28.1, 1.9, P*C*HCH$_2$Si), 21.5 (d, $^2J_{C,P}$ = 1.7, PC*C*H$_3$), 20.5 (d, $^2J_{C,P}$ = 1.4, PC*C*H$_3$), 20.3, 20.2 (both singlets, PC*C*H$_3$ P^{i}Pr$_3$), 19.4 (s, PC*C*H$_3$), 18.3 (d, $J_{C,P}$ = 2.6, PC*C*H$_3$), 18.1 (d, $J_{C,P}$ = 3.0, PC*C*H$_3$). ^{29}Si{^{1}H}NMR (59.63 MHz, C_6D_6, 298 K): δ 18.7 (dd, $J_{Si,P}$ = 19.6, 15.5). $T_{1(min)}$ (ms, OsH$_5$, 300.13 MHz, toluene-d_8, 213 K): 154 ± 15 (−10.18 ppm). IR (cm^{-1}): ν(Os-H) 1992, 1919 (w). HRMS (electrospray, *m/z*): calcd. for $C_{30}H_{55}OsP_2Si$ [M-H]$^+$: 697.3158; found 697.3133. Anal. Calcd. for $C_{30}H_{56}OsP_2Si$: C, 51.70; H, 8.10. Found: C, 52.03; H, 8.40.

Reaction of 1 with PhMe$_2$SiH: Formation of OsH$_5$(SiMe$_2$Ph)(P^{i}Pr$_3$)$_2$ (6).

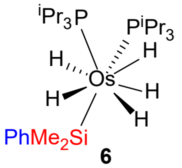

6

A solution of **1** (100 mg, 0.19 mmol) and PhMe$_2$SiH (45 μL, 0.29 mmol) in n-octane (4 mL) was heated at 60 ºC, for 5 h, in an Schlenk-tube attached to a condenser provided with a gas bubbler. Afterwards, the crude reaction was concentrated to dryness under reduced pressure, giving a yellow oil. The NMR spectra showed the presence of **1** and **6** in a 10:90 molar ratio. NMR data for **6**: ^{1}H NMR (300.13 MHz, C_6D_6, 298 K): δ 7.91 (dd, $^3J_{H,H}$ = 8.0, $^4J_{H,H}$ = 1.4, 2H, *o*-Ph), 7.31 (m, 2H, *m*-Ph), 7.14 (m, 1H, *p*-Ph), 1.80 (m, 6H, PCH), 1.09 (s, 6H, *Me$_2$*SiPh), 1.05 (dvt, $^3J_{H,H}$ = 6.9, *N* = 13.5, 36H, PCCH$_3$), −10.13 (t, $^2J_{H,\text{-}P}$ = 9.2, 5H, Os-H). ^{31}P{^{1}H} NMR (121.50 MHz, C_6D_6, 298 K): δ 43.8 (s). ^{13}C{^{1}H}-apt NMR (75 MHz, C_6D_6, 298 K): δ 152.9 (s, C_q Ph), 136.1 (s, CH *p*-Ph), 134.2 (s, *o*-CH Ph), 126.9 (s, CH *m*-Ph), 29.2 (vt, *N* = 29.0, PCH), 20.1 (s, PC*C*H$_3$), 15.1 (s, *Me$_2$*SiPh). ^{29}Si{^{1}H}NMR (59.63 MHz, C_6D_6, 298 K): δ -21.8 (t, $^2J_{Si,P}$ = 1.5). $T_{1(min)}$ (ms, OsH$_5$, 300.13 MHz, toluene-d_8, 228 K): 152 ± 15

(−10.13 ppm). IR (cm^{-1}): ν(Os-H) 1894 (w). HRMS (electrospray, *m/z*): calcd. for $C_{26}H_{58}NaOsP_2Si$ $[M+Na]^+$: 675.3290; found 675.3305.

Preparation of $OsH_5\{Si(OSiMe_3)_2Me\}(P^iPr_3)_2$ (7).

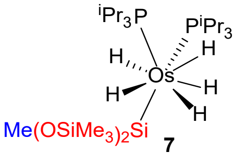

7

A solution of **1** (50 mg, 0.10 mmol) in octane (4 mL) was treated with $Me(OSiMe_3)_2SiH$ (27 μL, 0.10 mmol). The resulting mixture was heated at 65 ºC for 4 h, in an Schlenk-tube attached to a condenser provided with a gas bubbler. The crude reaction was concentrated to dryness under reduced pressure, giving a yellow oil. The ^{1}H and ^{31}P NMR spectra showed the quantitative formation of **7**. ^{1}H NMR (300.13 MHz, C_6D_6, 298 K): δ 2.03 (m, 6H, PCH), 1.14 (dvt, $^3J_{H,H}$ = 6.9, N = 13.5, 36H, $PCCH_3$), 0.75 (s, 3H, *Me*Si(OSiMe$_3$)$_2$), 0.40 (s, 18H, MeSi(OSi*Me*$_3$)$_2$), −10.31 (t, $^2J_{H,P}$ = 8.5, 5H, Os-H). ^{31}P{^{1}H} NMR (121.50 MHz, C_6D_6, 298 K): δ 45.5 (s). ^{13}C{^{1}H}-apt NMR (75 MHz, C_6D_6, 298 K): δ 29.0 (vt, N = 28.6, PCH), 23.8 (s, *Me*Si(OSiMe$_3$)$_2$), 20.2 (s, PC*C*H$_3$), 3.0 (s, OSiMe$_3$). ^{29}Si{^{1}H}NMR (59.63 MHz, C_6D_6, 298 K): δ −6.0 (s, OSiMe$_3$), −14.9 (t, $^2J_{Si,P}$ = 5.0, Os-Si). $T_{1(min)}$ (ms, OsH_5, 300.13 MHz, toluene-d_8, 203 K): 140 ± 14 (−10.31 ppm). IR (cm^{-1}): ν(Os-H) 1894. HRMS (electrospray, *m/z*): calcd. for $C_{25}H_{67}NaO_2OsP_2Si_3$ $[M-H+Na]^+$: 760.3428; found 760.3394.

Reaction of 1 with $HSiMe_2Ph$: Formation of $OsH_4\{\kappa^1\text{-}P,\eta^2\text{-}SiH\text{-}[^iPr_2PCH(Me)CH_2SiMe_2H]\}(P^iPr_3)$ (8).

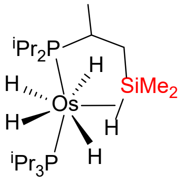

8

A solution of **1** (50 mg, 0.10 mmol) in toluene (4 mL) was treated with $PhMe_2SiH$ (23.0 μL, 0.15 mmol). The resulting mixture was heated at 80 ºC for 8 h, in an Schlenk-tube attached

to a condenser provided with a gas bubbler. Afterwards, the reaction crude was concentrated to dryness under reduced pressure, giving a yellow oil. The ^{1}H and ^{31}P NMR spectra showed the presence of **1** and **8** in a 8:92 molar ratio. ^{1}H NMR (300.13 MHz, C_6D_6, 298 K): δ 2.00-1.66 (m, 6H, PCH), 1.50 (ddd, $^{2}J_{H,H}$ = 12.9, $^{3}J_{H,H}$ = 5.8, $^{3}J_{H,P}$ = 48.5, 1H, PCC*H*$_2$Si), 1.30-0.95 (m, 33H, $PCCH_3$), 1.00 (s, 3H, $SiMe_2$), 0.90 (s, 3H, $SiMe_2$), 0.83 (m, 1H, PCHC*H*$_2$Si), −10.70 (dd, $^{2}J_{H,P}$ = 13.8, 10.8, 5H, Os-H. ^{31}P{^{1}H} NMR (121.50 MHz, C_6D_6, 298 K): δ 66.2 (AB spin system, Δν = 2535, $J_{A\text{-}B}$ = 156). ^{13}C{^{1}H}-apt NMR (75 MHz, C_6D_6, 298 K): δ 35.9 (d, $^{2}J_{C,P}$ = 29.5, PC*C*H$_2$Si), 31.7 (d, $J_{C,P}$ = 25.3, PCH P^{i}Pr$_2$), 30.8 (d, $J_{C,P}$ = 30.1, PCH P^{i}Pr$_2$), 29.1 (d, $J_{C,P}$ = 29.4, PCH P^{i}Pr$_3$), 26.5 (d, $J_{C,P}$ = 27.0, P*C*HCH$_2$Si), 21.5, 20.7 (both s, PC*C*H$_3$), 20.6 (s, PC*C*H$_3$ P^{i}Pr$_3$), 19.5 (s, PC*C*H$_3$), 18.4 (s, 2 PC*C*H$_3$), 14.1, 14.0 (both s, $SiMe_2$). ^{29}Si{^{1}H}NMR (59.63 MHz, C_6D_6, 298 K): δ 10.0 (dd, $J_{Si,P}$ = 18.0, 14.7). $T_{1(min)}$ (ms, OsH_5, 300.13 MHz, toluene-d_8, 203 K): 166 ± 17 (−10.70 ppm). IR (cm^{-1}): ν(Os-H) 1909, 1992 (w). HRMS (electrospray, *m/z*) calcd. for $C_{20}H_{52}NaOsP_2Si$ [M+Na]$^+$: 597.2820; found 597.2799.

Preparation of OsH$_4${*κ*^{1}-*P*,*η*^{2}-*SiH*-[^{i}Pr$_2$PCH(Me)CH$_2$Si(OSiMe$_3$)$_2$H]}(P^{i}Pr$_3$) (9).

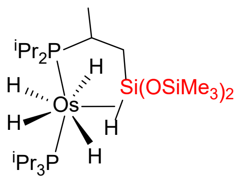

9

A solution of **7** (50 mg, 0.068 mmol) in toluene (4 mL) was stirred in a sealed tube at 80 ºC for 6 h. The crude reaction was concentrated to dryness under reduced pressure, giving a yellow oil. The ^{1}H and ^{31}P NMR spectra showed the quantitative formation of **9**. ^{1}H NMR (300.13 MHz, C_6D_6, 298 K): δ 1.96-1.70 (m, 6H, PCH), 1.50 (ddd, $^{2}J_{H,H}$ = 12.9, $^{3}J_{H,H}$ = 6.2, $^{3}J_{H,P}$ = 46.9, 1H, PCC*H*$_2$Si), 1.29-1.18 (m, 9H, $PCCH_3$), 1.12 (dd, $^{3}J_{H,P}$ = 13.7, $^{3}J_{H,H}$ = 7.0, 18H, $PCCH_3$ P^{i}Pr$_3$), 1.03-0.96 (m, 6H, $PCCH_3$), 0.81 (ddd, $^{2}J_{H,H}$ = $^{3}J_{H,H}$ =13.0, $^{3}J_{H,P}$ = 6.7, 1H, PCHC*H*$_2$Si), 0.36 (s, 9H, $OSiMe_3$), 0.35 (s, 9H, $OSiMe_3$), −10.58 (dd, $^{2}J_{H,P}$ = 13.3, 11.3, 5H, Os-H). ^{31}P{^{1}H} NMR (121.50 MHz, C_6D_6, 298 K): δ 65.6 (AB spin system, Δν = 2869, $J_{A\text{-}B}$ = 159.0). ^{13}C{^{1}H}-apt NMR (75 MHz, C_6D_6, 298 K): δ 44.1 (dd, $J_{C,P}$ = 28.8, 4.3,

$PCCH_2Si$), 30.9 (dd, $J_{C,P}$ = 31.2, 1.6, PCH), 29.0 (dd, $J_{C,P}$ = 27.8, 1.6, PCH P^iPr_3), 26.4 (d, $J_{C,P}$ = 24.5, PCH), 26.2 (dd, $J_{C,P}$ = 28.5, 2.0, PCH), 21.4 (d, $^2J_{C,P}$ = 1.4, $PCCH_3$), 20.7 (d, $^2J_{C,P}$ = 1.2, $PCCH_3$), 20.6, 20.5 (both singlets, $PCCH_3$ P^iPr_3), 19.5 (s, $PCCH_3$), 18.4 (d, $^2J_{C,P}$ = 1.8, $PCCH_3$), 17.5 (d, $^2J_{C,P}$ = 3.0, $PCCH_3$), 2.8, 2.7 (both s, $OSiMe_3$). $^{29}Si\{^1H\}$NMR (59.63 MHz, C_6D_6, 298 K): δ 5.4 (dd, $J_{Si,P}$ = 23.6, 18.8, Os-Si), 4.4, 3.7 (both s, $OSiMe_3$). $T_{1(min)}$ (ms, OsH_5, 300.13 MHz, toluene-d_8, 213 K): 164 ± 16 (−10.58 ppm). IR (cm^{-1}): ν(Os-H) 1924 (w). HRMS (electrospray, *m/z*): calcd. for $C_{24}H_{64}NaO_2OsP_2Si_3$ $[M+Na]^+$: 745.3193; found 745.3189.

Reaction of 1 with $HGeEt_3$: Formation of $OsH_5(GeEt_3)(P^iPr_3)_2$ (10).

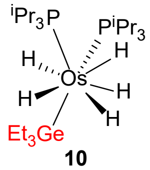

10

A mixture of **1** (50 mg, 0.10 mmol) and Et_3GeH (16.2 µL, 0.10 mmol) in octane (4 mL) was stirred at 50 ºC for 24 h, in an Schlenk-tube attached to a condenser provided with a gas bubbler. The crude reaction was concentrated to dryness under reduced pressure, giving a yellow oil. The NMR spectra showed the presence of **1**, **10**, and **11** in a 20:56:24 molar ratio. NMR data for **10**: ^1H NMR (300.13 MHz, C_6D_6, 298 K): δ 1.85 (m, 6H, PCH), 1.40 (t, $^3J_{H,H}$ = 7.7, 9H, $GeEt_3$), 1,10 (m, 42H, $PCH(CH_3)_2$ + $GeEt_3$), -10.40 (t, $^2J_{H,P}$ = 9.0, 5H, Os-H). $^{31}P\{^1H\}$ NMR (121.50 MHz, C_6D_6, 298 K): δ 44.3 (s). $^{13}C\{^1H\}$-apt NMR (75 MHz, C_6D_6, 298 K): 29.1 (PCH partially overlapped with PCH of **11**), 20.5 (s, $PCCH_3$), 14.3 (s, $SiCH_2CH_3$), 11.0 (s, $SiCH_2CH_3$). $T_{1(min)}$ (ms, OsH_5, 300.13 MHz, toluene-d_8, 228 K): 136 ± 14 (−10.40 ppm). HRMS (electrospray, m/z): calcd. for $C_{24}H_{62}GeOsP_2$ $[M]^+$: 678.3148; found 678.3129.

Preparation of $OsH_4\{\kappa^1\text{-}P,\eta^2\text{-}GeH\text{-}[^iPr_2PCH(Me)CH_2GeEt_2H]\}(P^iPr_3)$ (11).

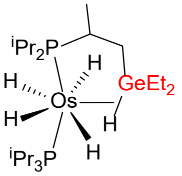

11

A solution of **1** (50 mg, 0.10 mmol) in toluene (4 mL) was treated with Et_3GeH (16.2 µL, 0.10 mmol). The resulting mixture was heated at 90 ºC for 5 h, in a Schlenk-tube attached to a condenser provided with a gas bubbler. The crude reaction was concentrated to dryness under reduced pressure, giving a yellow oil. The ^1H and ^{31}P NMR spectra showed the quantitative formation of **11**. ^1H NMR (300.13 MHz, C_6D_6, 298K): 1.81 (m, 6H, PCH), 1.50-1.30 (m, 11H, Ge*Et₂* + 1H PCC*H₂*Ge), 1.19-1.00 (m, 33H, PC(C*H₃*)₂), 0.84 (ddd, $^2J_{H,H}$ = $^3J_{H,H}$ = 13.4, $^3J_{H,P}$ = 5.6 , 1H, PCC*H₂*Ge), -11.00 (dd, $^2J_{H,P}$ = 14.5, 9.3, 5H, Os-H). $^{31}P\{^1H\}$ NMR (121.50 MHz, C_6D_6, 298 K): δ 69.2 (AB spin system, Δν = 3148 Hz, $J_{A\text{-}B}$ = 138.5 Hz). $^{13}C\{^1H\}$-apt NMR (75 MHz, C_6D_6, 298 K): δ 36.0 (d, $J_{C,P}$ = 26.6, PCH P^iPr_2), 29.9 (dd, $J_{C,P}$ = 28.9, 2.0, PCH P^iPr_2), 29.6 (dd, $J_{C\text{-}P}$ = 29.3, 1.5, PCH P^iPr_3), 28.5 (dd, $J_{C,P}$ = 26.1, 2.0, P*C*HCH₂Ge), 26.2 (dd, $J_{C,P}$ = 30.1, 1.7, PCH*C*H₂Ge), 21.8, 20.8 (both s, PC*C*H₃), 20.6 (s, PC*C*H₃ P^iPr_3), 20.3 (d, $^2J_{C,P}$ = 2.1, PC*C*H₃), 19.2 (s, PC*C*H₃), 18.3 (d, $^2J_{C,P}$ = 1.2, PC*C*H₃), 18.2, 17.6 (both s, Ge*C*H₂CH₃), 11.4, 11.1 (both s, GeCH₂*C*H₃). $T_{1(min)}$ (ms, OsH_5, 300.13 MHz, toluene-d_8, 208 K): 150 ± 15 (−11.00 ppm). IR (cm^{-1}): ν(Os-H) 1996, 1901 (w). HRMS (electrospray, m/z): calcd. for $C_{22}H_{55}GeOsP_2$ $[M\text{-}H]^+$: 647.2601; found 647.2621.

NMR Spectroscopy Study of the transformation of 3 into 5. In the glovebox, an NMR tube was charged with a solution of **3** (15 mg, 0.02 mmol) in toluene (0.60 mL), and a capillary tube filled with a solution of the internal standard (PPh_3) in toluene was placed in the NMR tube. The tube was immediately introduced into an NMR probe preheated at the desired temperature, and the reaction was monitored by $^{31}P\{^1H\}$ NMR (a delay of 32 s was used) at different intervals of time. Rate constants were obtained by plotting Equation 1 .

$$ln\frac{[\mathbf{3}]}{[\mathbf{3}]_0} = -kt \qquad (1)$$

Kinetic Plots

363 K

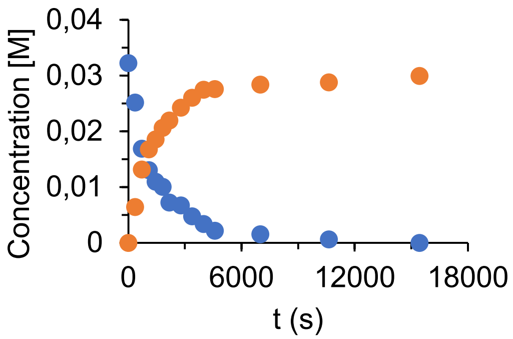

368 K

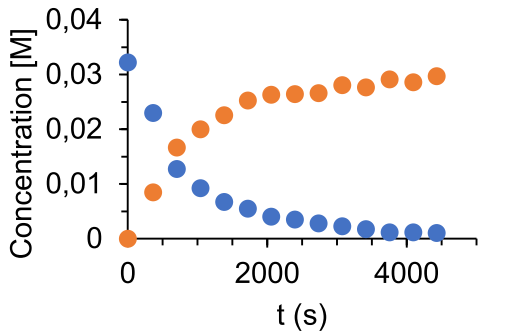

373 K

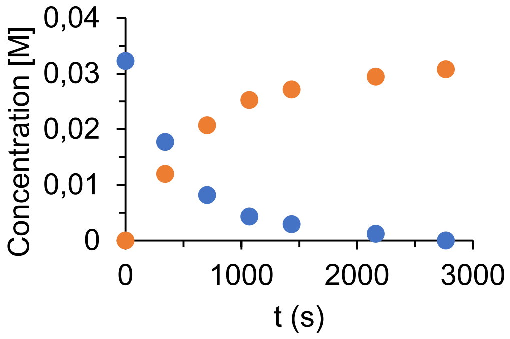

378 K

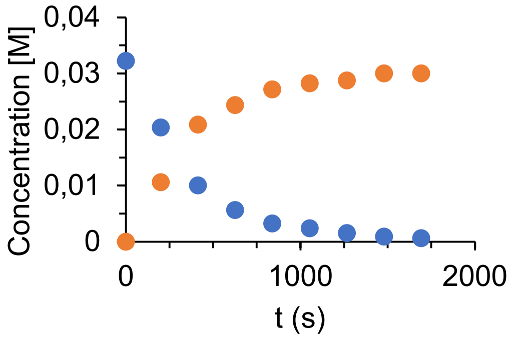

388 K

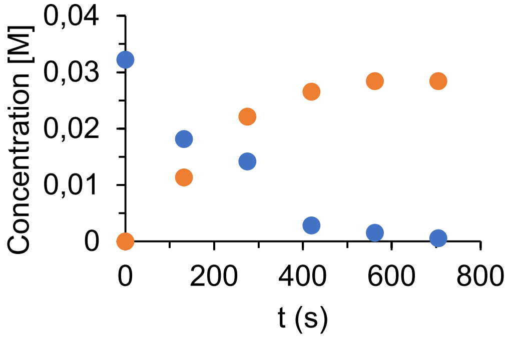

Figure S1. Concentration *versus* time plots for the transformation of **3** (blue circles) into **5** (orange circles) at different temperatures.

Structural Analysis of Complexes 3 and 5. X-ray data were collected for the complex on a Bruker APEX CCD (**3**) and DUO CCD (**5**) diffractometers equipped with a normal or fine focus, and 2.4 kW sealed tube source (Mo radiation, λ = 0.71073 Å). Data were collected over the complete sphere covering 0.3° in ω. Data were corrected for absorption by using a multiscan method applied with the SADABS program.[2] The structures were solved by Patterson or direct methods and refined by full-matrix least squares on F^2 with SHELXL2016,[3] including isotropic and subsequently anisotropic displacement parameters. The hydrogen atoms were observed in the last Fourier Maps or calculated, and refined freely or using a restricted riding model. The hydride ligands were observed in the difference Fourier maps and refined with a restrained distance to osmium atoms ($d_{Os\text{-}H}$ = 1.59 Å).

Crystal data for **3** (CCDC 2088334): $C_{36}H_{62}OsP_2Si$, M_W 775.08, colourless, irregular block (0.151 x 0.135 x 0.126 mm^3), monoclinic, space group P21/n, *a*: 8.9835(5) Å, *b*: 21.1769(11) Å, *c*: 19.0540(10) Å, β: 95.8480(10)°, V = 3606.0(3) $Å^3$, Z = 4, Z' = 1, D_{calc}: 1.428 g cm^{-3}, F(000): 1592, T = 100(2) K, μ 3.681 mm^{-1}. 62232 measured reflections (2θ: 3-57°, ω scans 0.3°), 8965 unique (R_{int} = 0.0528); min./max. transm. Factors 0.727/0.862. Final agreement factors were R^1 = 0.0296 (6929 observed reflections, I > 2σ(I)) and wR^2 = 0.0635; data/restraints/parameters 8965/5/ 388; GoF = 1.012. Largest peak and hole 1.856 (close to osmiun atoms) and -1.573 e/ $Å^3$.

Crystal data for **5** (CCDC 2088335): $C_{30}H_{56}OsP_2Si$, M_W 696.97, colourless, irregular block (0.182 x 0.145 x 0.079 mm^3), monoclinic, space group P21/n, *a*: 17.4758(15) Å, *b*: 9.7446(8) Å, *c*: 20.5035(18) Å, β: 115.0710(10)°, V = 3162.7(5) $Å^3$, Z = 4, Z' = 1, D_{calc}: 1.464 g cm^{-3}, F(000): 1424, T = 120(2) K, μ 4.188 mm^{-1}. 103113 measured reflections (2θ: 3-57°, ω scans 0.3°), 8820 unique (R_{int} = 0.0440); min./max. transm. Factors 0.742/0.862. Final agreement factors were R^1 = 0.0205 (7460 observed reflections, I > 2 σ(I)) and wR^2 = 0.0475; data/restraints/parameters 8820/5/ 333; GoF = 1.013. Largest peak and hole 1.465 (close to osmiun atoms) and -0.452 e/ $Å^3$.

Computational details: Geometry optimizations of complexes **3** and **5** were performed at the DFT level using the B3LYP functional[4] supplemented with the Grimme's dispersion correction D3[5] including an ultrafine integration grid, as implemented in Gaussian09.[6] Os atom was described by means of an effective core potential SDD for the inner electron[7] and its associated double-ζ basis set for the outer ones, complemented with a set of f-polarization functions.[8] The 6-31G** basis set was used for the H, C, Si, and P atoms.[9] All geometries were fully optimized in vacuum and characterized as minimun. The topology of the electron density was conducted using the AIMAll program package.[10]

The Cartesian coordinates for the computed structures can be found in the supplemental file xyz. The file may be opened as a text file to read the coordinates, or opened directly by a molecular modeling program such as Mercury (version 3.3 or later, http://www.ccdc.cam.ac.uk/pages/Home.aspx) for visualization and analysis.

References

(1) M. Aracama, M. A. Esteruelas, F. J. Lahoz, J. A. López, U. Meyer, L. A. Oro, H. Werner, *Inorg.Chem.*, **1991**, *30*, 288−293.

(2) R. H.Blessing, *Acta Crystallogr.* **1995**, *A51*, 33. SADABS: Area-detector absorption correction; Bruker-AXS, Madison, WI, 1996.

(3) SHELXL-2016/6. G. M. Sheldrick, *Acta Cryst***.** **2008**, *A64*, 112–122.

(4) a) C. Lee, W. Yang, R. G. Parr, *Phys. Rev. B* **1988**, *37*, 785–789; b) A. D. Becke, *J. Chem. Phys.* **1993**, *98*, 5648–5652; c) P. J. Stephens, F. J. Devlin, C. F. Chabalowski, M. J. Frisch, *J. Phys. Chem.* **1994**, *98*, 11623–11627.

(5) S. Grimme, J. Antony, S. Ehrlich, H. Krieg, *J. Chem. Phys*., **2010**, *132*, 154104–154123.

(6) Gaussian 09, Revision D.01, M. J. Frisch, G.W. Trucks, H. B. Schlegel, G. E. Scuseria, M.A. Robb, J .R. Cheeseman, G. Scalmani, V. Barone, B. Mennucci, G. A. Petersson, H. Nakatsuji, M. Caricato, X. Li, H. P. Hratchian, A. F. Izmaylov, J. Bloino, G. Zheng, J. L. Sonnenberg, M. Hada, M. Ehara, K. Toyota, R. Fukuda, J. Hasegawa, M. Ishida, T. Nakajima, Y. Honda, O. Kitao, H. Nakai, T. Vreven, J. A. Montgomery, J. E. Peralta, F. Ogliaro, M. Bearpark, J. J. Heyd, E. Brothers, K. N. Kudin, V. N. Staroverov, T. Keith, R. Kobayashi, J. Normand, K. Raghavachari, P. G. Rendell, J. C Burant, S. S. Iyengar, J. Tomasi, M. Cossi, N. Rega, J. M. Millam, M. Klene, J. E. Knox, J. B. Cross, V. Bakken, C. Adamo, J. Jaramillo, R. Gomperts, R. E. Stratmann, O. Yazyev, A. J. Austin, R. Cammi, C. Pomelli, J. W. Ochterski, R. L. Martin, K. Morokuma, V. G. Zakrzewski, G. A. Voth, P. Salvador, J. J. Dannenberg, S. Dapprich, A. D Daniels, O. Farkas, J. B. Foresman, J. V. Ortiz, J. Cioslowski, D. J. Fox, Gaussian, Inc., Wallingford CT, 2013.

(7) D. Andrae, U. M. Haeussermann, M. Dolg, H. Stoll, H. Preuss, *Theor. Chim. Acta* **1990**, *77*, 123–141.

(8) A. W. Ehlers, M. Bohme, S. Dapprich, A. Gobbi, A. Hollwarth, V. Jonas, K. F. Kohler, R. Stegmann, A. Veldkamp, G. Frenking, *Chem. Phys. Lett.* **1993**, *208*, 111–114.

(9) a) W. J. Hehre, R. Ditchfield, J. A. Pople, *J. Chem. Phys.* **1972**, *56*, 2257–2261. b) M. M. Francl, W. J. Pietro, W. J. Hehre, J. S. Binkley, M. S. Gordon, D. J. DeFrees, J. A. Pople, *J. Chem. Phys.* **1982**, *77*, 3654–3665.

(10) T. A. Keith, AIMAll, version 15.09.27; TK Gristmill Software: Overland Park, KS, 2015.

(a) (b)

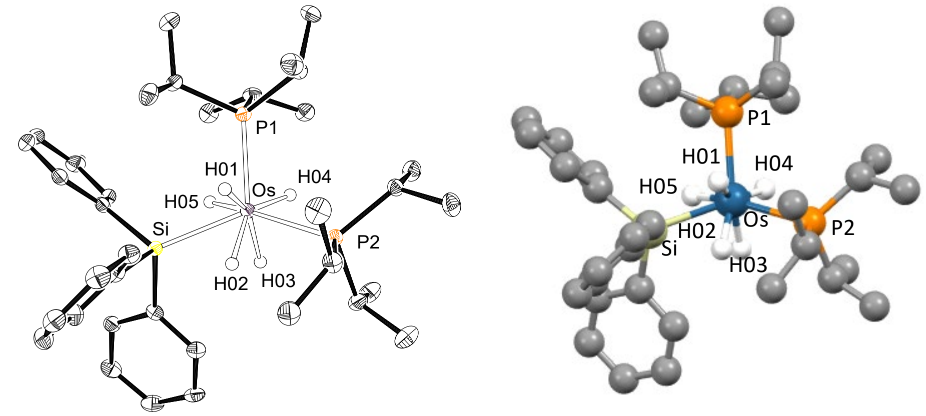

Figure S2. (a) X-ray structure of complex **3** with 50% probability ellipsoids. (b) DFT-optimized structure of **3**. Hydrogen atoms (except hydrides) are omitted for clarity. Selected bond lengths (Å) and angles (deg) for the X-ray and optimized (in square brackets) structures: Os−H(01) = 1.581(10) [1.662], Os−H(02) = 1.582(10) [1.635], Os−H(03) = 1.593(10) [1.629], Os−H(04) = 1.593(10) [1.657], Os−H(05) = 1.582(10) [1.638], Os−(P1) = 2.3996(8) [2.448], Os−P(2) = 2.3965(8) [2.442], Os−Si = 2.4312(9) [2.458], Si−H(02) = [2.174], Si−H(05) = [2.123]; P1−Os−P2 = 110.92(3) [109.84], Si−Os−P1 = 109.70(3) [106.95], Si−Os−P2 = 133.72(3) [134.04].

(a) (b)

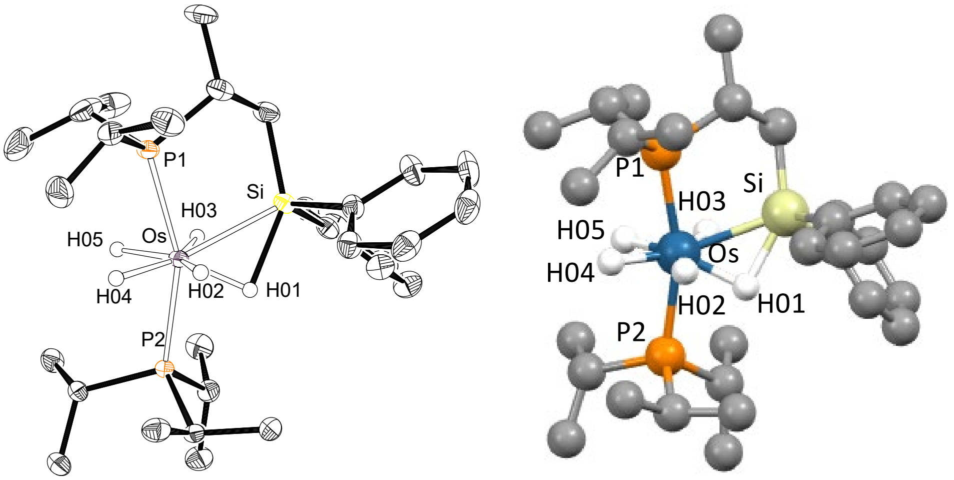

Figure S3. (a) X-ray structure of complex **5** with 50% probability ellipsoids. (b) DFT-optimized structure of **3**. Hydrogen atoms (except hydrides) are omitted for clarity. Selected bond lengths (Å) and angles (deg) for the X-ray and optimized (in square brackets) structures: Os−H(01) = 1.584(9) [1.666], Os−H(02) = 1.577(9) [1.667], Os−H(03) = 1.580(9) [1.674], Os−H(04) = 1.592(9) [1.648], Os−H(05) = 1.590(9) [1.641], Os−(P1) = 2.3512(6) [2.376], Os−P(2) = 2.3449(6) [2.377], Os−Si = 2.4645(6) [2.512], Si−H(01) = 1.91(2) [1.847]; P1−Os−P2 = 157.56(2) [164.01], Si−Os−P1 = 80.98(2) [80.29], Si−Os−P2 = 121.302(19) [115.56].

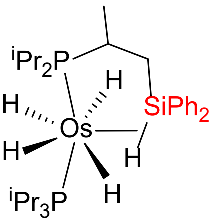

5

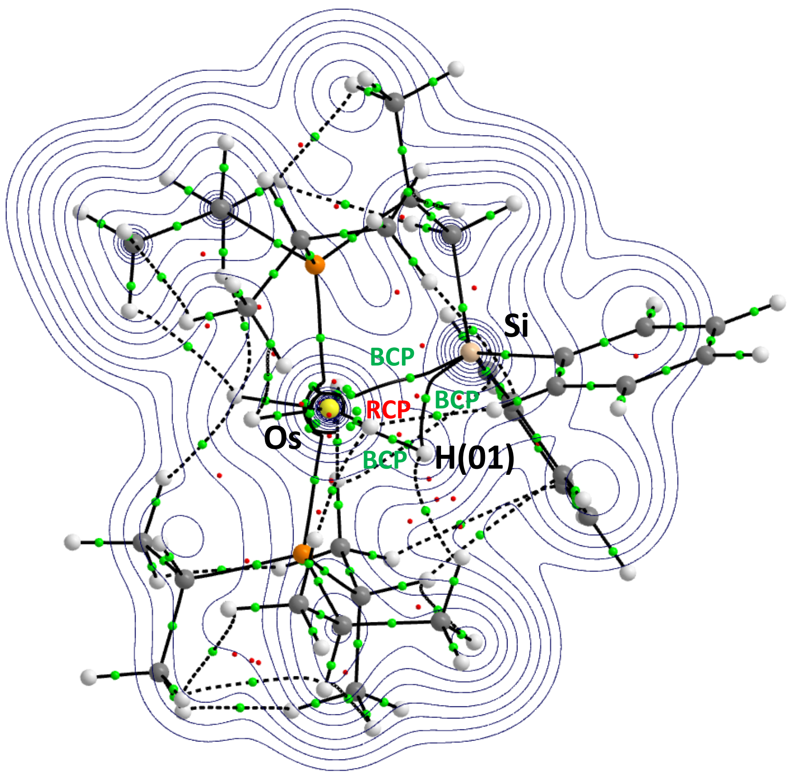

Figure S4. Contour line diagram $\nabla^2\rho(r)$ for complex **5** in the Os−H01−Si plane. The solid lines connecting the atomic nuclei are the bond paths while the small green and red spheres indicate the corresponding BCPs and RCPs ring critical points, respectively.

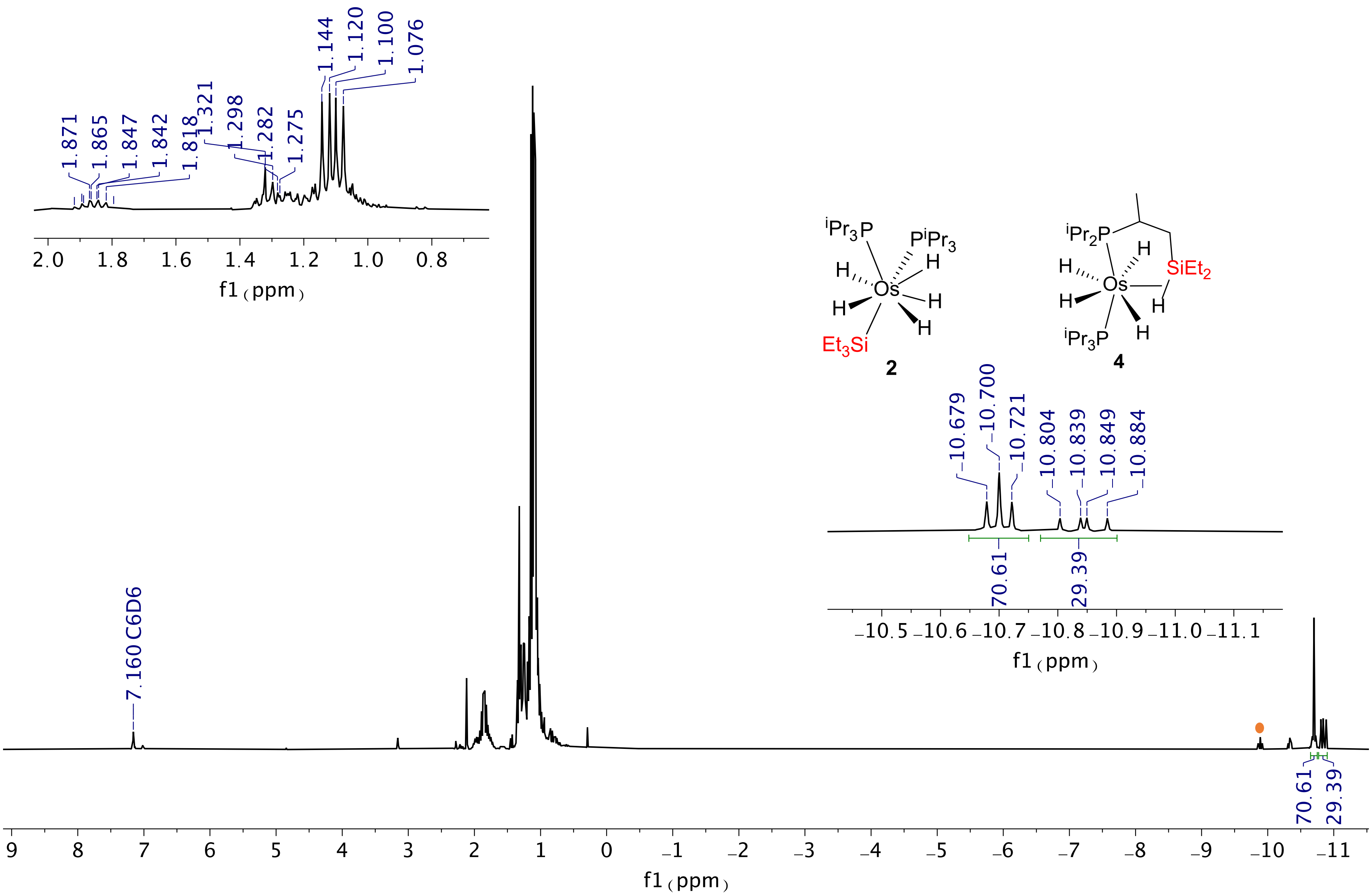

Figure S5. ^{1}H NMR (300.13 MHz, C_6D_6, 298 K) spectrum of the reaction of **1** with $HSiEt_3$ (after heating in n-octane at 65 ºC for 4 h): Formation of **2** and **4** in a 70:30 molar ratio. ● $OsH_6(P^iPr_3)_2$ (**1**).

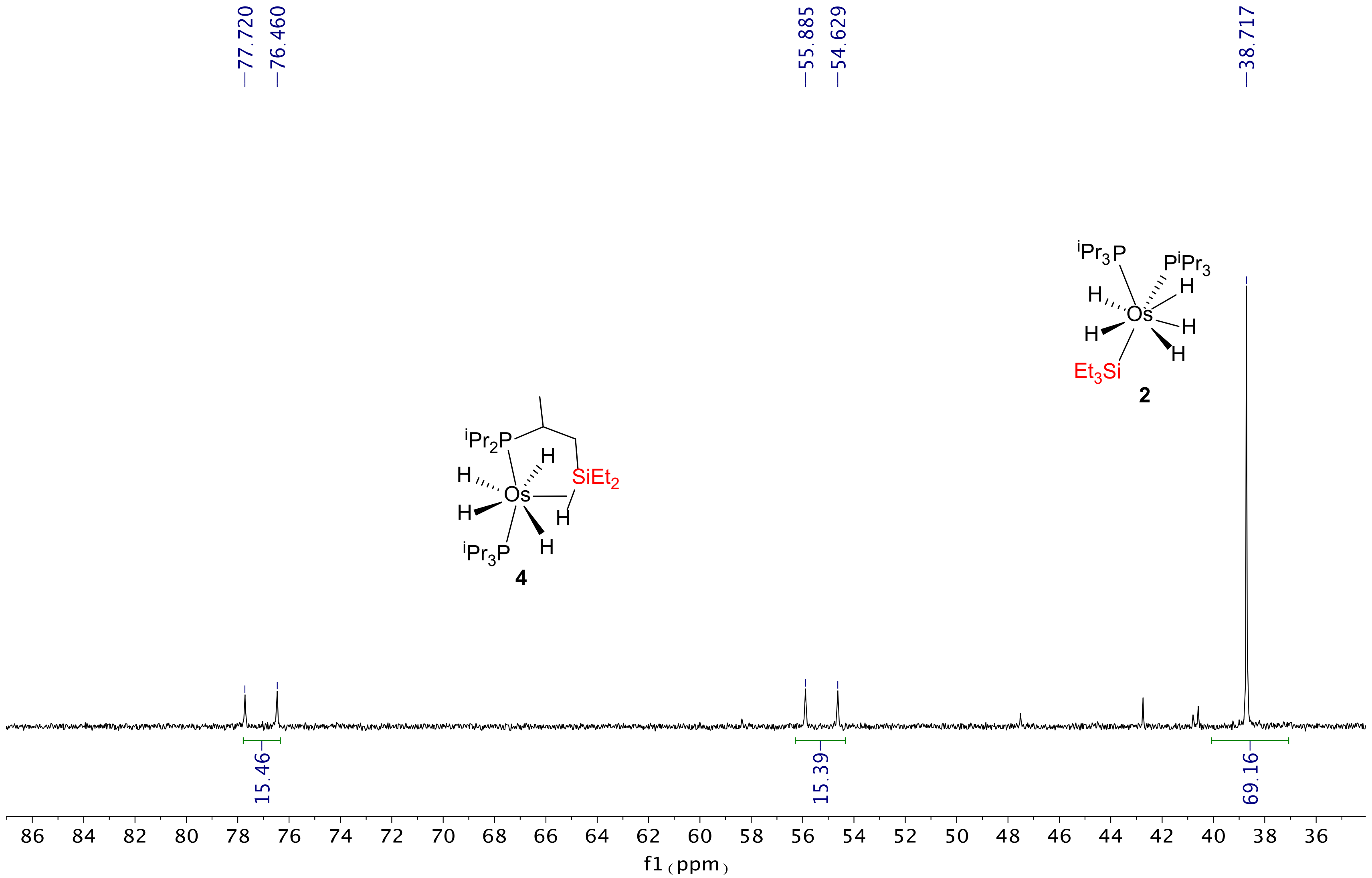

Figure S6. $^{31}P\{^{1}H\}$ NMR (121.50 MHz, C_6D_6, 298 K) spectrum of the reaction of **1** with $HSiEt_3$ (after heating in n-octane at 65 ºC for 4 h): Formation of **2** and **4** in a 70:30 molar ratio.

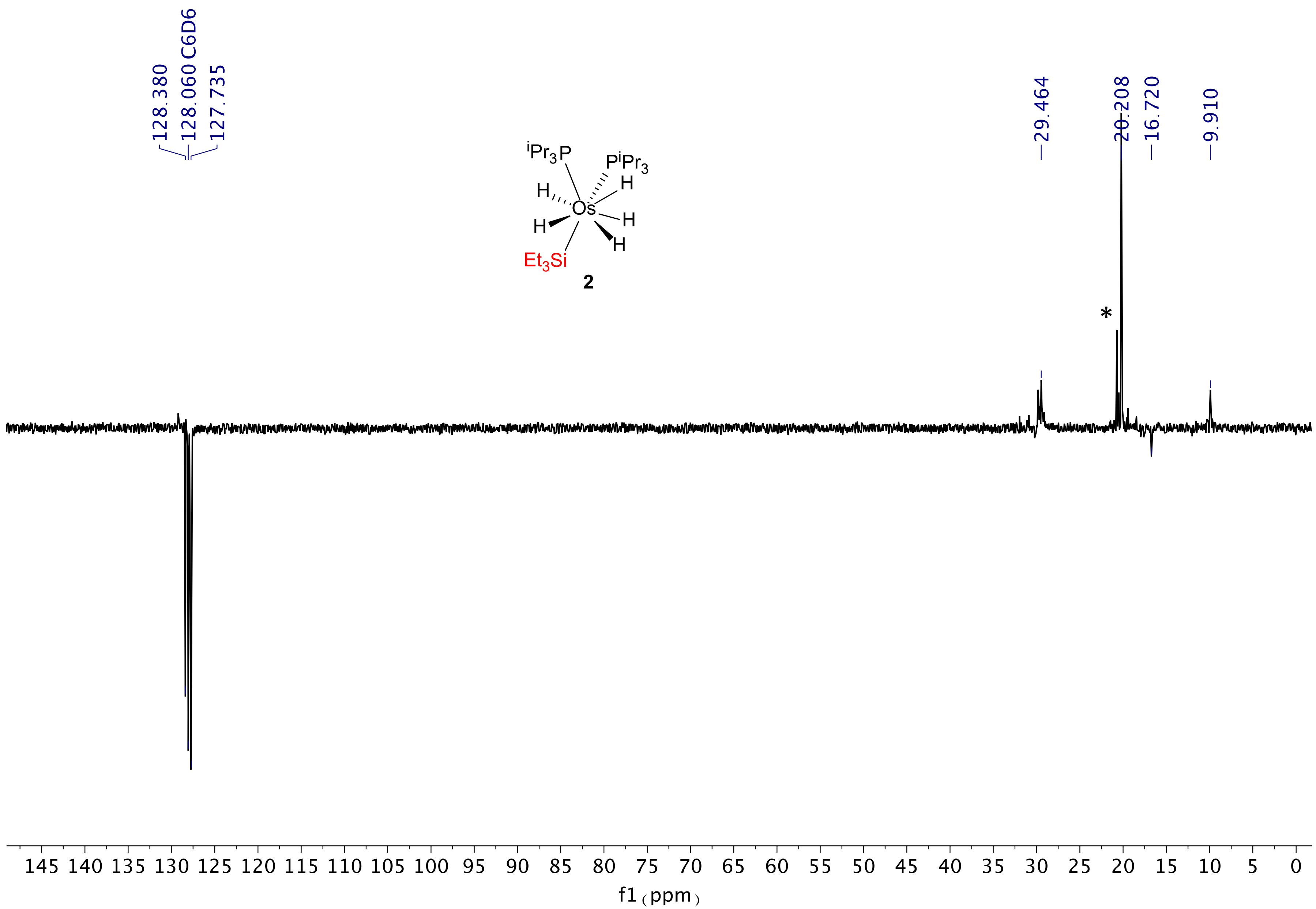

Figure S7. $^{13}C\{^1H\}$-apt NMR (75 MHz, C_6D_6, 298 K) spectrum of the reaction of **1** with $HSiEt_3$ (after heating in n-octane at 65 ºC for 4 h): Formation of **2** and **4** in a 70:30 molar ratio. * Complex **4**.

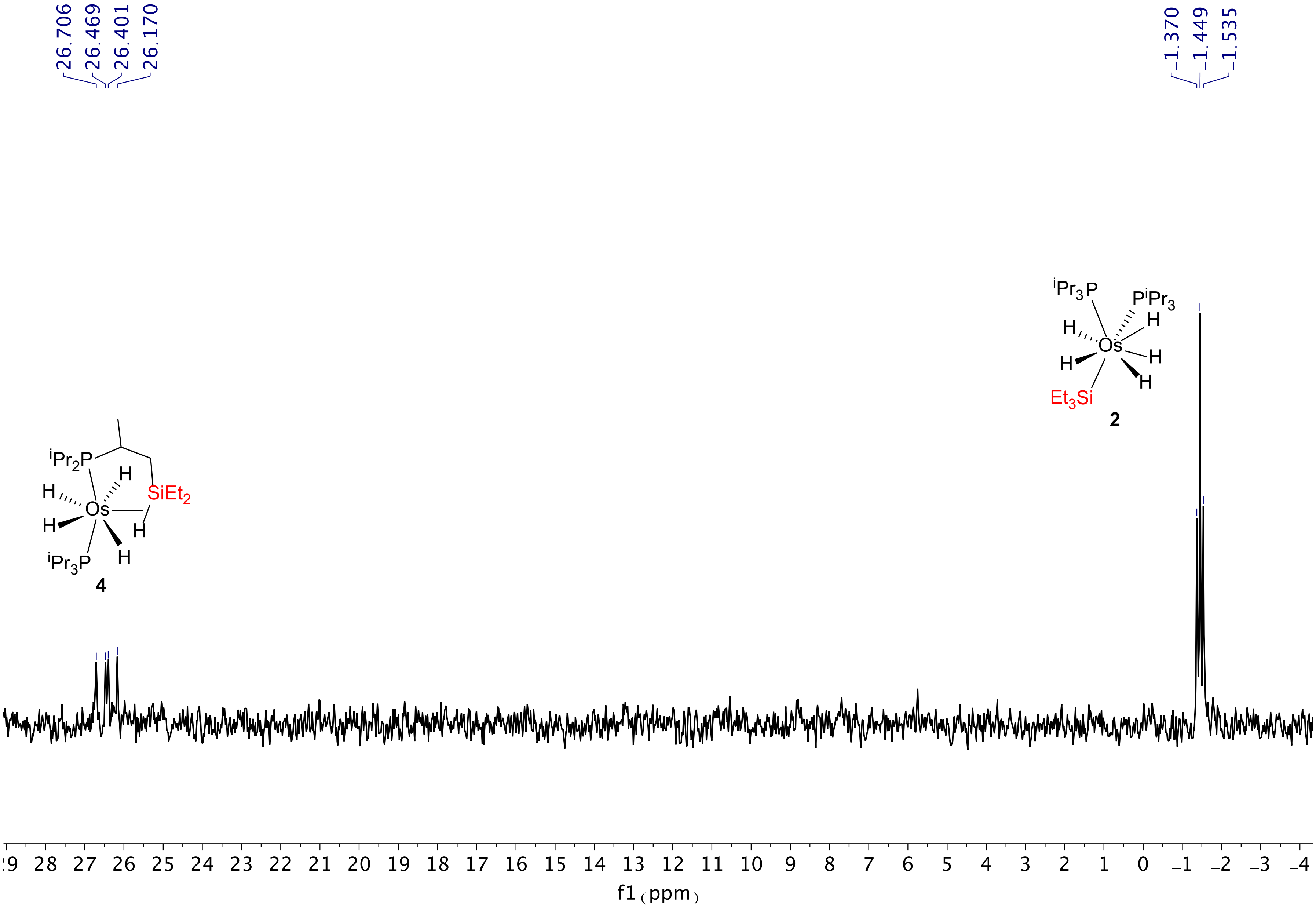

Figure S8. $^{29}Si\{^{1}H\}$ NMR (59.63 MHz, C_6D_6, 298 K) spectrum of spectrum of the reaction of **1** with $HSiEt_3$ (after heating in n-octane at 65 ºC for 4 h): Formation of **2** and **4** in a 70:30 molar ratio.

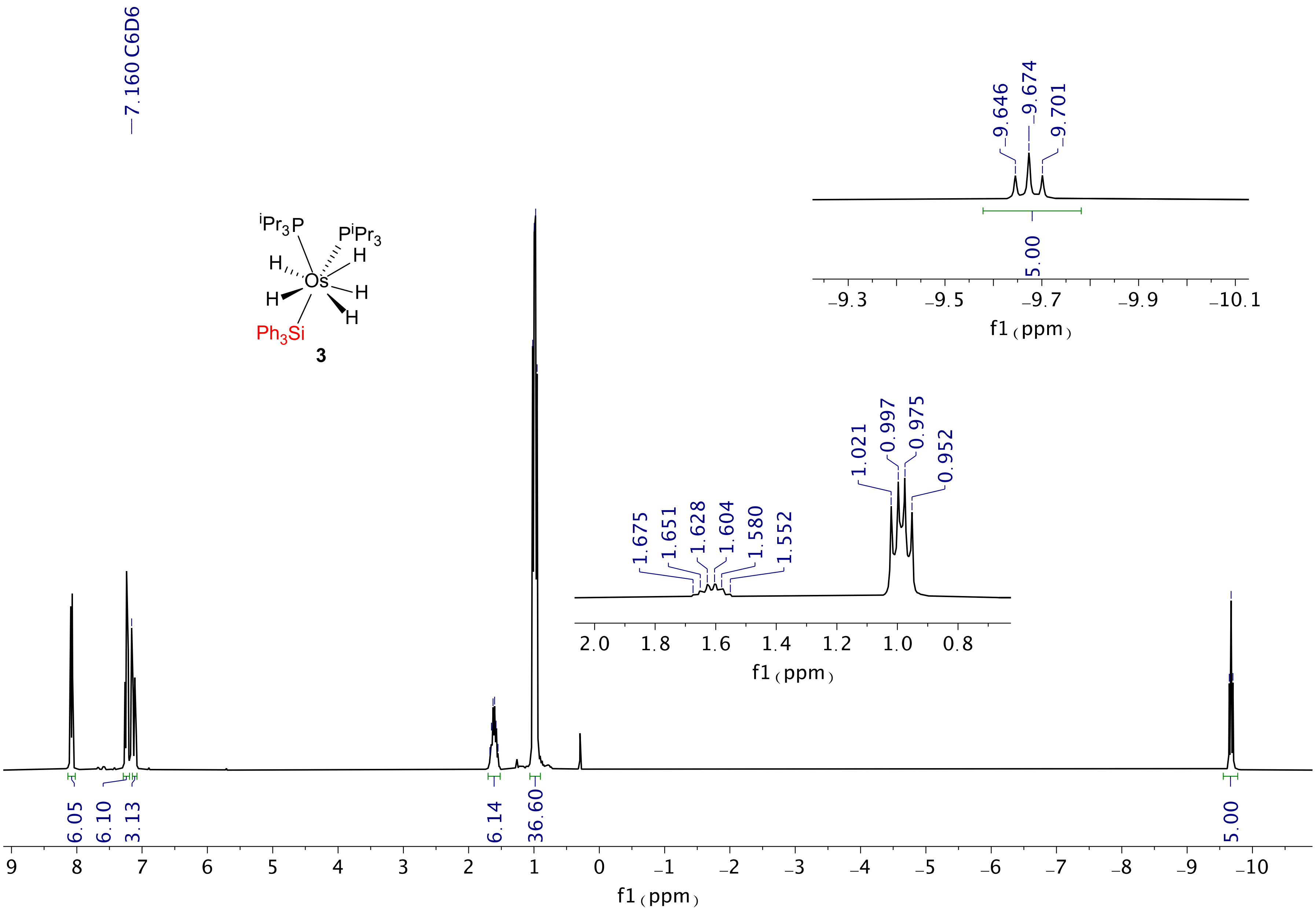

Figure S9. ^{1}H NMR (300.13 MHz, C_6D_6, 298 K) spectrum of complex **3**.

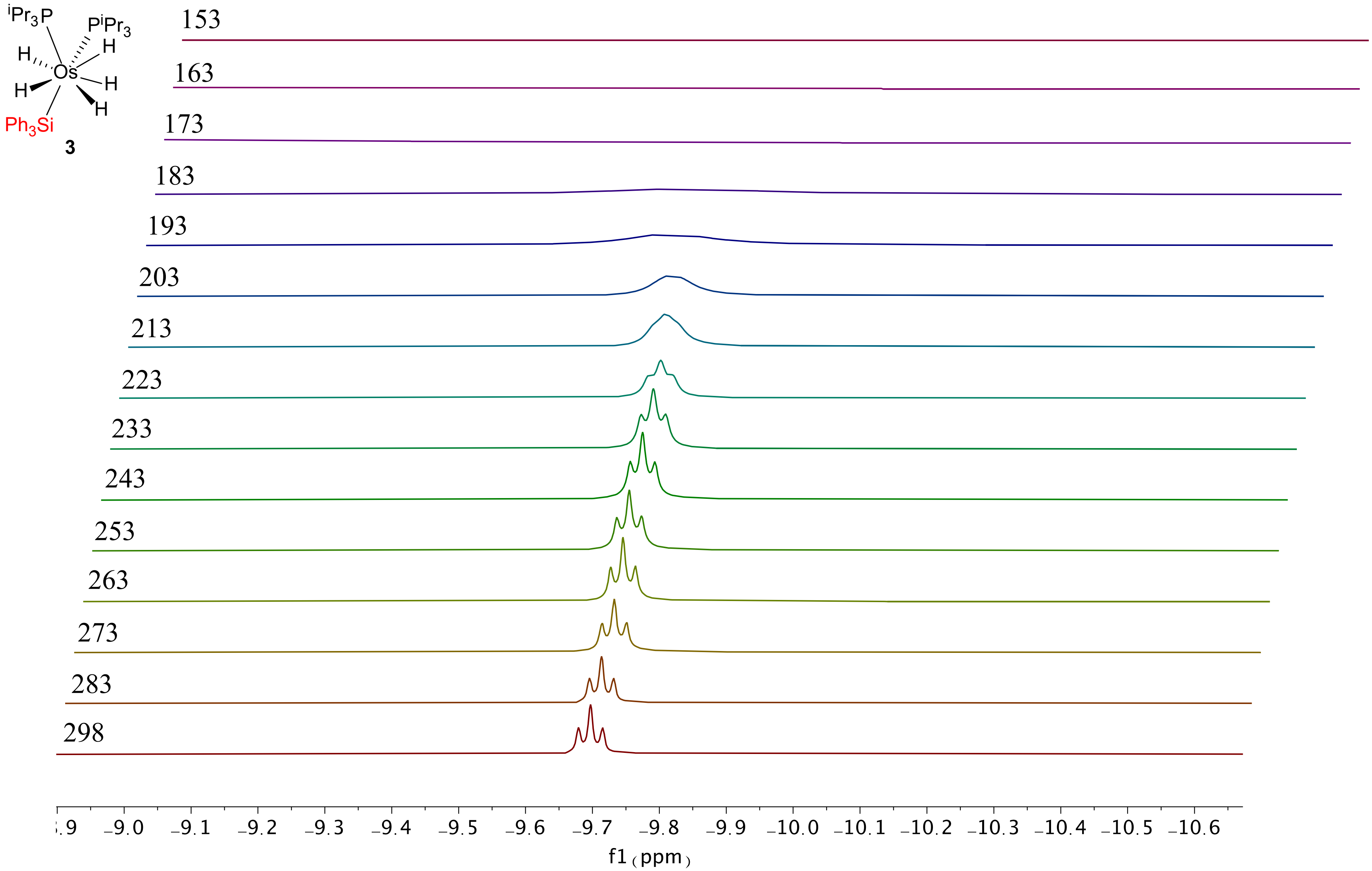

Figure S10. High-field region of the ^1H NMR (500.12 MHz, Methylcyclohexane-d_{14}) spectrum of complex **3** between 298 and 153 K.

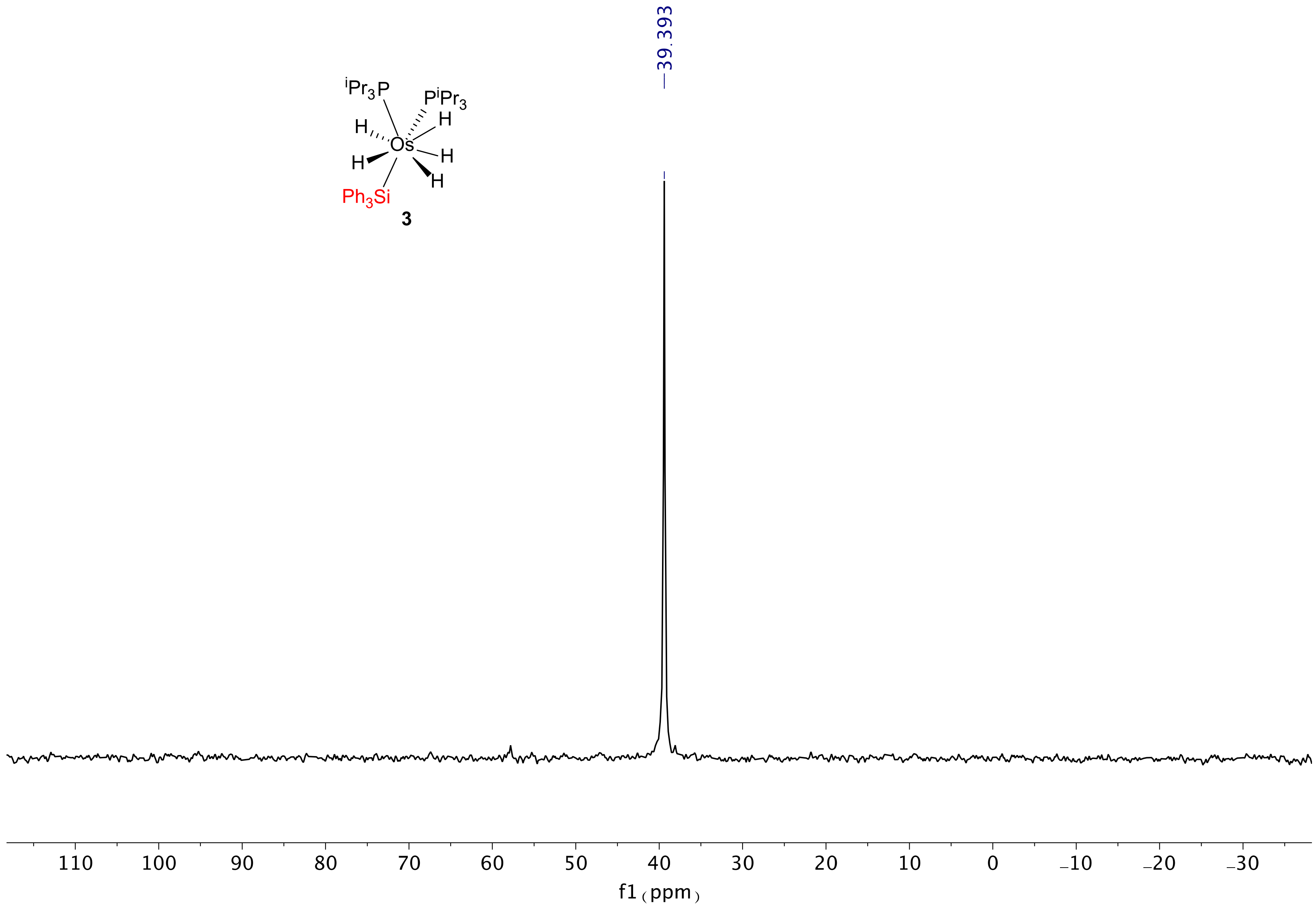

Figure S11. $^{31}P\{^1H\}$ NMR (121.50 MHz, C_6D_6, 298 K) spectrum of **3**.

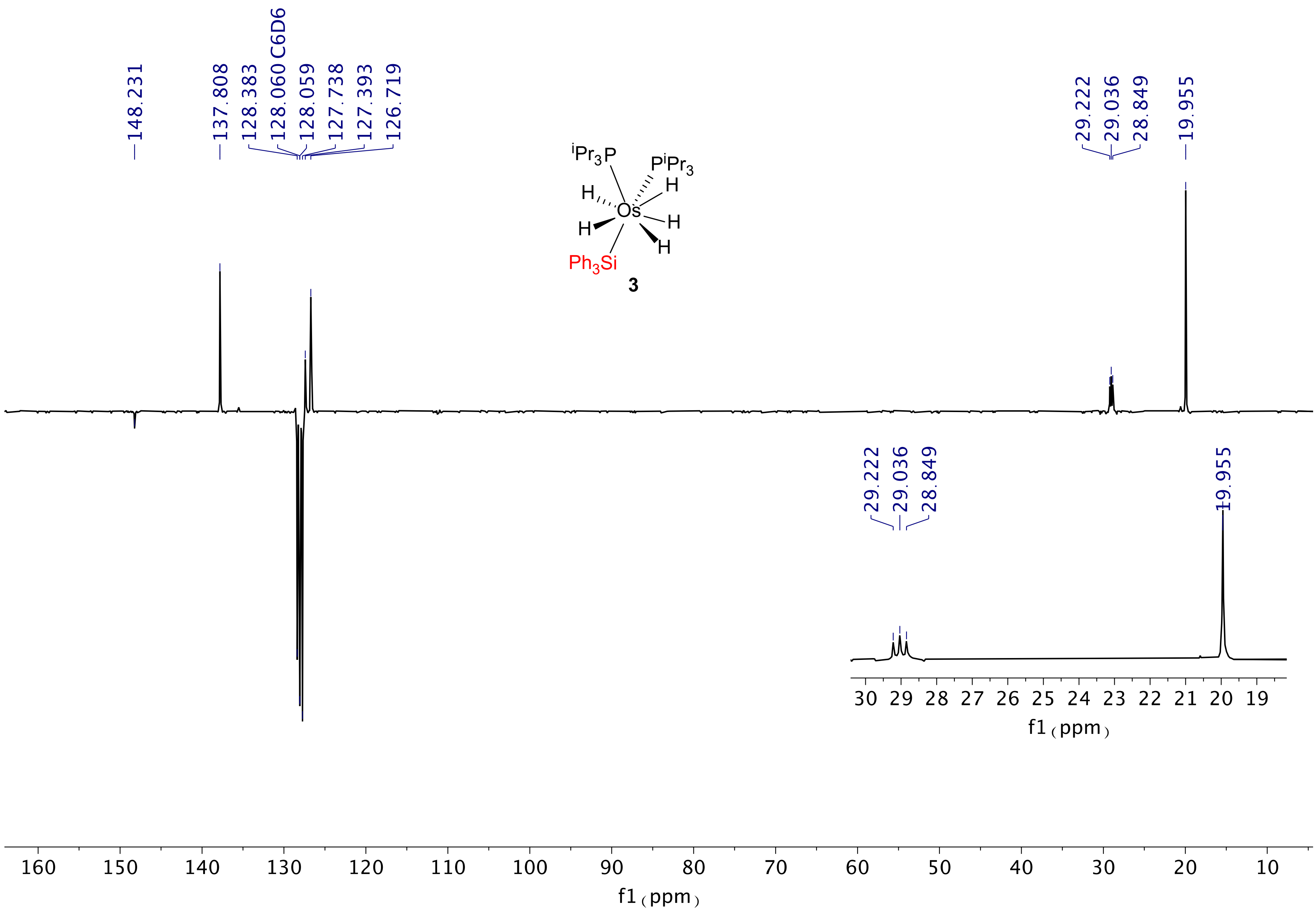

Figure S12. $^{13}C\{^{1}H\}$-apt NMR (75 MHz, C_6D_6, 298 K) spectrum of **3**.

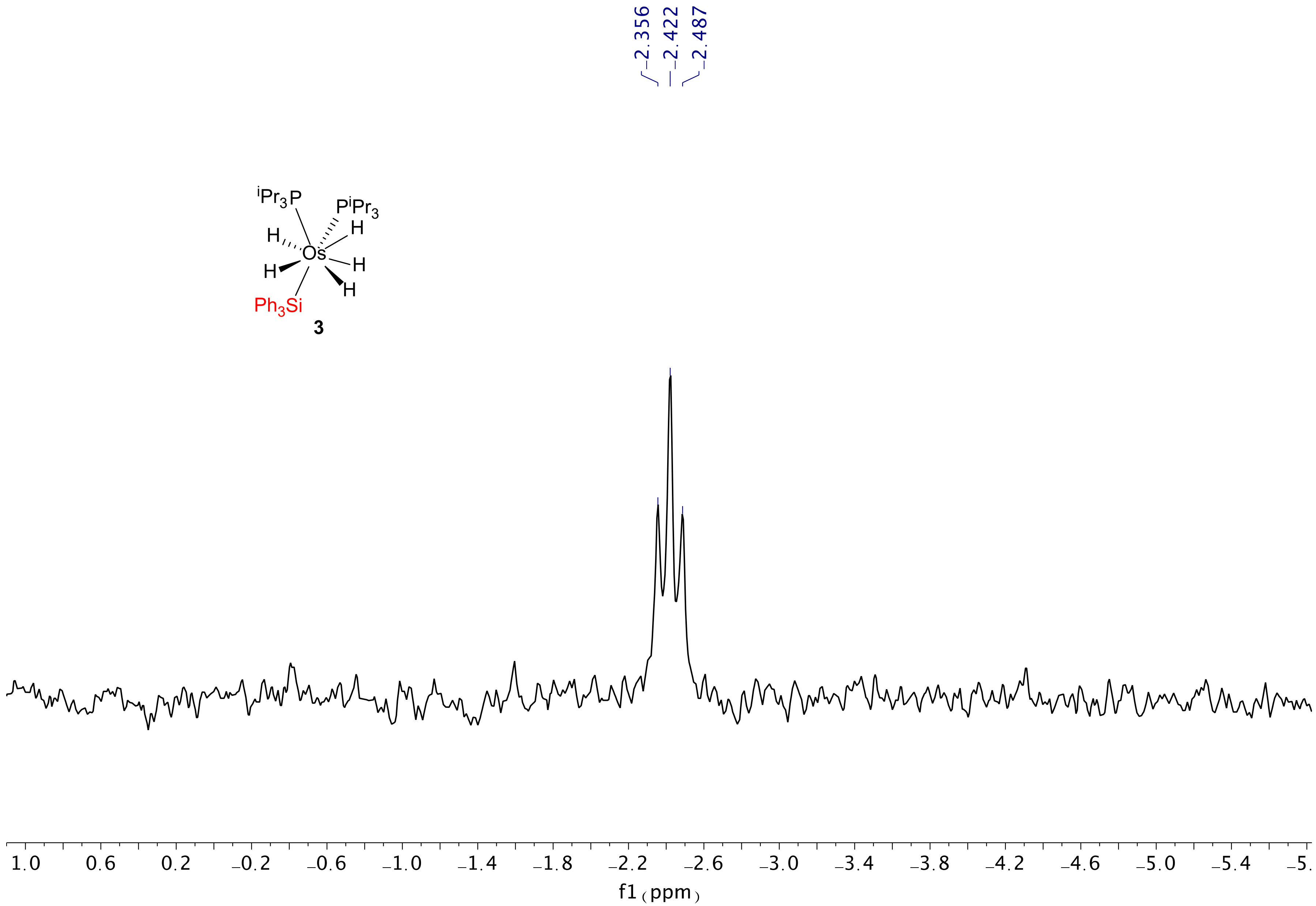

Figure S13. $^{29}Si\{^{1}H\}$ NMR (59.63 MHz, C_6D_6, 298 K) spectrum of **3**.

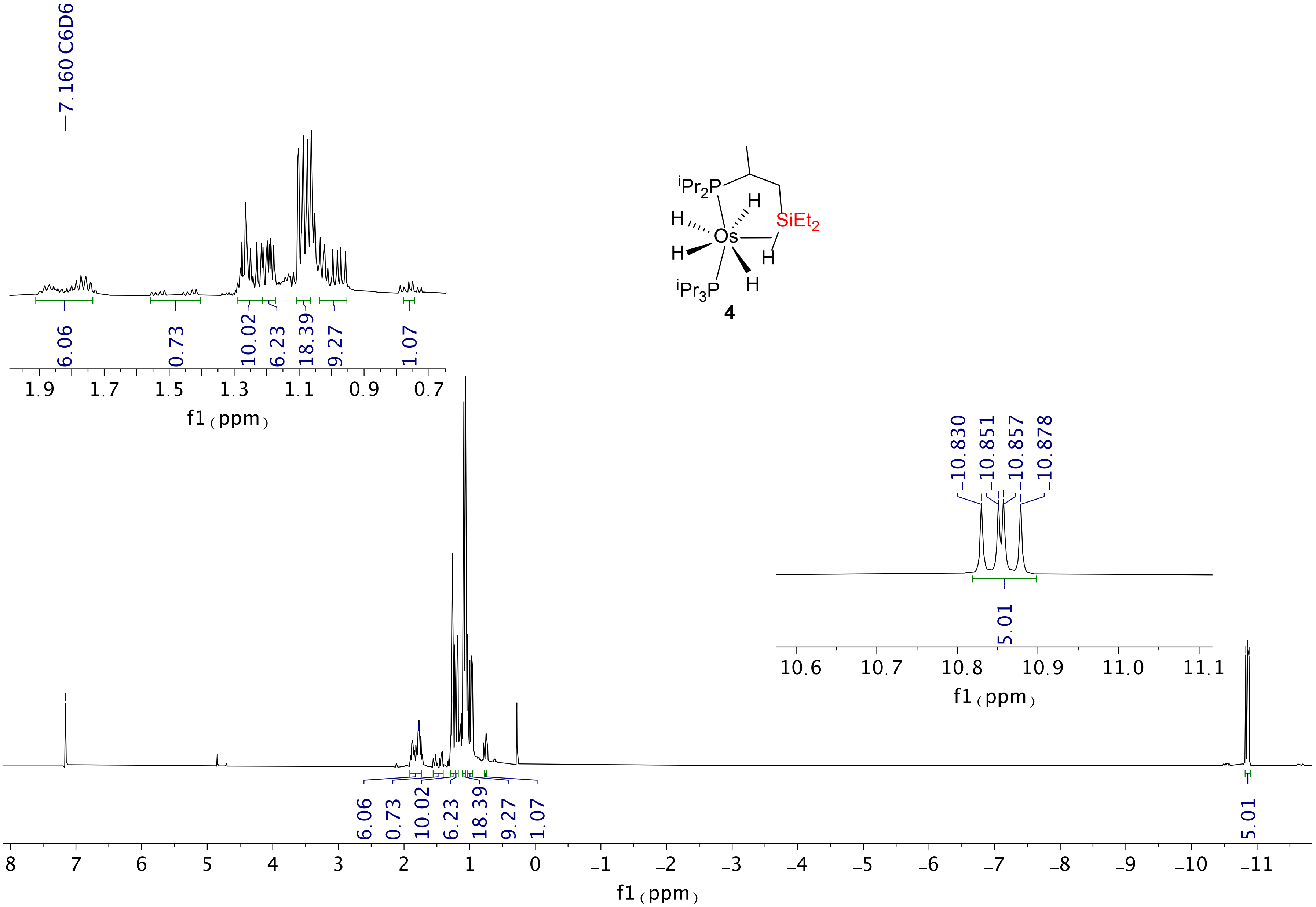

Figure S14. ^1H NMR (500.12 MHz, C_6D_6, 298 K) spectrum of **4**.

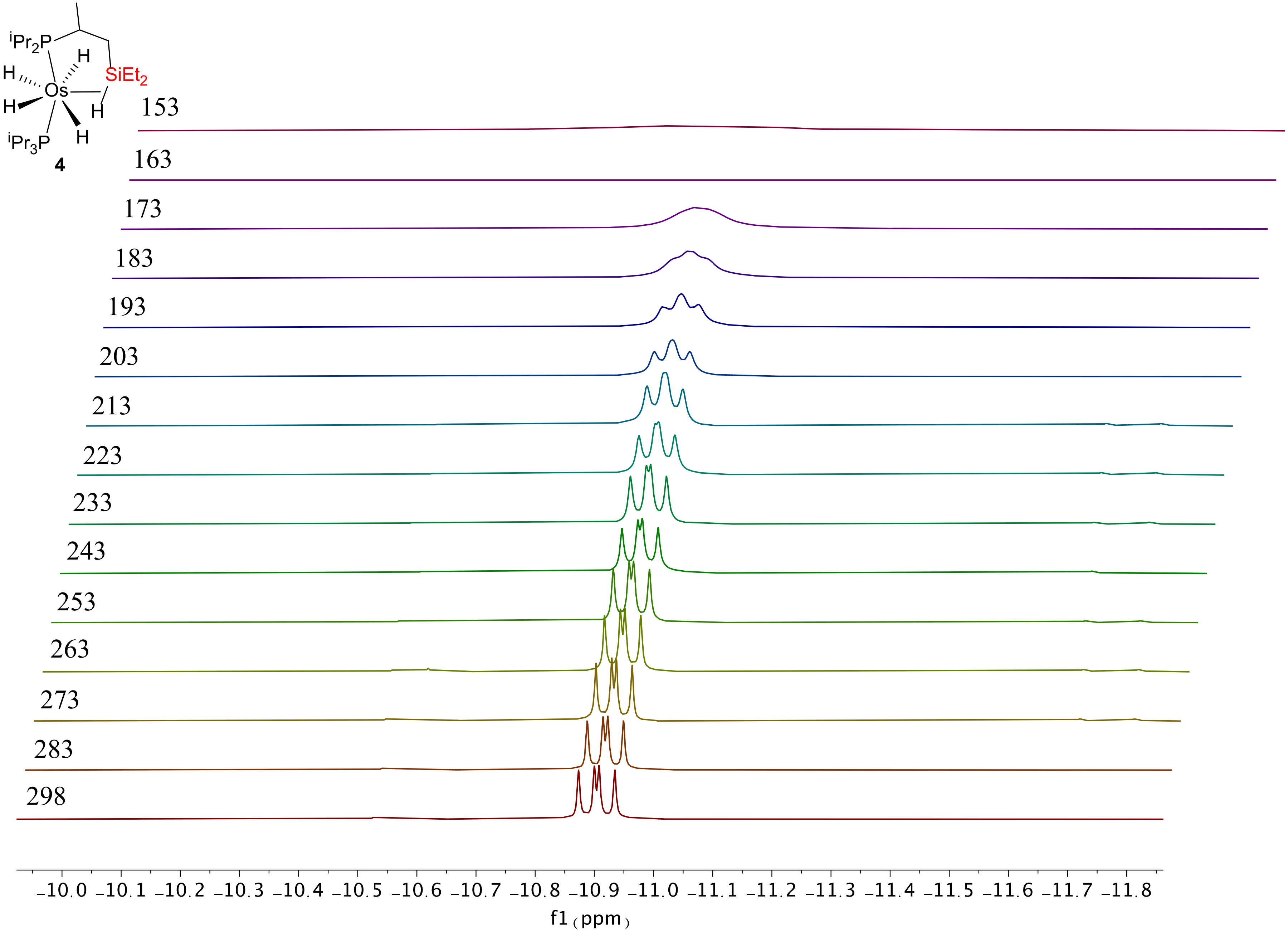

FFigure S15. High-field region of the ^1H NMR (400.13 MHz, Methylcyclohexane-d_{14}) spectrum of **4** between 298 and 153 K.

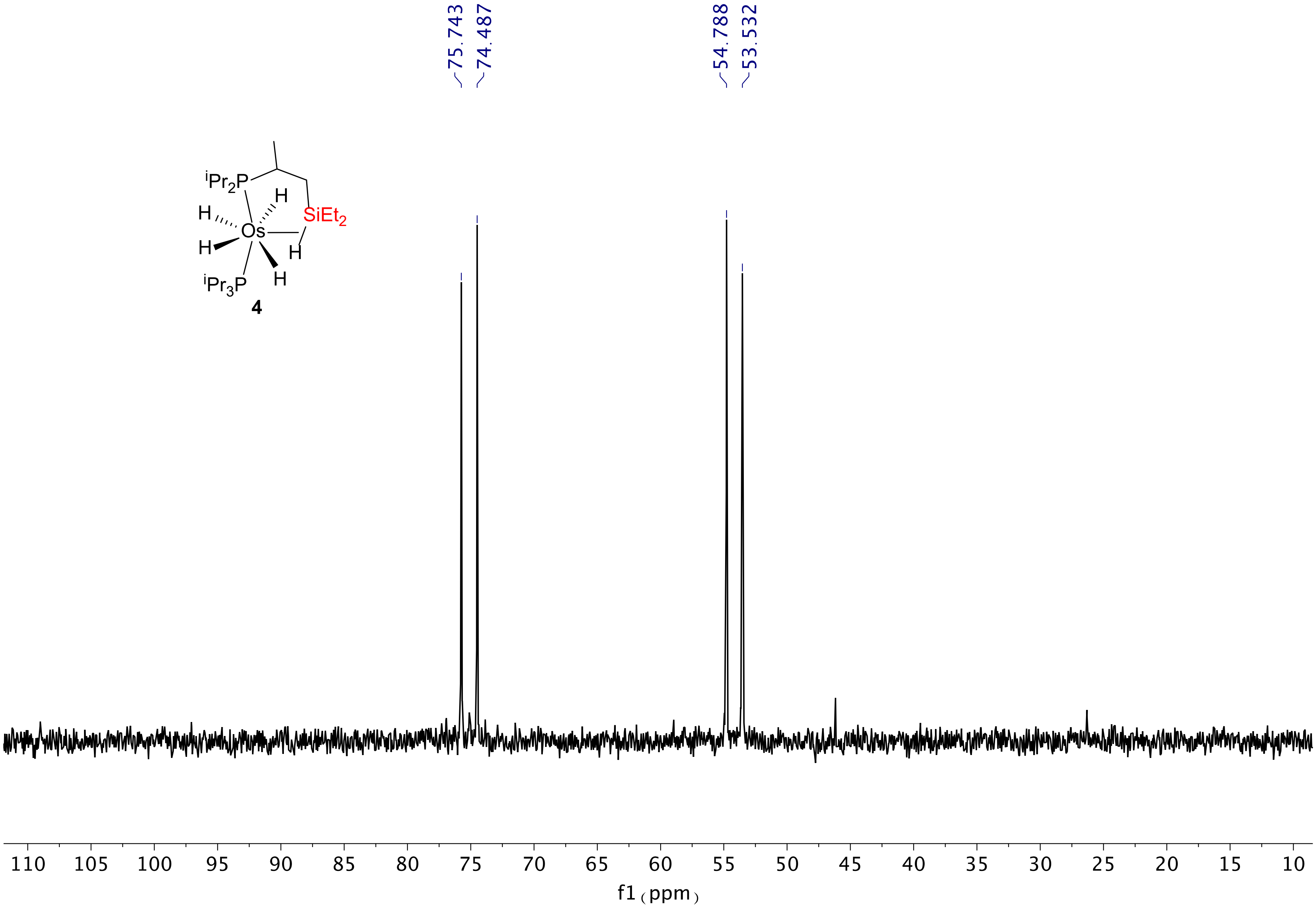

Figure S16. $^{31}P\{^{1}H\}$ NMR (121.50 MHz, C_6D_6, 298 K) spectrum of **4**.

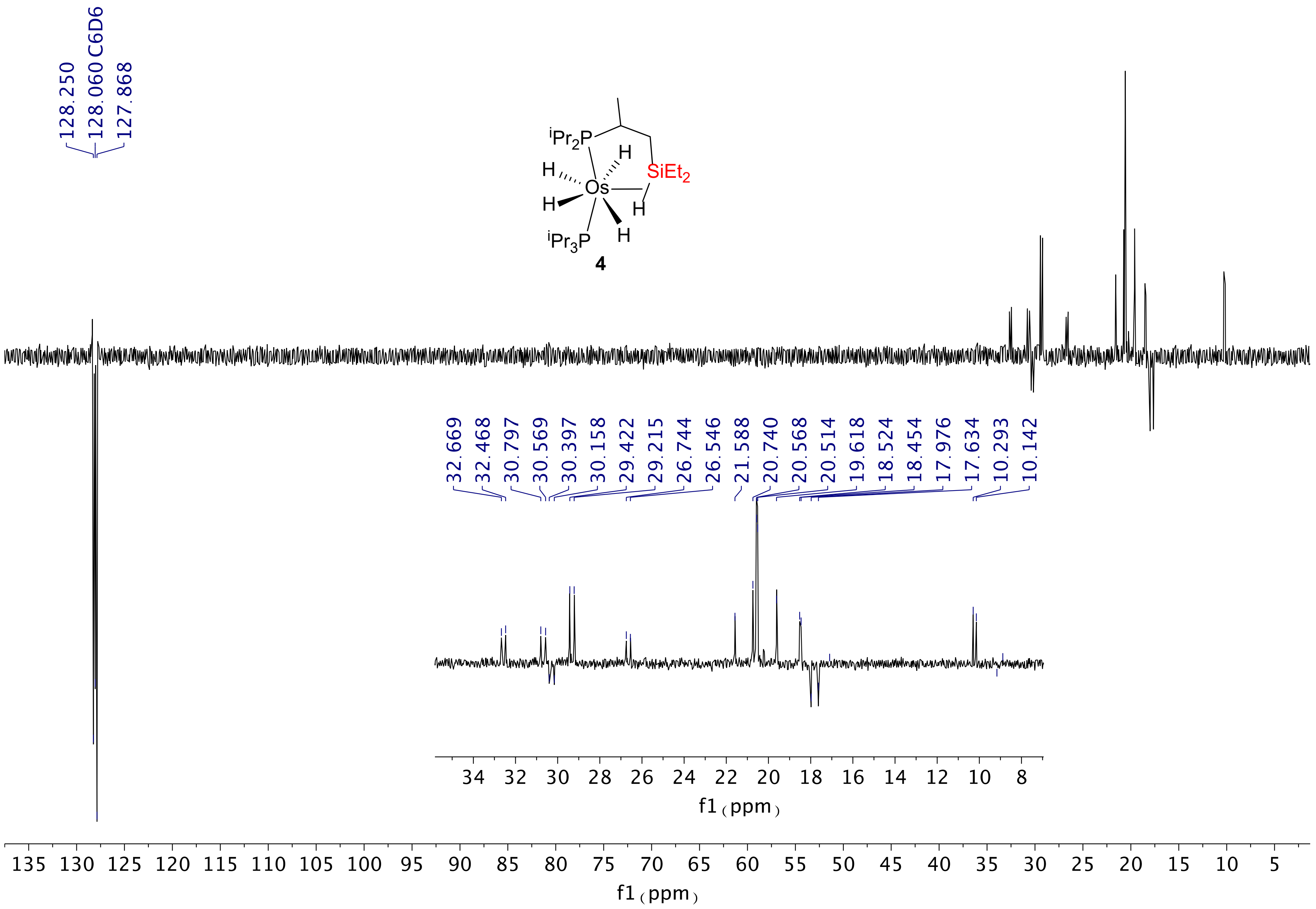

Figure S17. $^{13}C\{^{1}H\}$-apt NMR (125.77 MHz, C_6D_6, 298 K) spectrum of **4**.

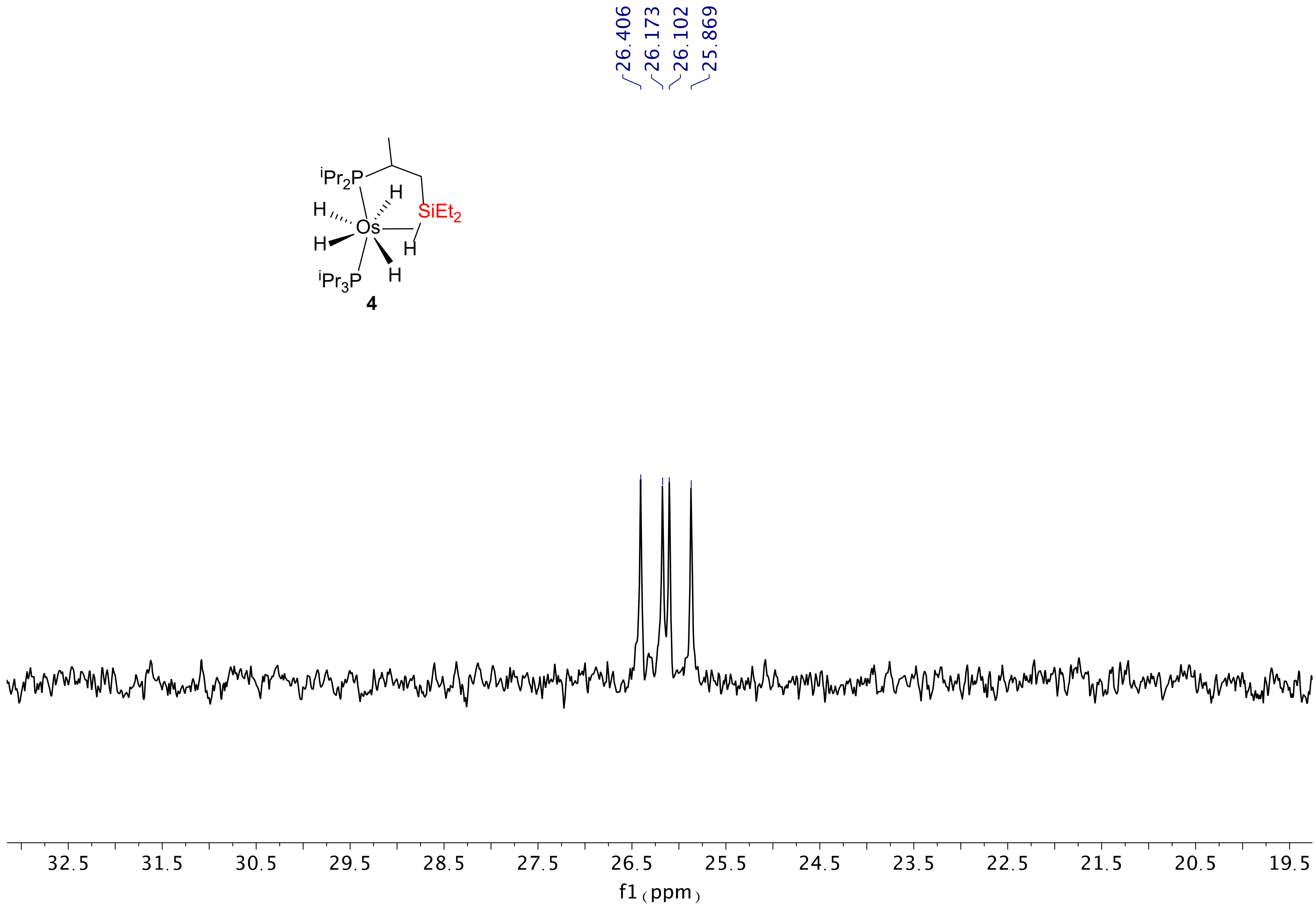

Figure S18. $^{29}Si\{^1H\}$ NMR (59.63 MHz, C_6D_6, 298 K) spectrum of **4**.

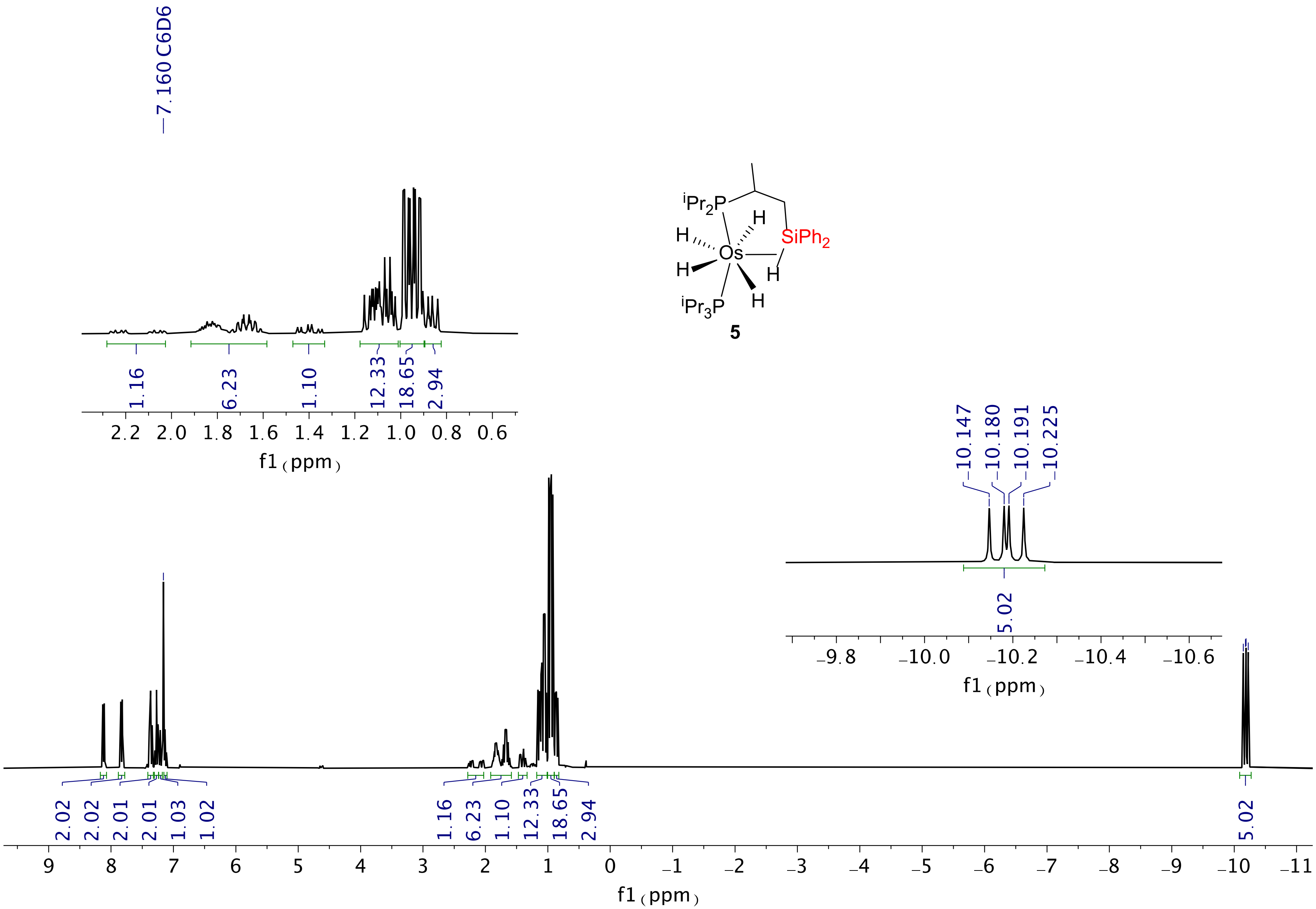

Figure S19. ^{1}H NMR (300.13 MHz, C_6D_6, 298 K) spectrum of **5**.

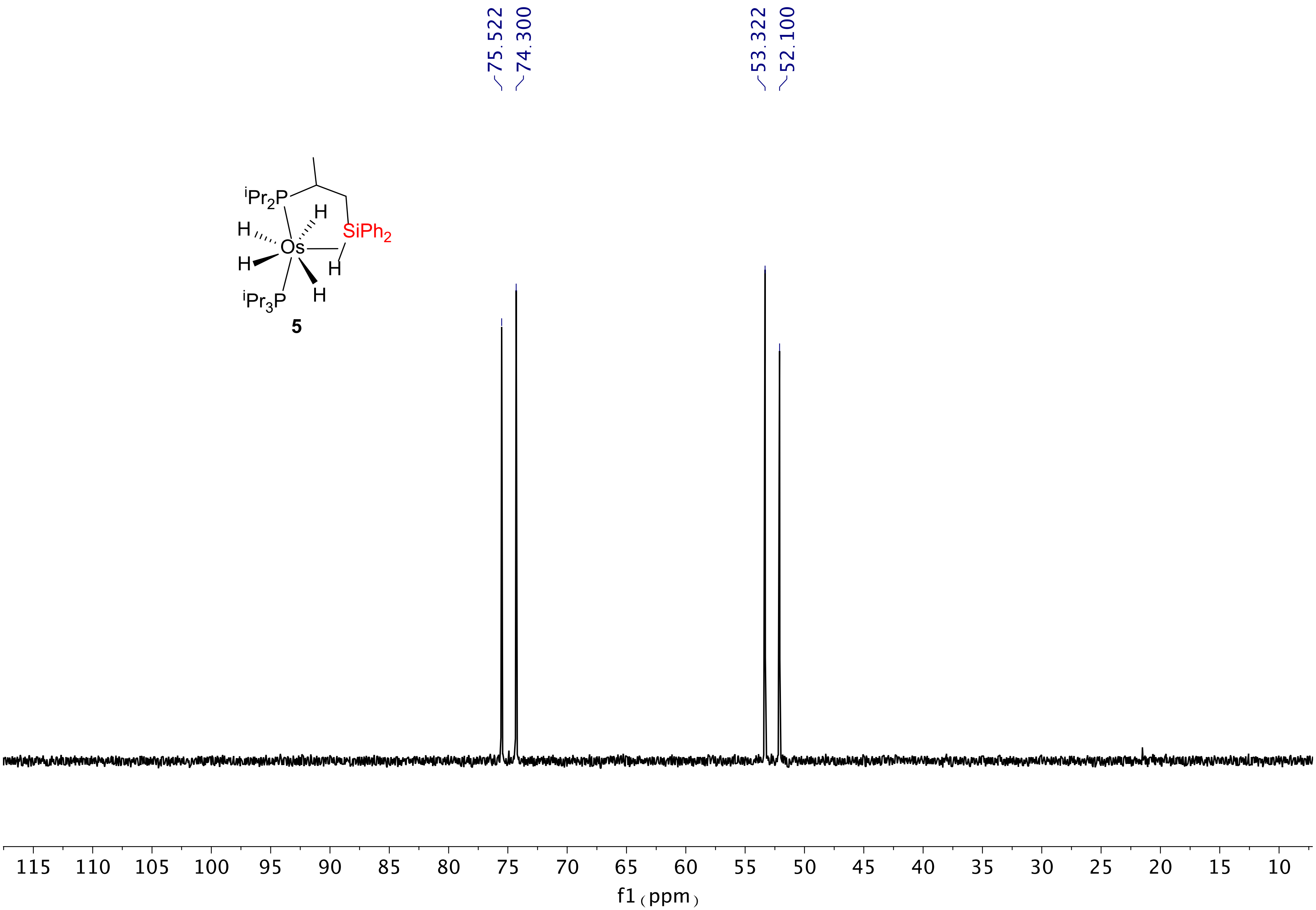

Figure S20. $^{31}P\{^1H\}$ NMR (121.50 MHz, C_6D_6, 298 K) spectrum of **5**.

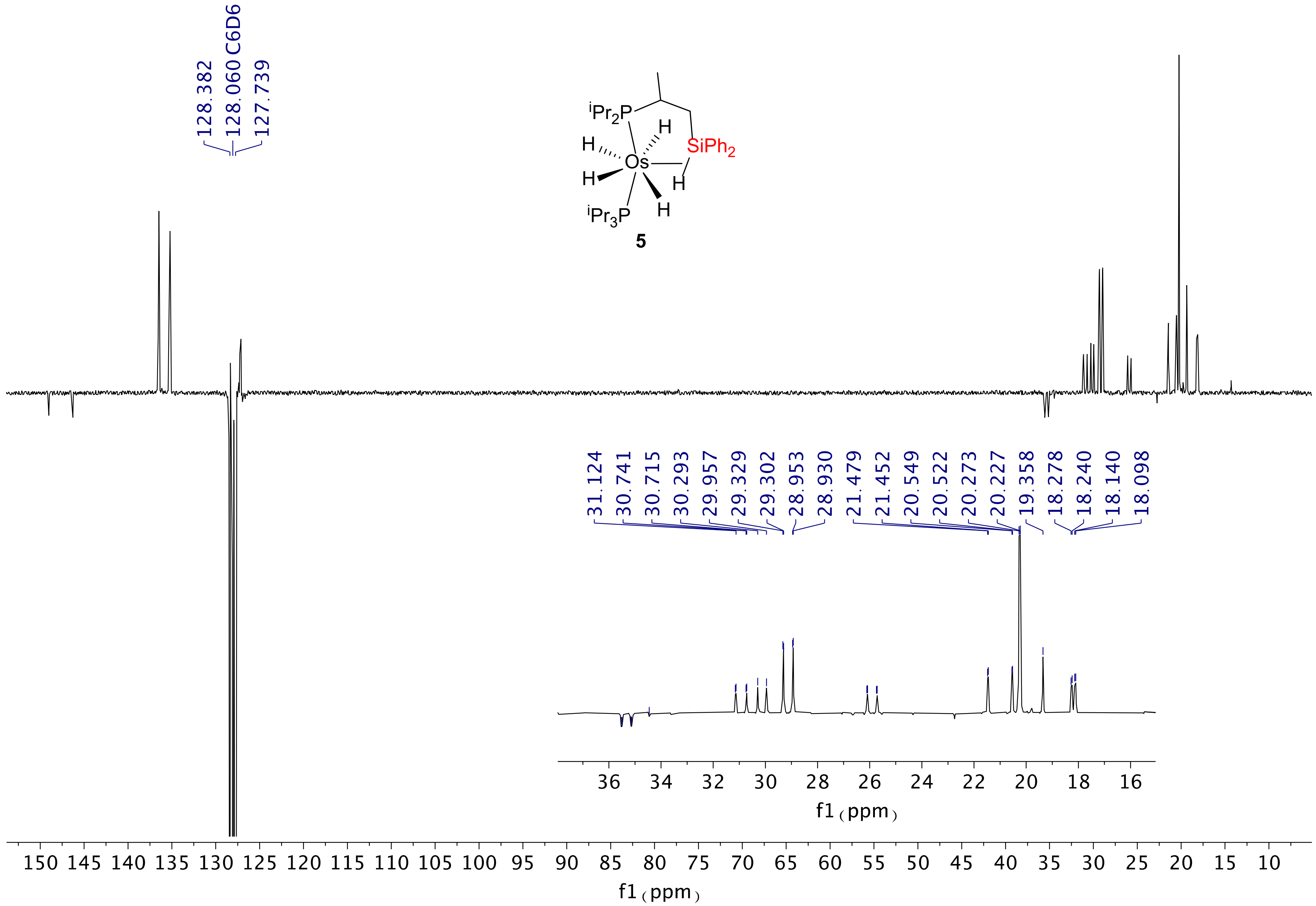

Figure S21. $^{13}C\{^1H\}$-apt NMR (75 MHz, C_6D_6, 298 K) spectrum of **5**.

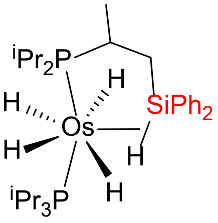

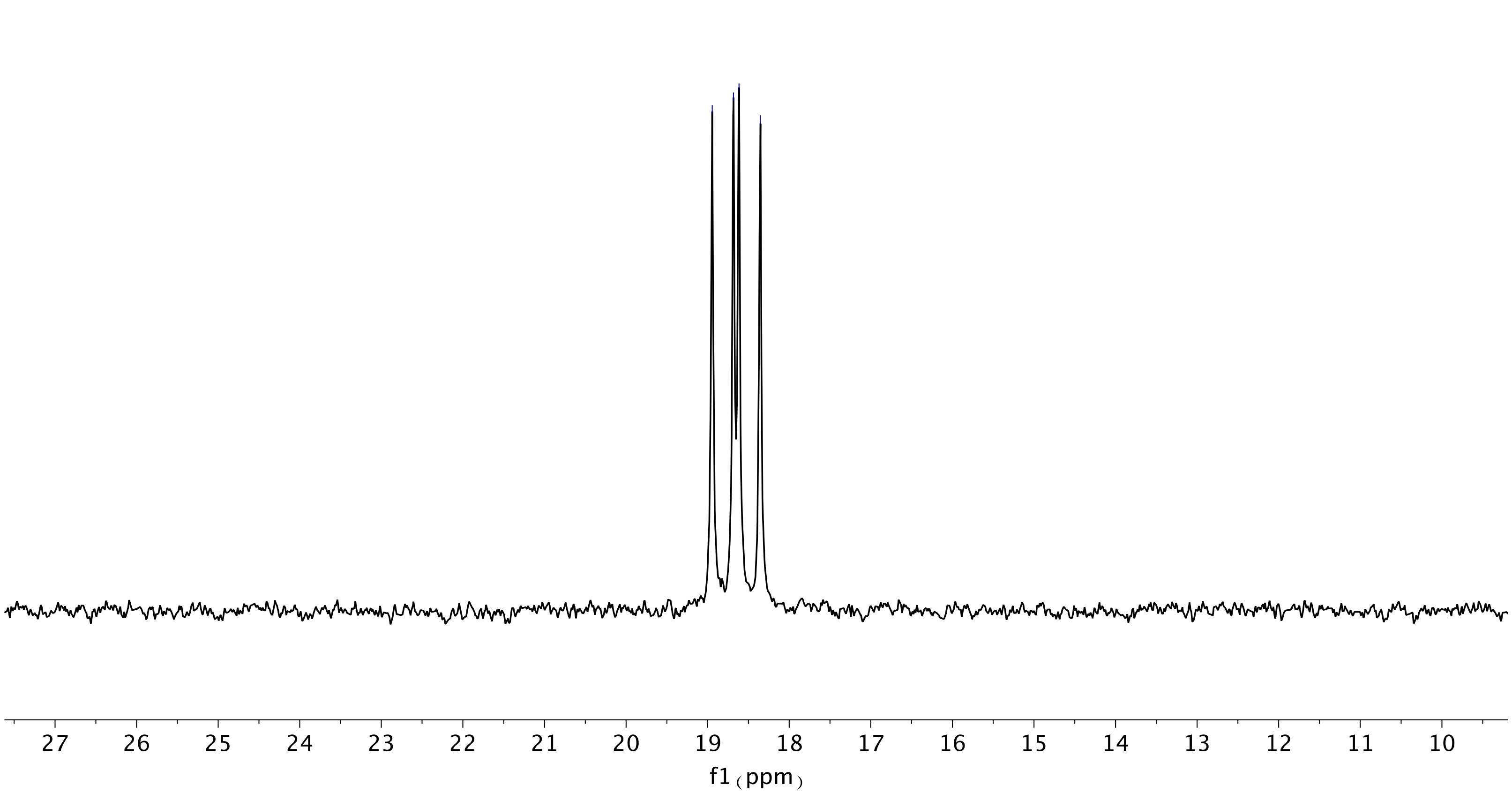

Figure S22. $^{29}Si\{^{1}H\}$ NMR (59.63 MHz, C_6D_6, 298 K) spectrum of **5**.

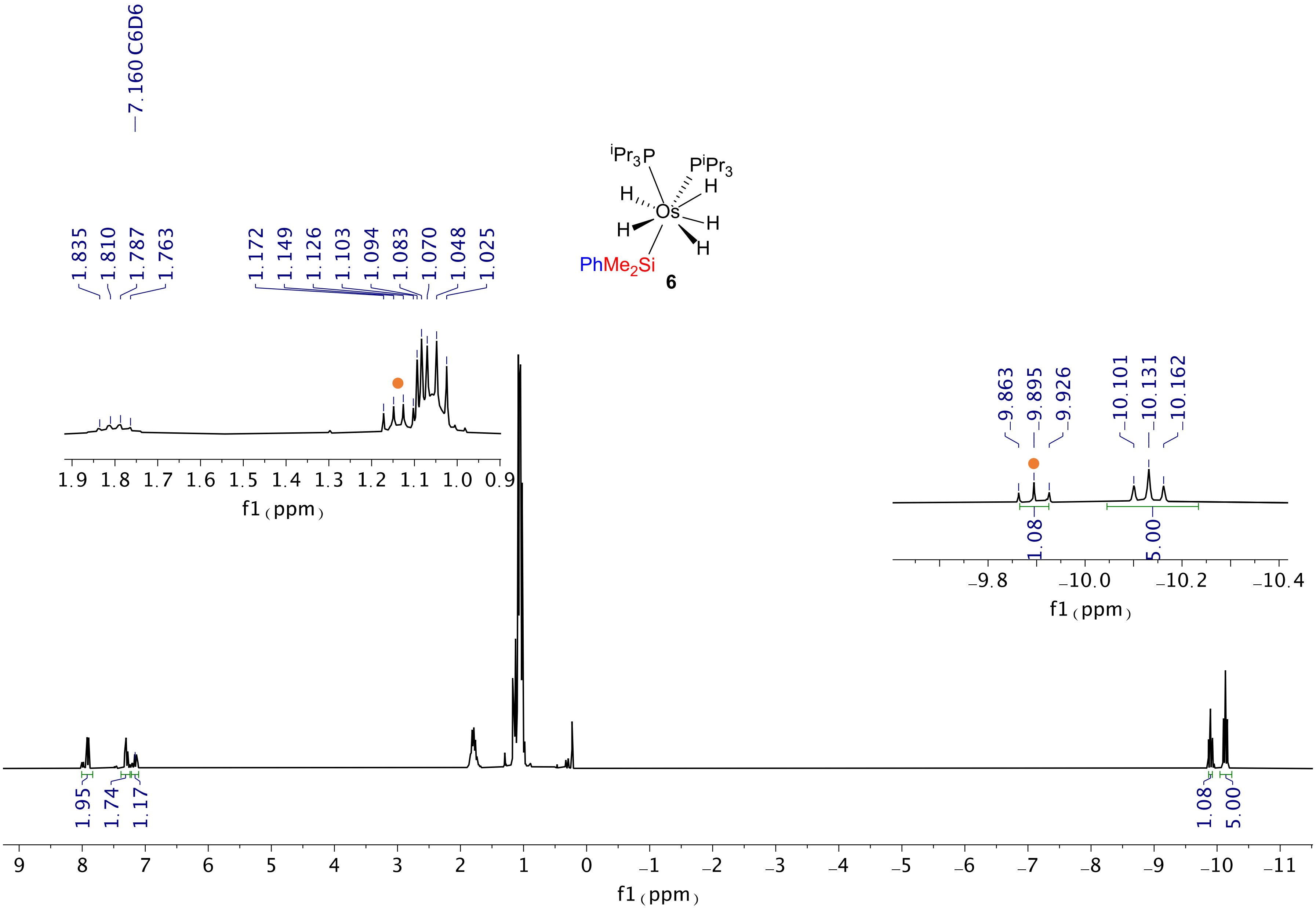

Figura S23. ^{1}H NMR (300.13 MHz, C_6D_6, 298 K) spectrum of **6**. ● $OsH_6(P^iPr_3)_2$ (**1**).

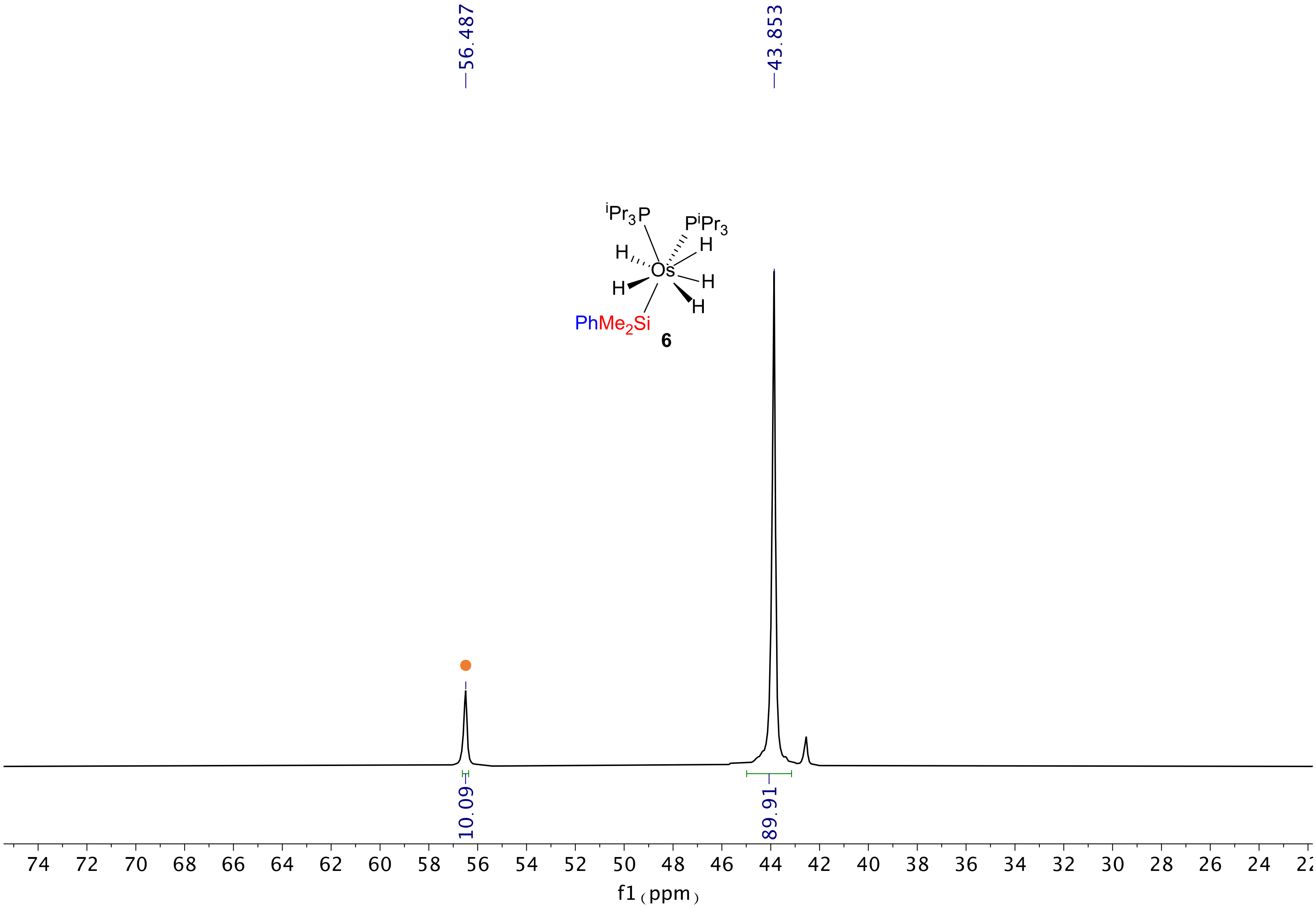

Figure S24. $^{31}P\{^{1}H\}$ NMR (121.50 MHz, C_6D_6, 298 K) spectrum of **6**. ● $OsH_6(P^iPr_3)_2$ (**1**).

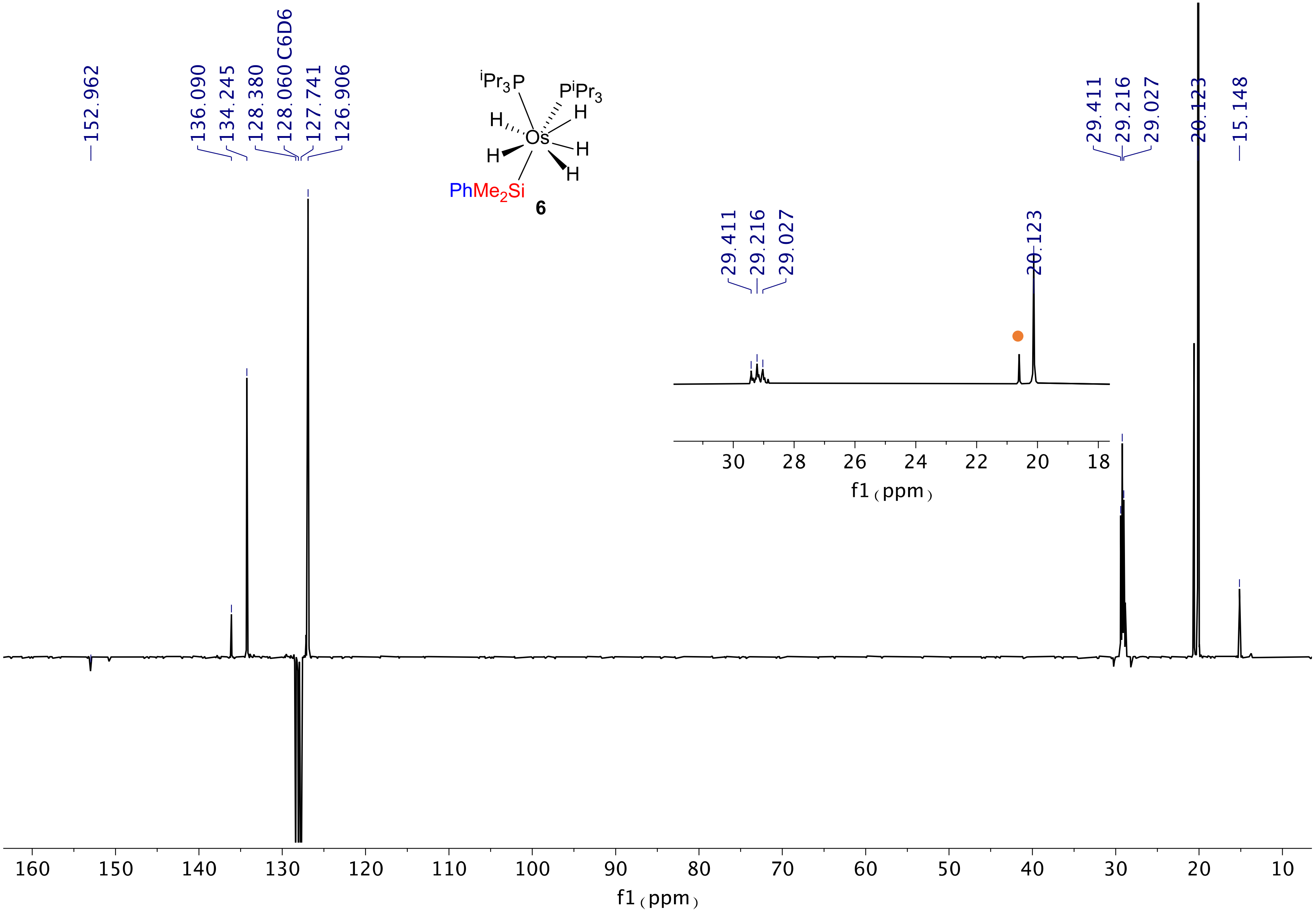

Figure S25. $^{13}C\{^1H\}$-apt NMR (75 MHz, C_6D_6, 298 K) spectrum of **6**. ● $OsH_6(P^iPr_3)_2$ (**1**).

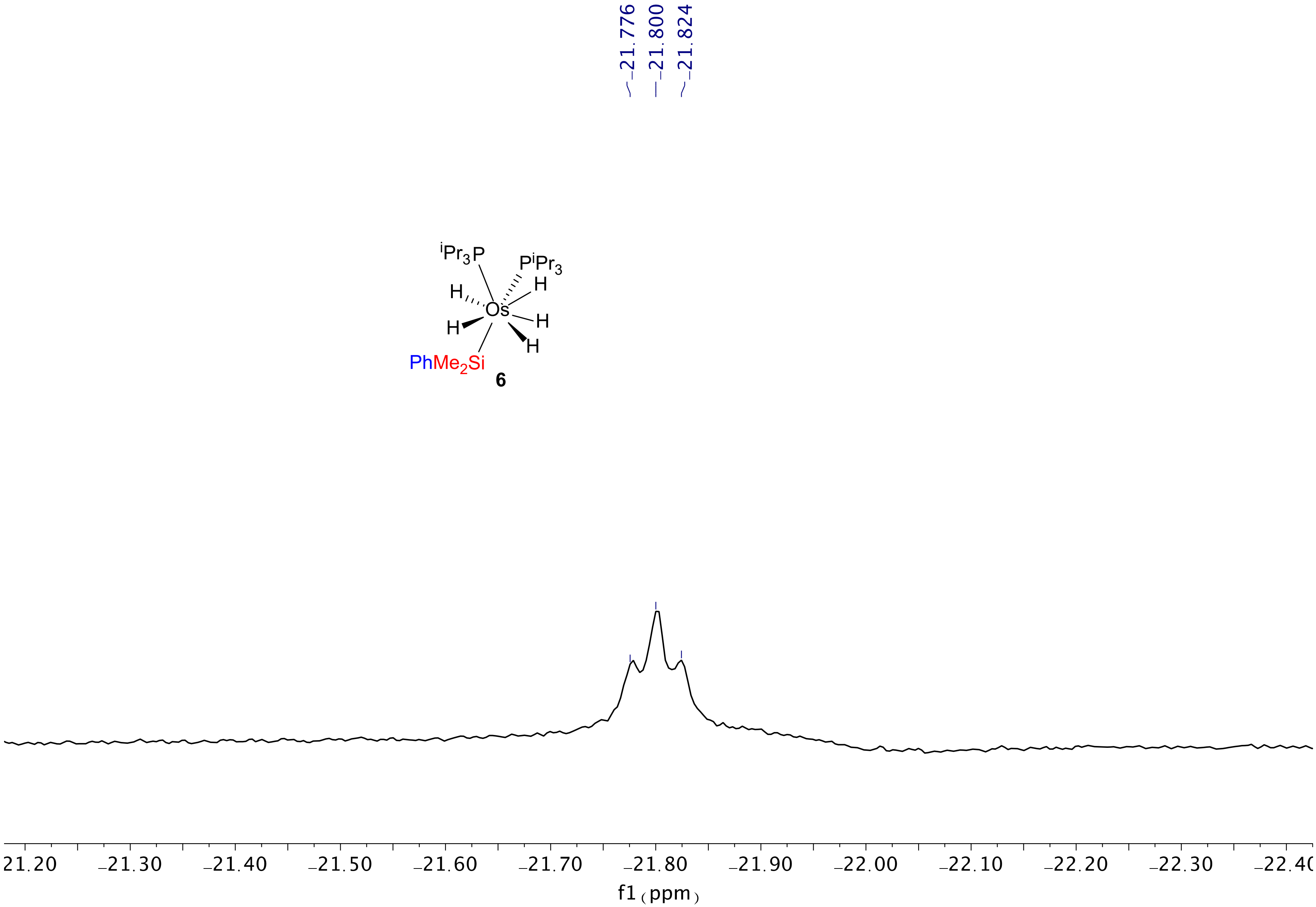

Figure S26. $^{29}Si\{^1H\}$ NMR (59.63 MHz, C_6D_6, 308 K) spectrum of **6**.

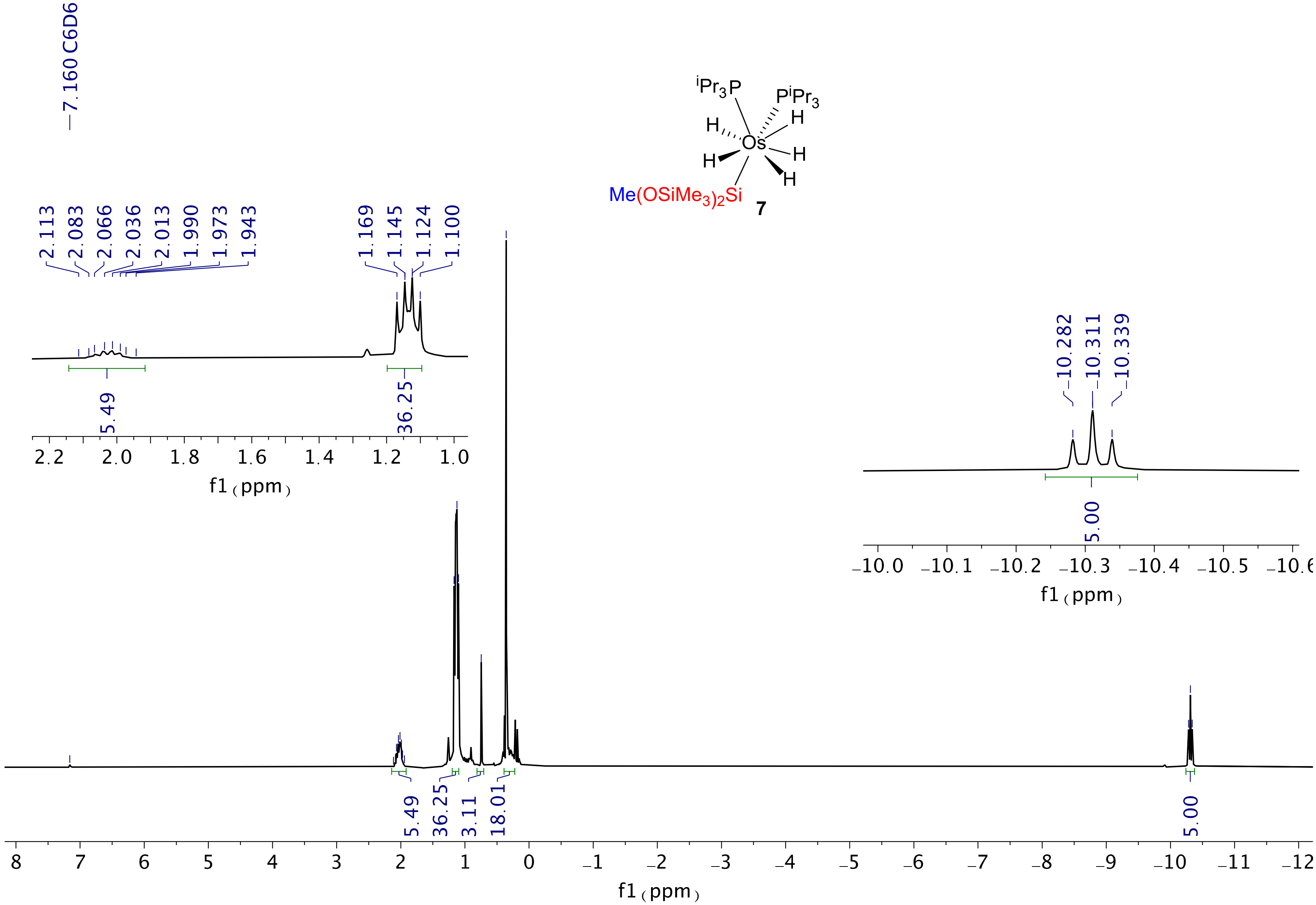

Figure S27. ^{1}H NMR (300.13 MHz, C_6D_6, 298 K) spectrum of **7**.

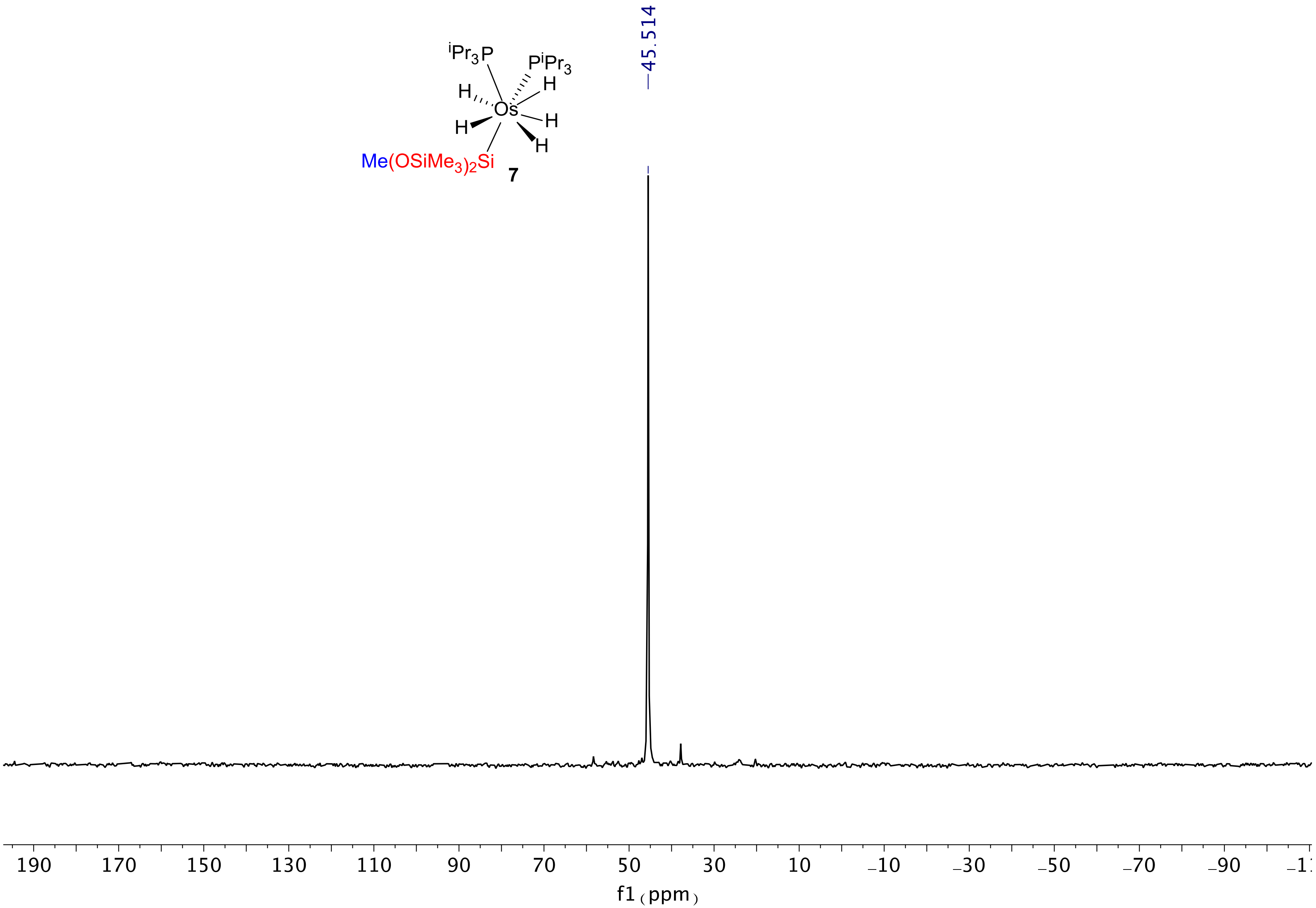

Figure S28. $^{31}P\{^{1}H\}$ NMR (121.50 MHz, C_6D_6, 298 K) spectrum of **7**.

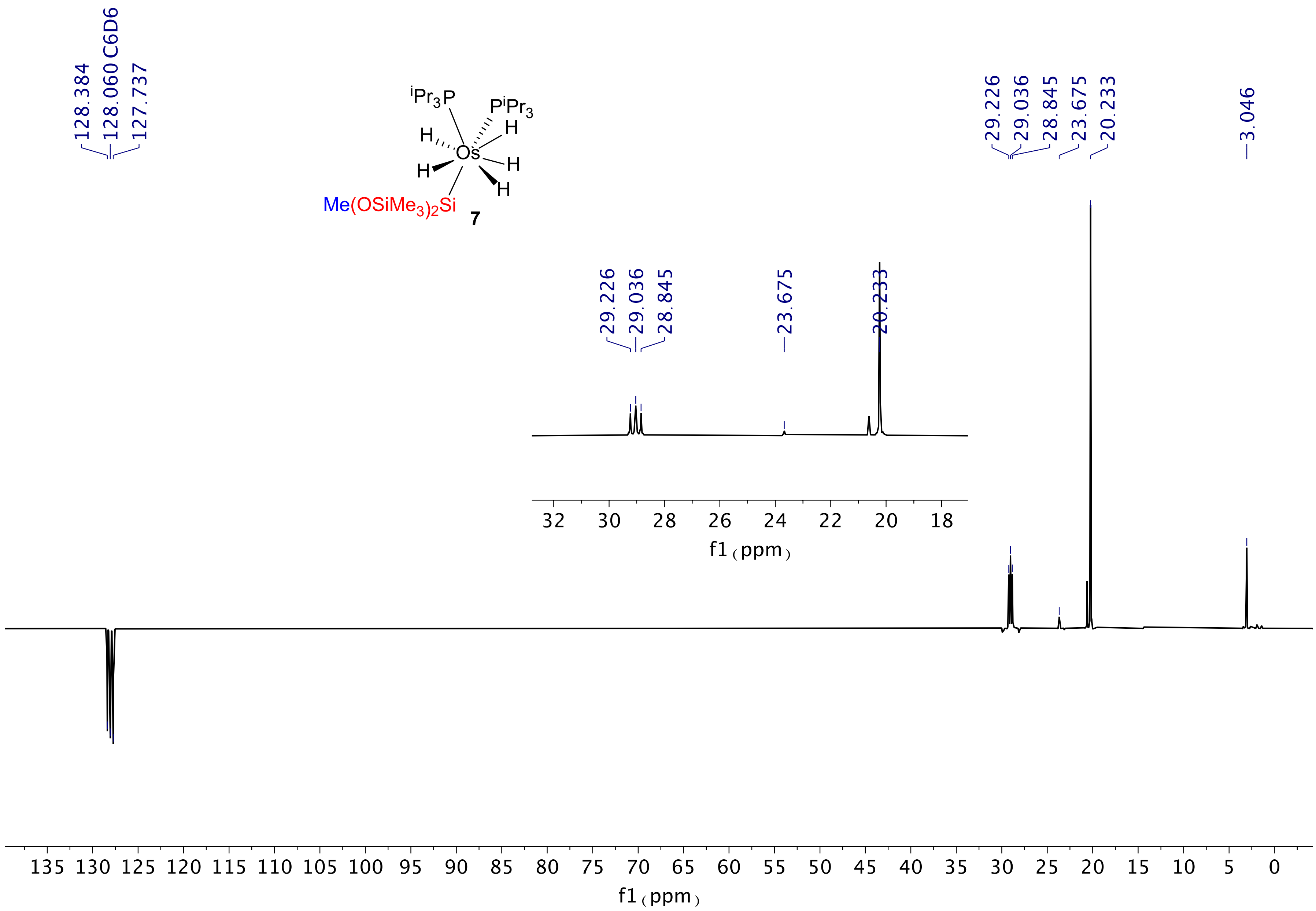

Figure S29. $^{13}C\{^1H\}$-apt NMR (75 MHz, C_6D_6, 298 K) spectrum of **7**.

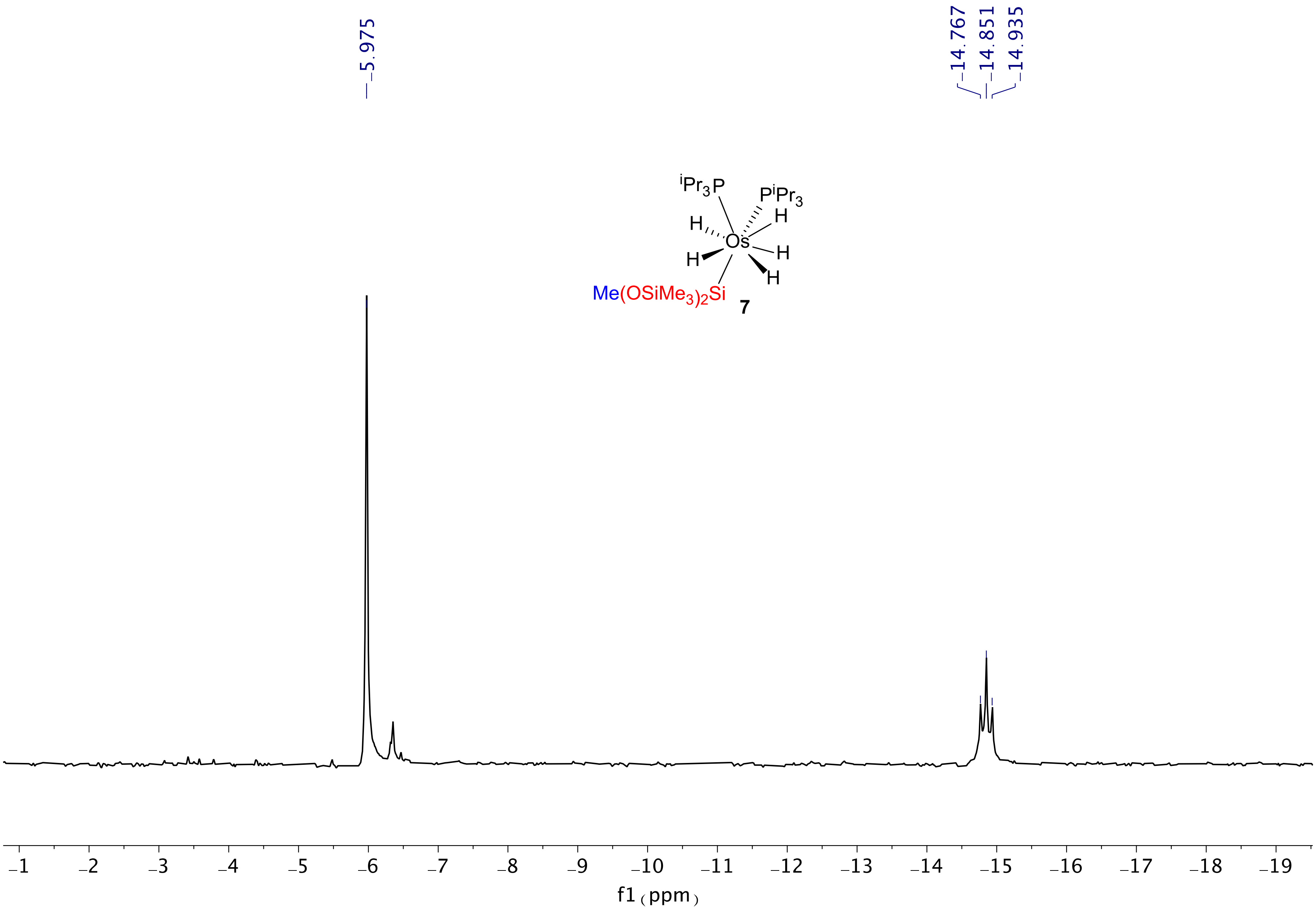

Figure S30. $^{29}Si\{^{1}H\}$ NMR (59.63 MHz, C_6D_6, 298 K) spectrum of **7**.

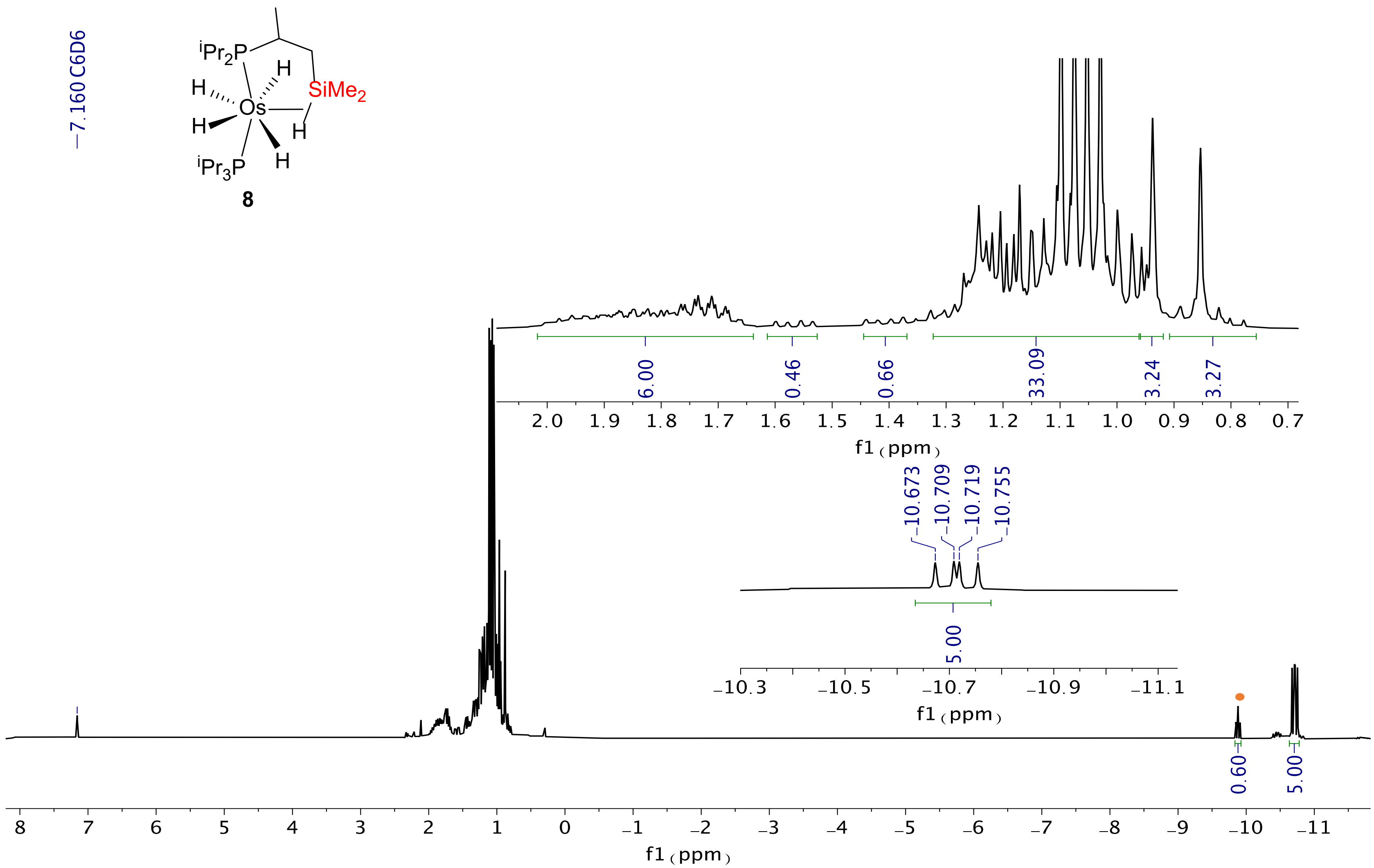

Figure S31. ^{1}H NMR (300.13 MHz, C_6D_6, 298 K) spectrum of **8**. ● $OsH_6(P^iPr_3)_2$ (**1**).

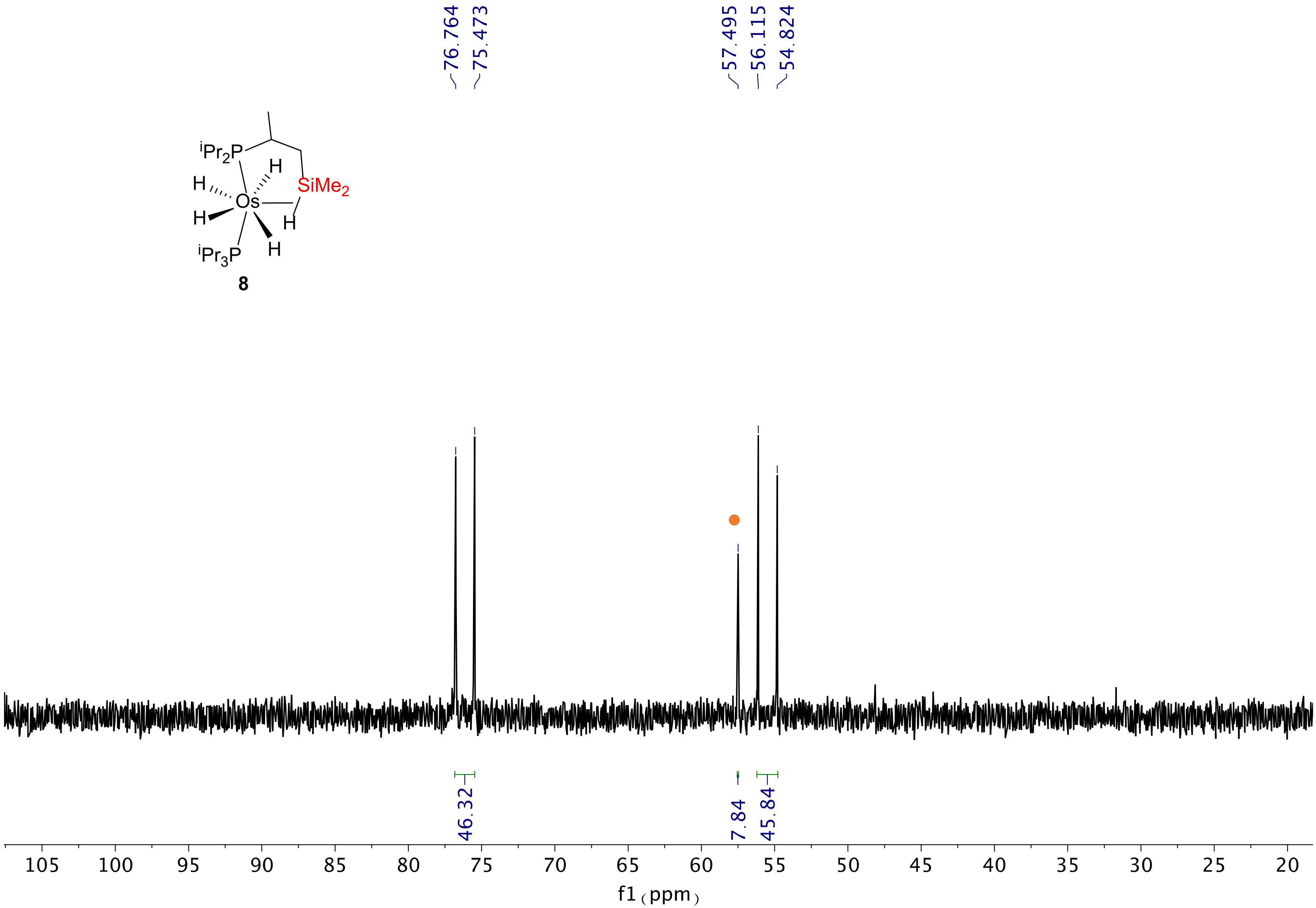

Figure S32. $^{31}P\{^{1}H\}$ NMR (121.50 MHz, C_6D_6, 298 K) spectrum of **8**. ● $OsH_6(P^iPr_3)_2$ (**1**).

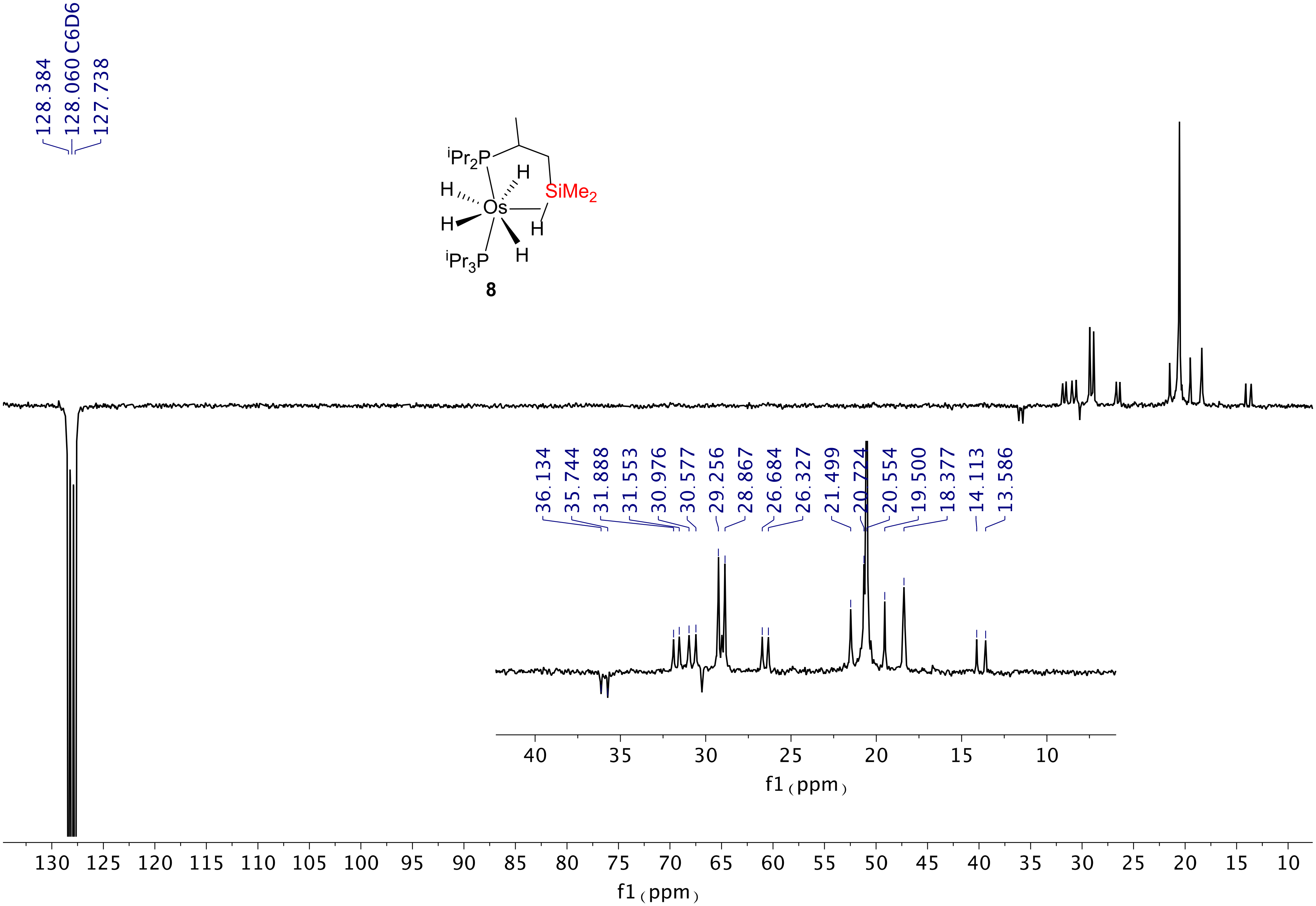

Figure S33. $^{13}C\{^{1}H\}$-apt NMR (75 MHz, C_6D_6, 298 K) spectrum of **8**.

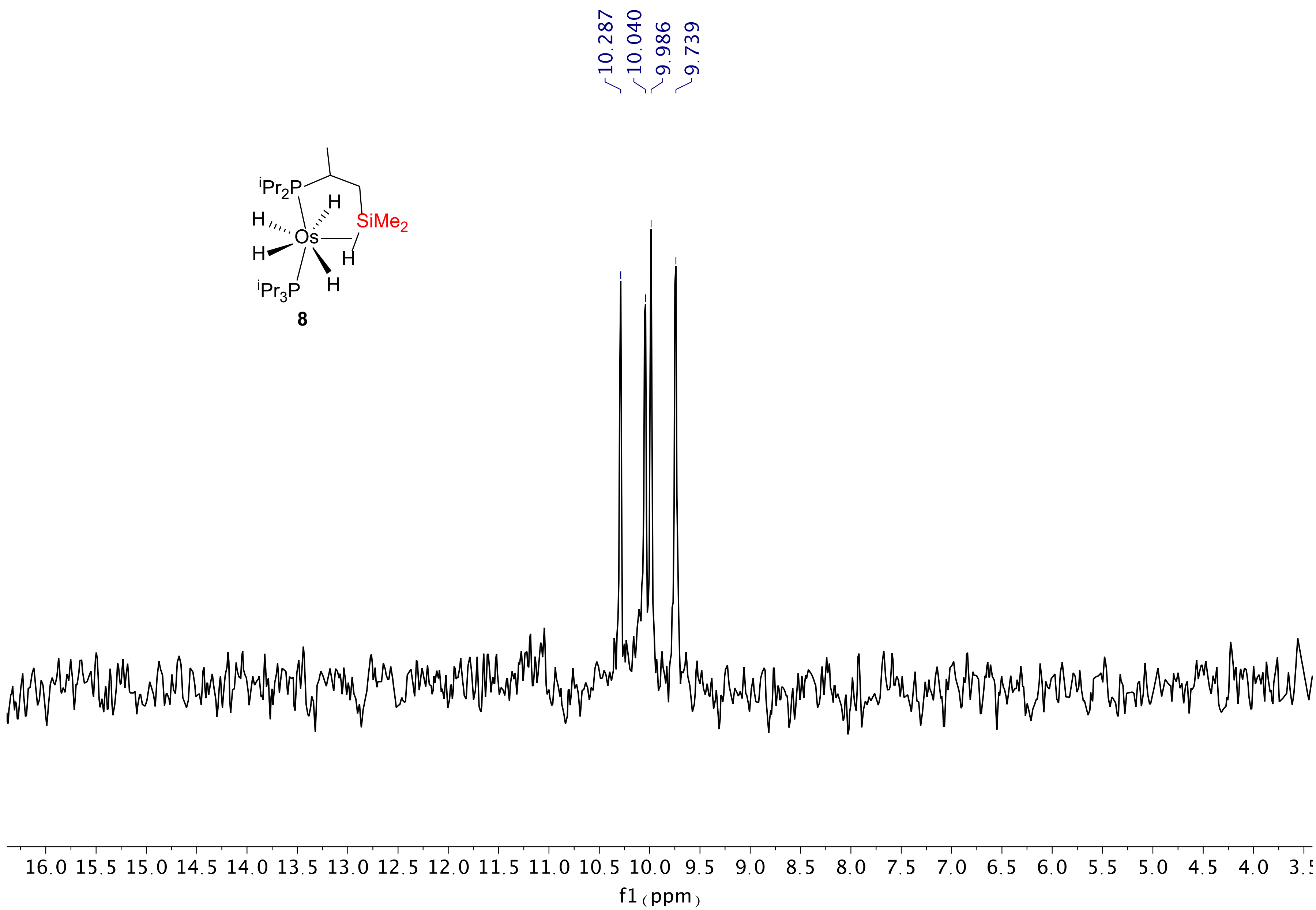

Figure S34. $^{29}Si\{^1H\}$ NMR (59.63 MHz, C_6D_6, 298 K) spectrum of **8**.

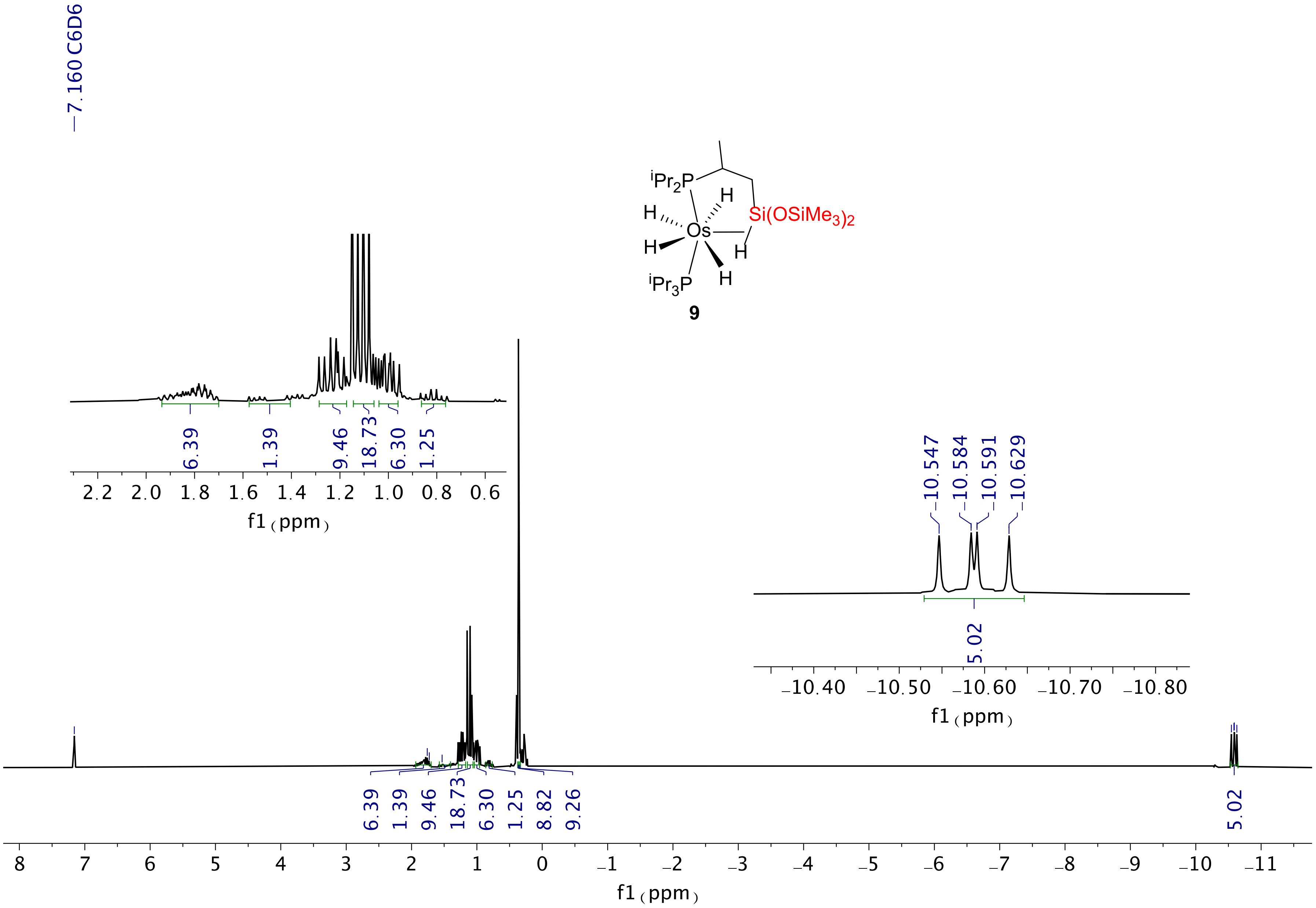

Figure S35. ^{1}H NMR (300.13 MHz, C_6D_6, 298 K) spectrum of **9**.

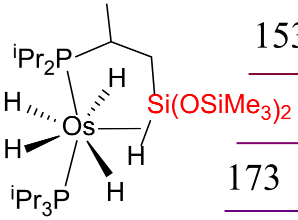

9

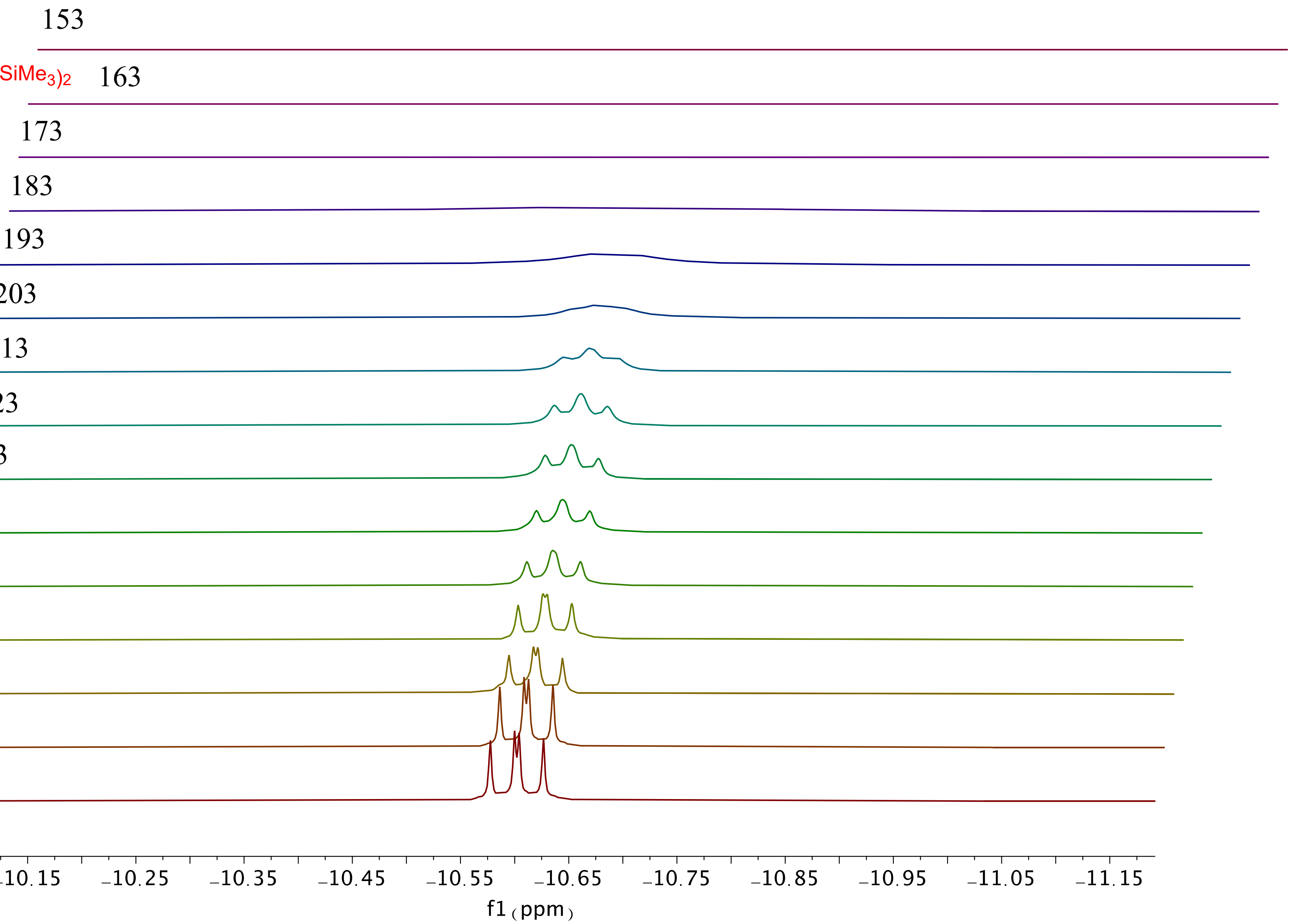

Figure S36. High-field region of the ^{1}H NMR (500.12 MHz, Methylcyclohexane-d_{14}) spectrum of **9** between 298 and 153 K.

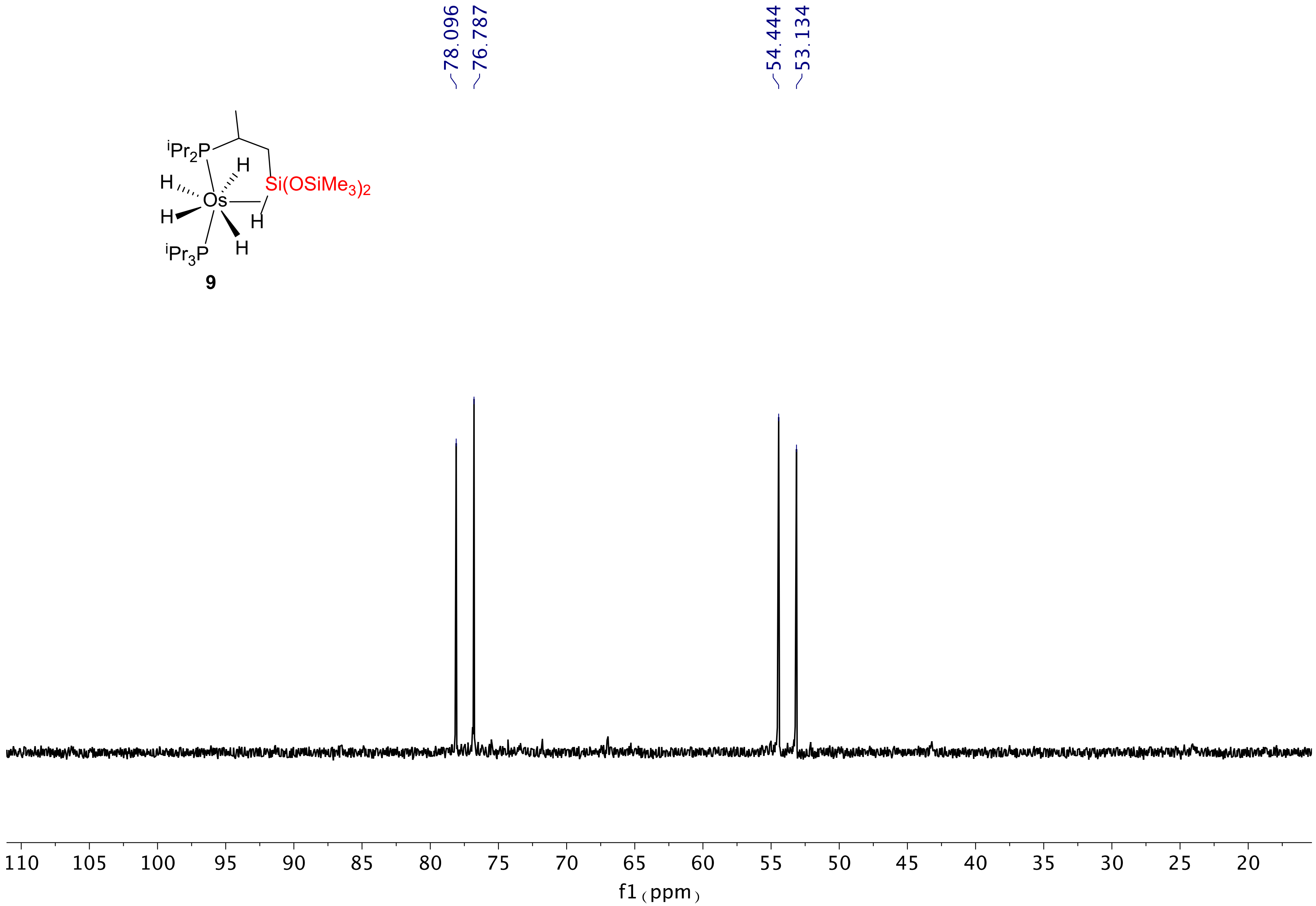

Figure S37. $^{31}P\{^1H\}$ NMR (121.50 MHz, C_6D_6, 298 K) spectrum of **9**.

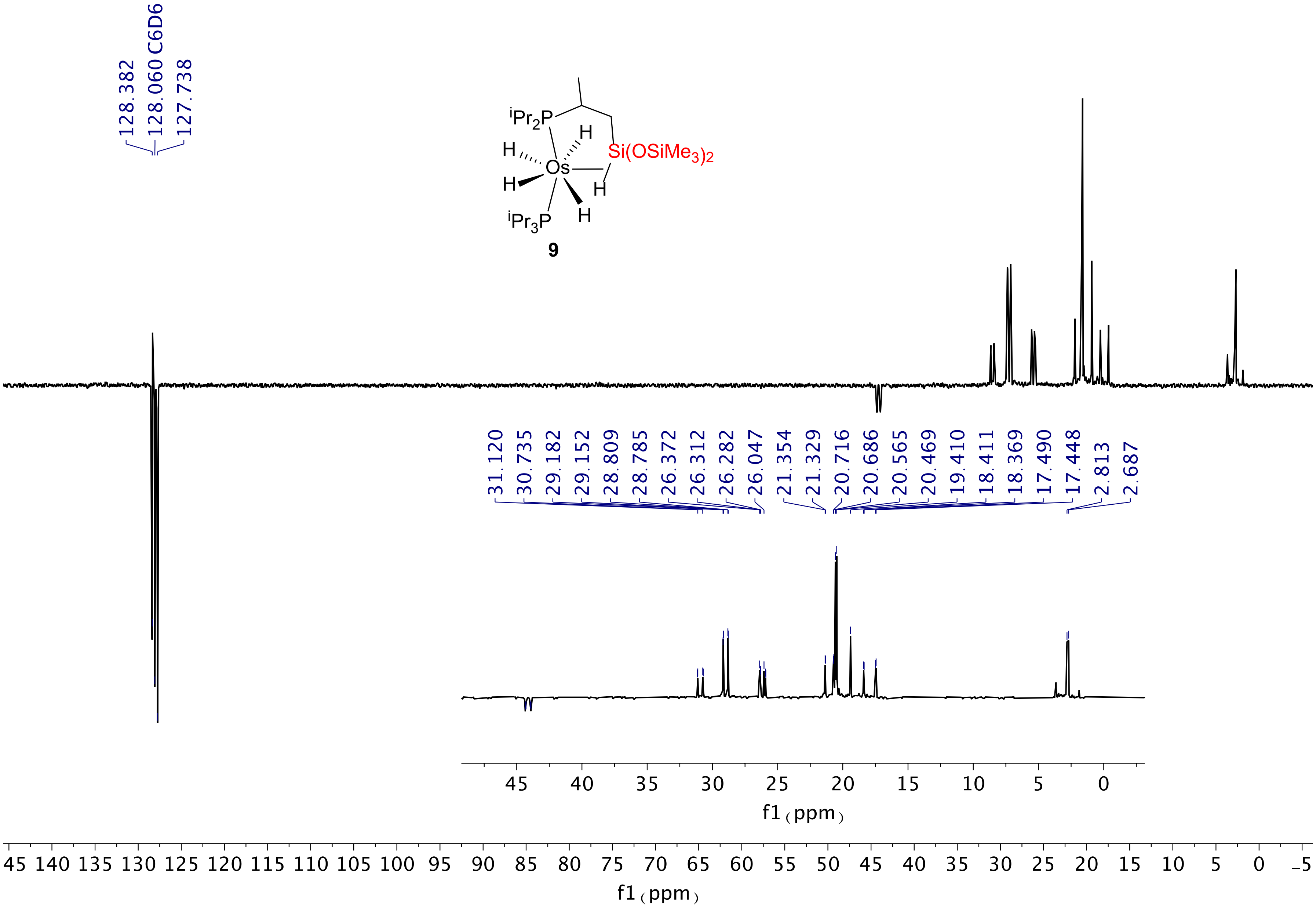

Figure S38. $^{13}C\{^1H\}$-apt NMR (75 MHz, C_6D_6, 298 K) spectrum of **9**.

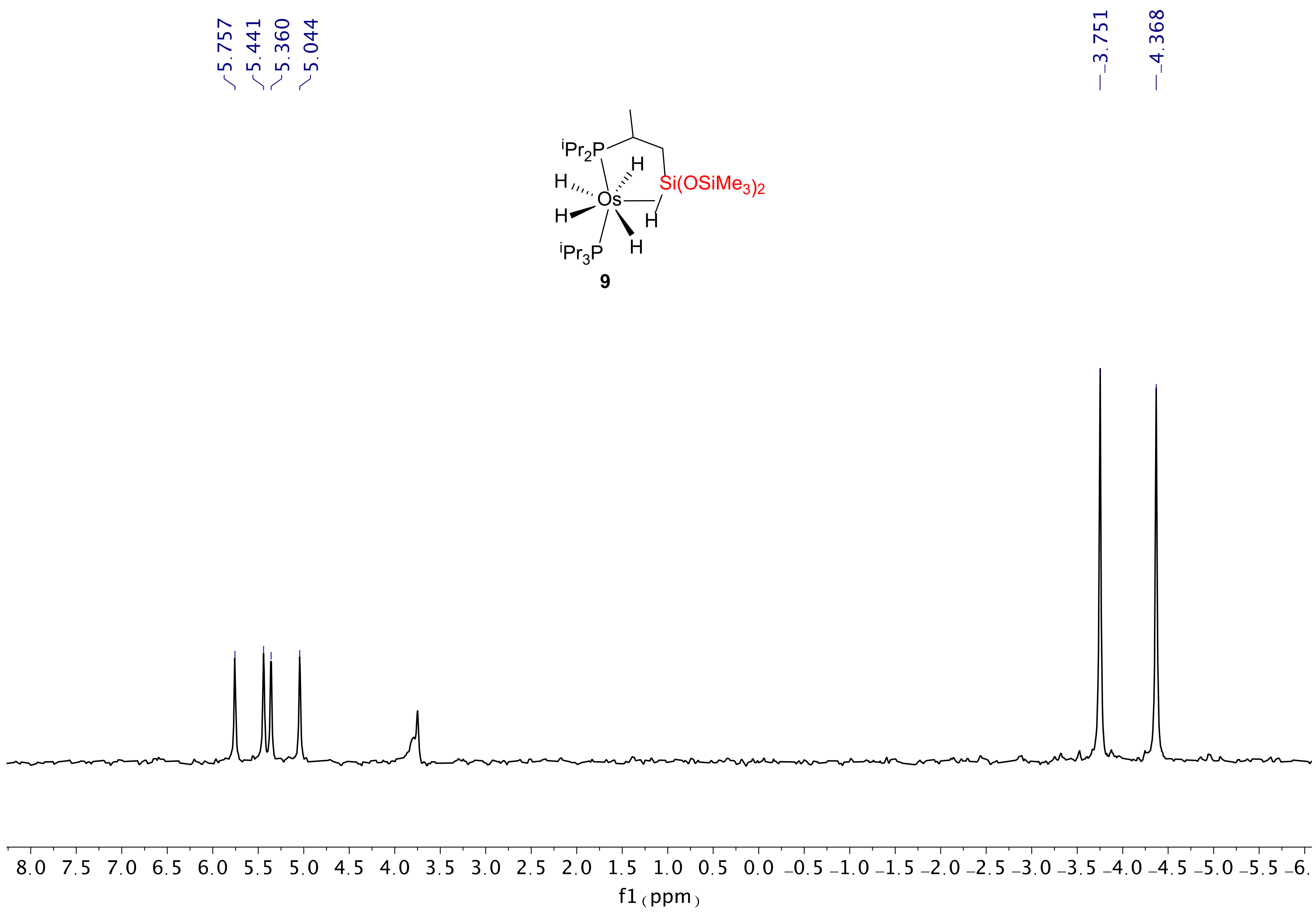

FFigure S39. $^{29}Si\{^{1}H\}$ NMR (59.63 MHz, C_6D_6, 298 K) spectrum of **9**.

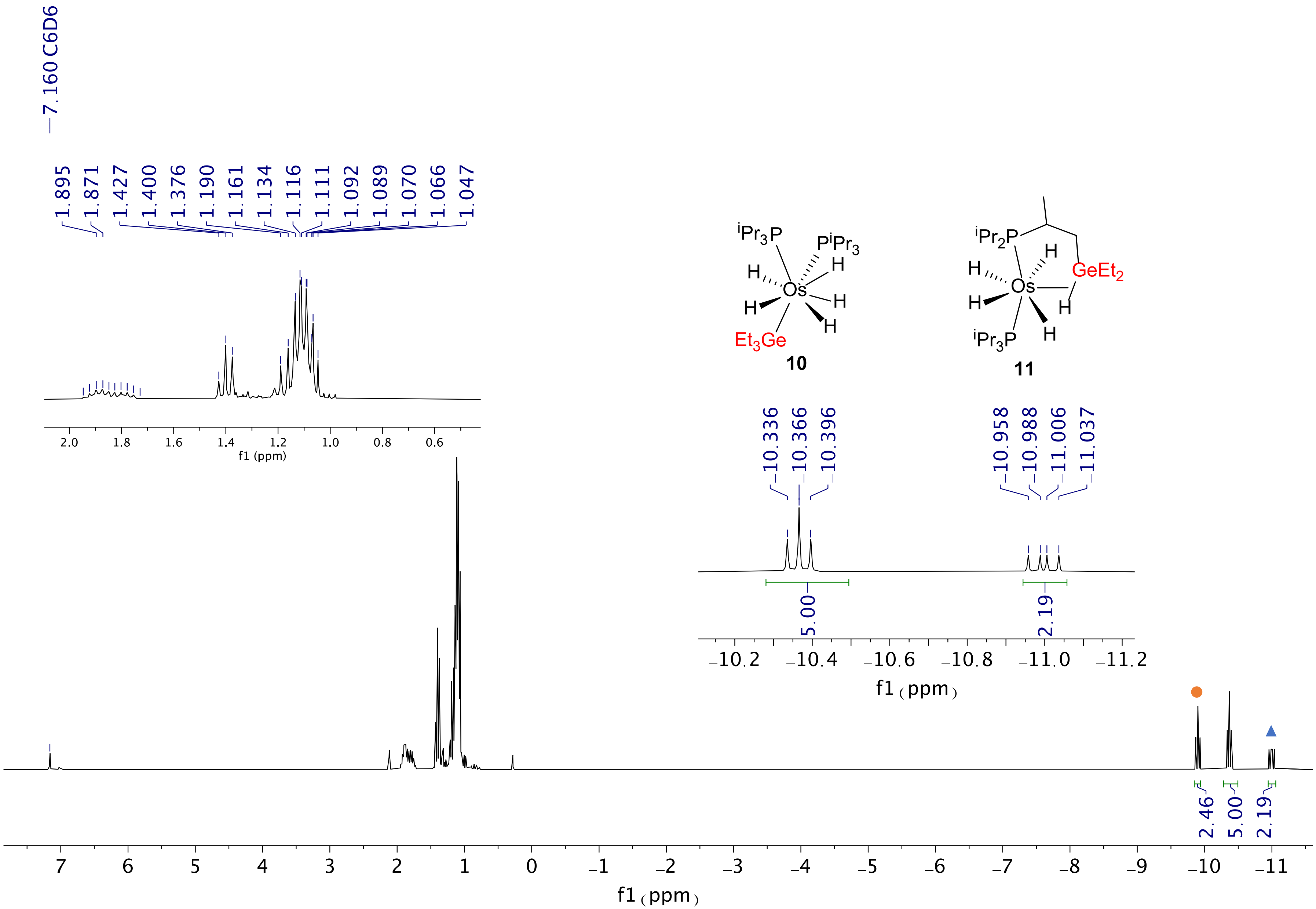

Figure S40. ^{1}H NMR (300.13 MHz, C_6D_6, 298 K) spectrum of the reaction of **1** with Et_3GeH (after heating in n-octane at 50 ºC for 24 h): Formation of **10** and **11** in a 56:24 molar ratio. ● $OsH_6(P^iPr_3)_2$ (**1**). ▲ Complex (**11**).

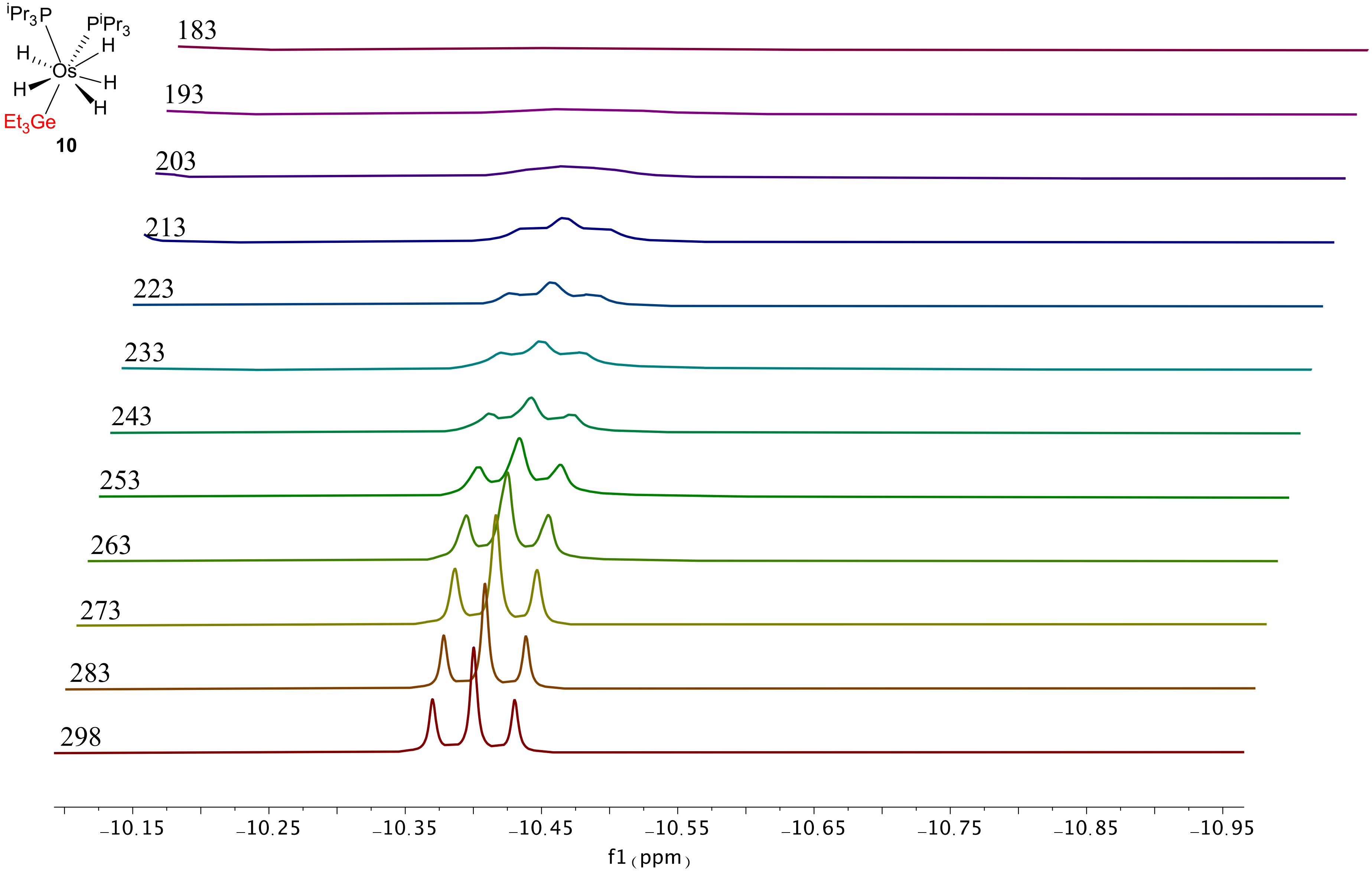

Figure S41. High-field region of the ^{1}H NMR (300.13 MHz, Toluene-d_8) spectrum of complex **10** between 298 and 183 K.

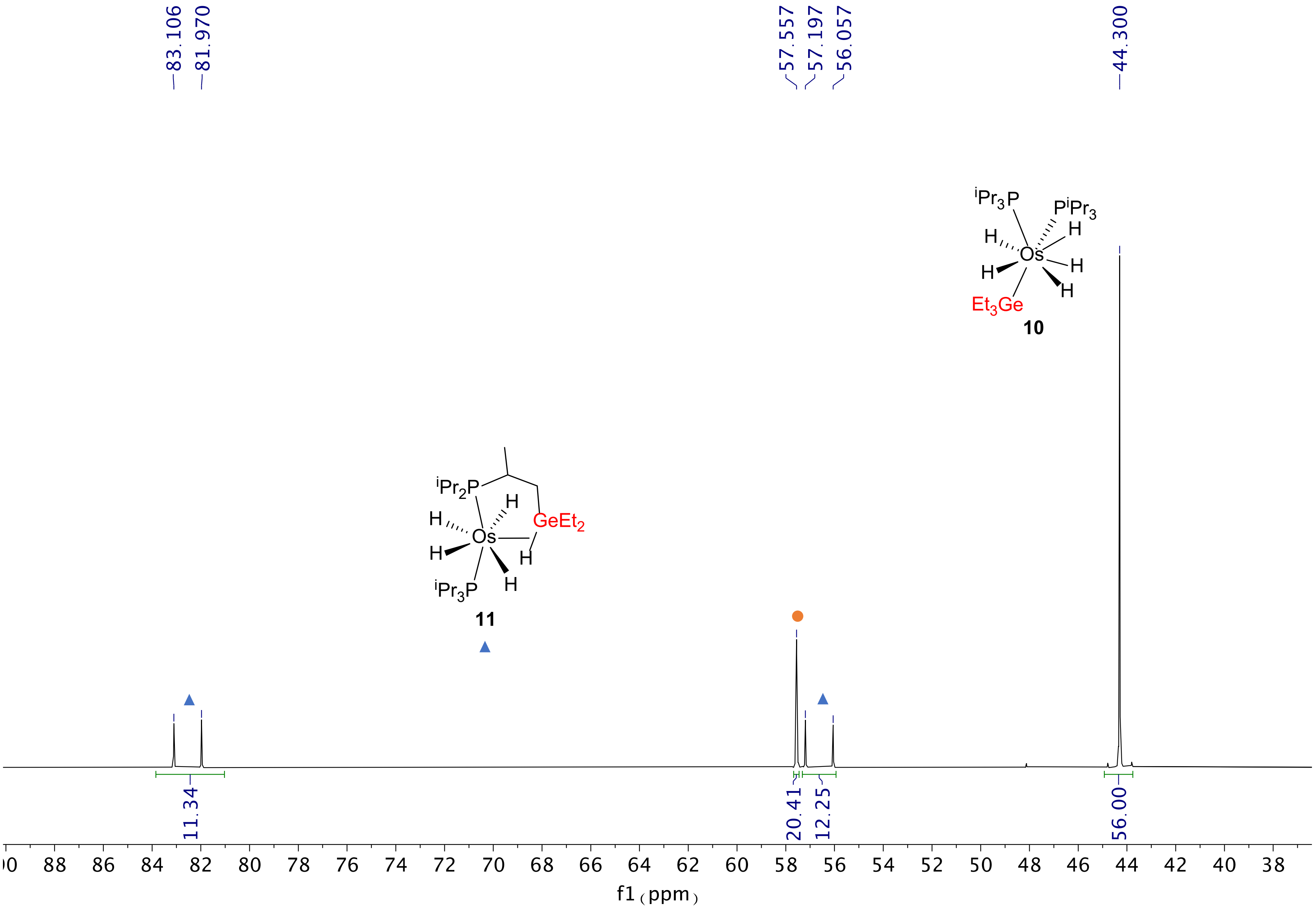

Figure S42. $^{31}P\{^1H\}$ NMR (121.50 MHz, C_6D_6, 298 K) spectrum spectrum of the reaction of **1** with Et_3GeH (after heating in n-octane at 50 ºC for 24 h): Formation of **10** and **11** in a 56:24 molar ratio. ● $OsH_6(P^iPr_3)_2$ (**1**). ▲ Complex (**11**).

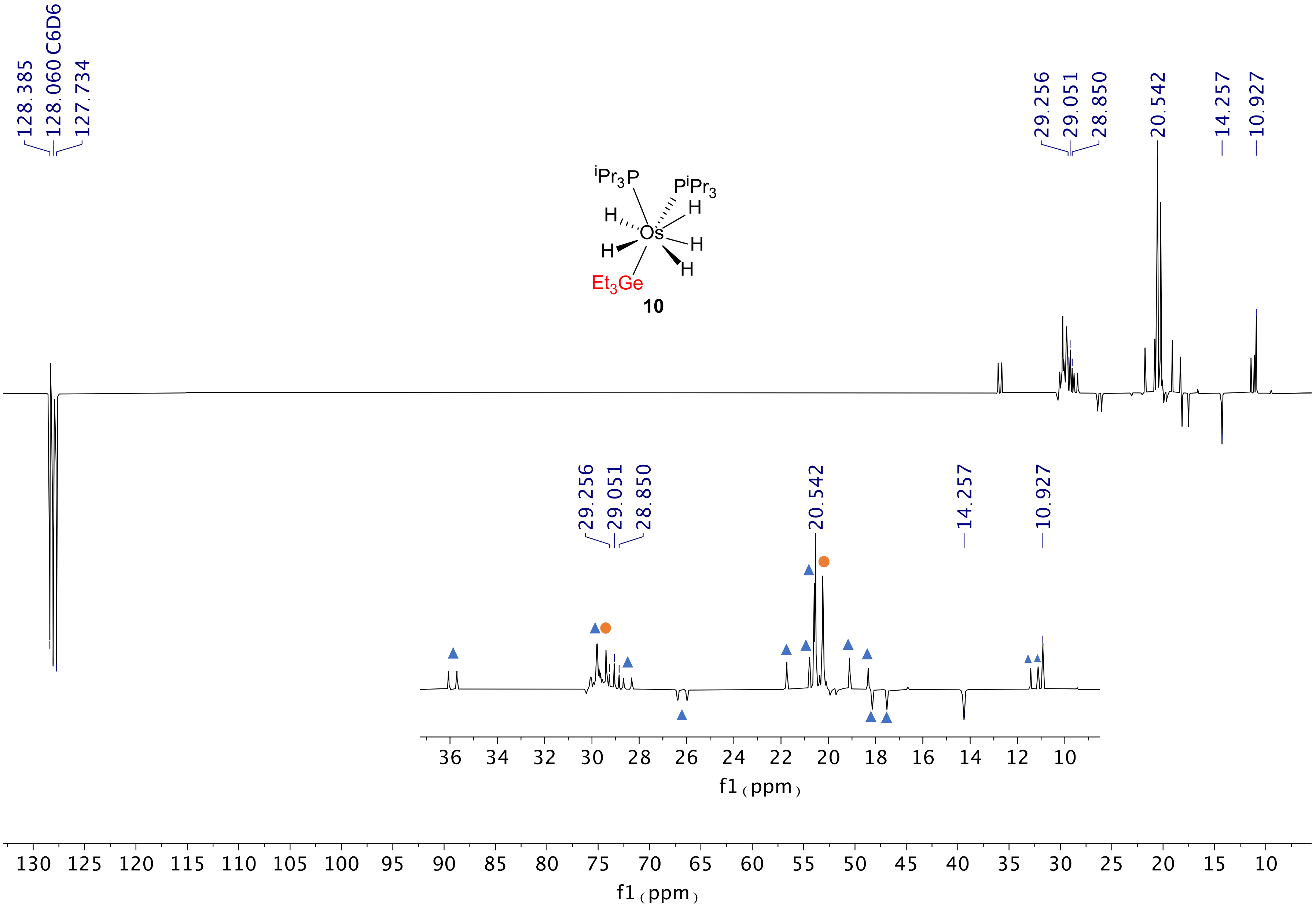

Figure S43. $^{13}C\{^{1}H\}$-apt NMR (75 MHz, C_6D_6, 298 K) spectrum of the reaction of **1** with Et_3GeH (after heating in n-octane at 50 ºC for 24 h. Formation of **10** and **11** in a 56:24 molar ratio. ● $OsH_6(P^{i}Pr_3)_2$ (**1**). ▲ Complex (**11**).

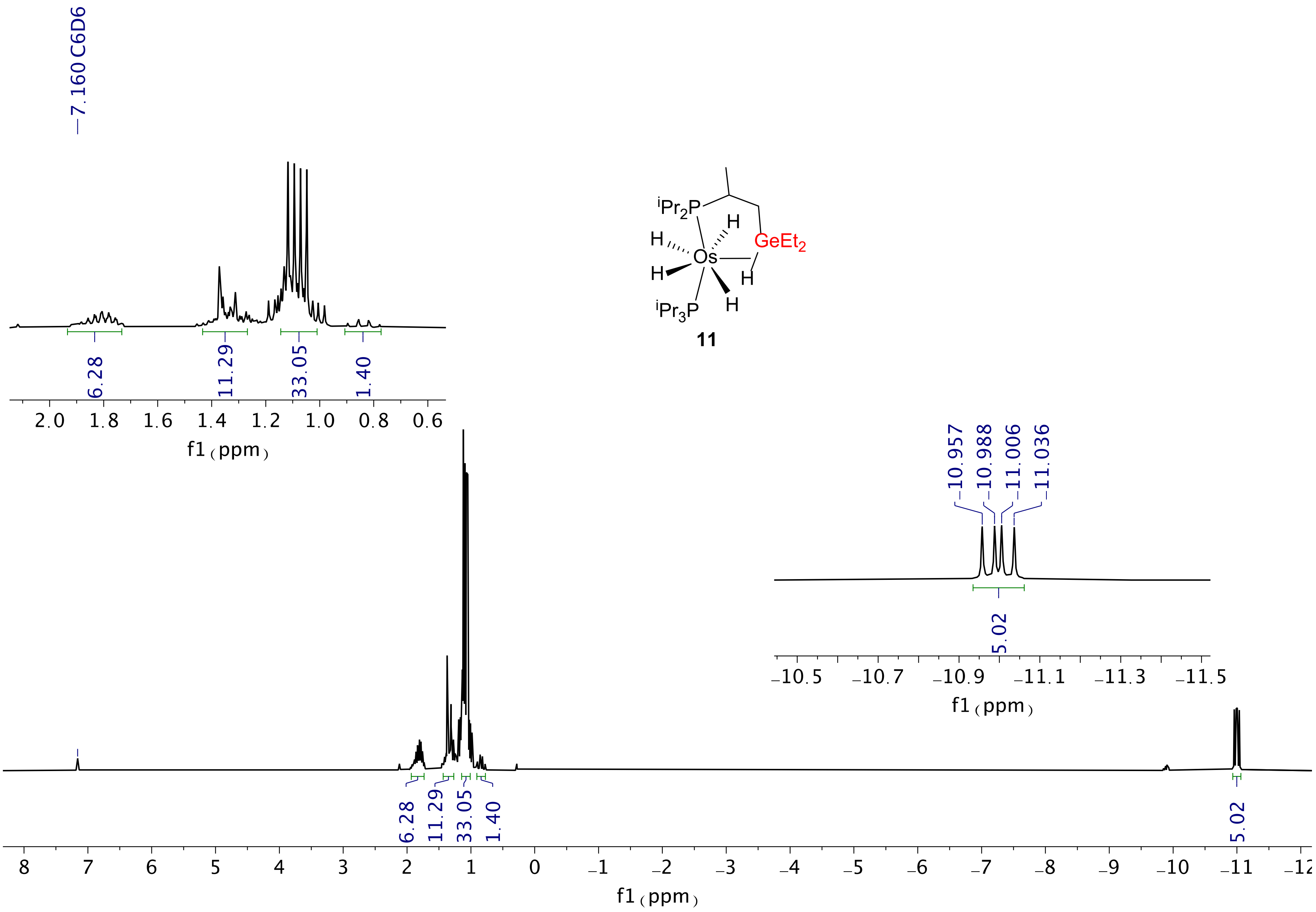

Figure S45. ^{1}H NMR (300.13 MHz, C_6D_6, 298 K) spectrum of **11.**

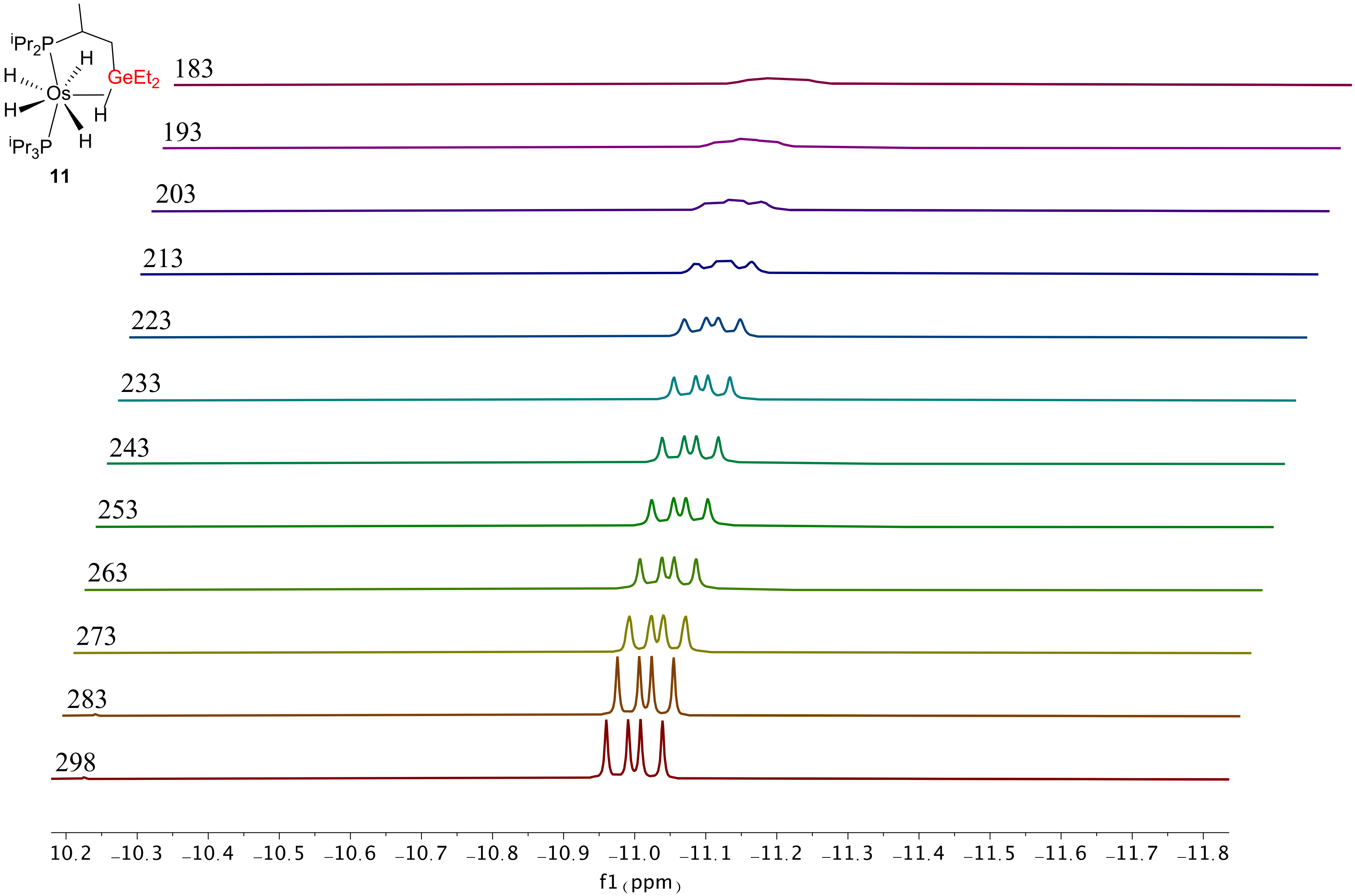

Figure S46. High-field region of the ^{1}H NMR (300.13 MHz, Toluene-d_8) spectrum of complex **11** between 298 and 183 K,

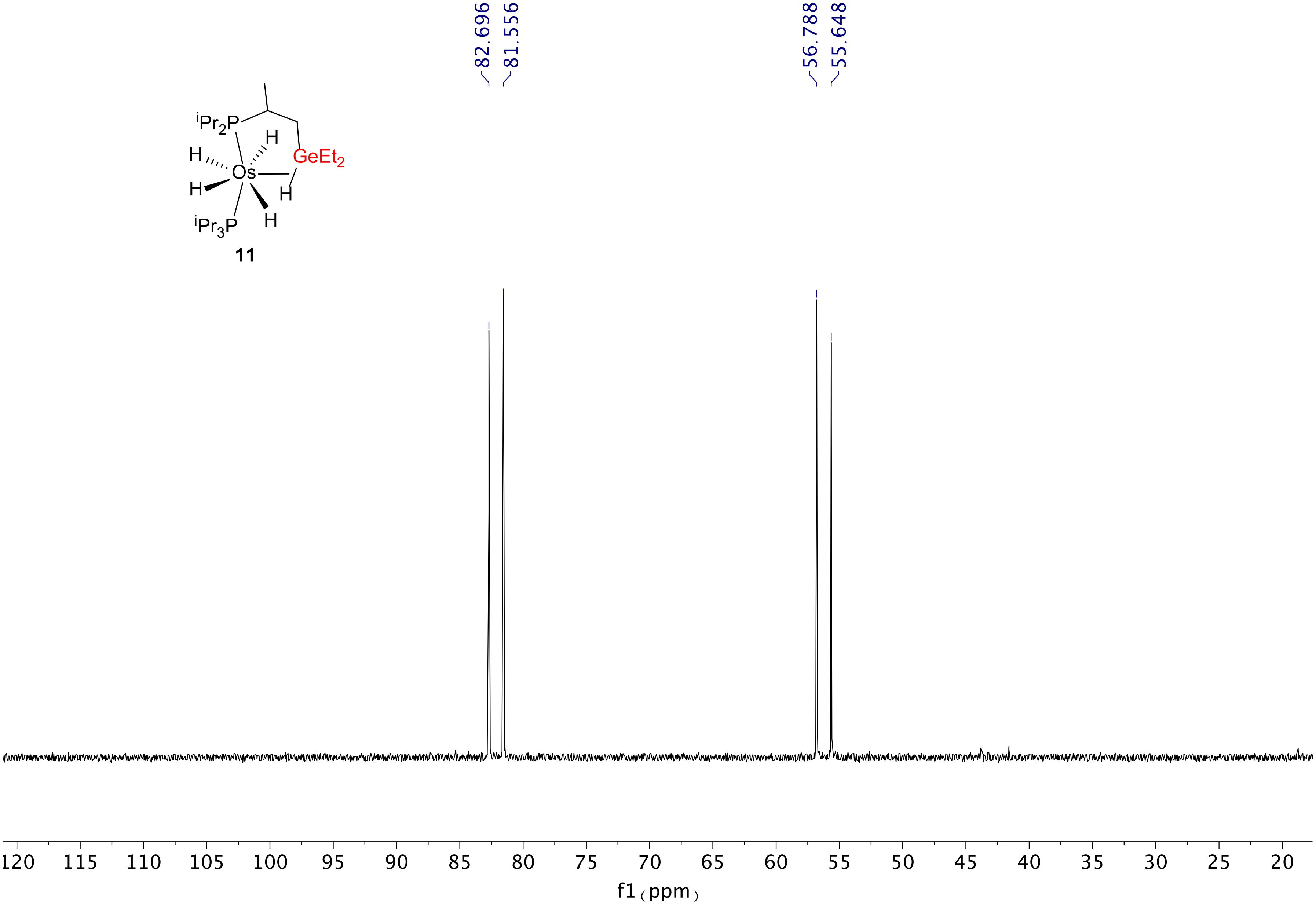

Figure S47. $^{31}P\{^{1}H\}$ NMR (121.50 MHz, C_6D_6, 298 K) spectrum of **11.**

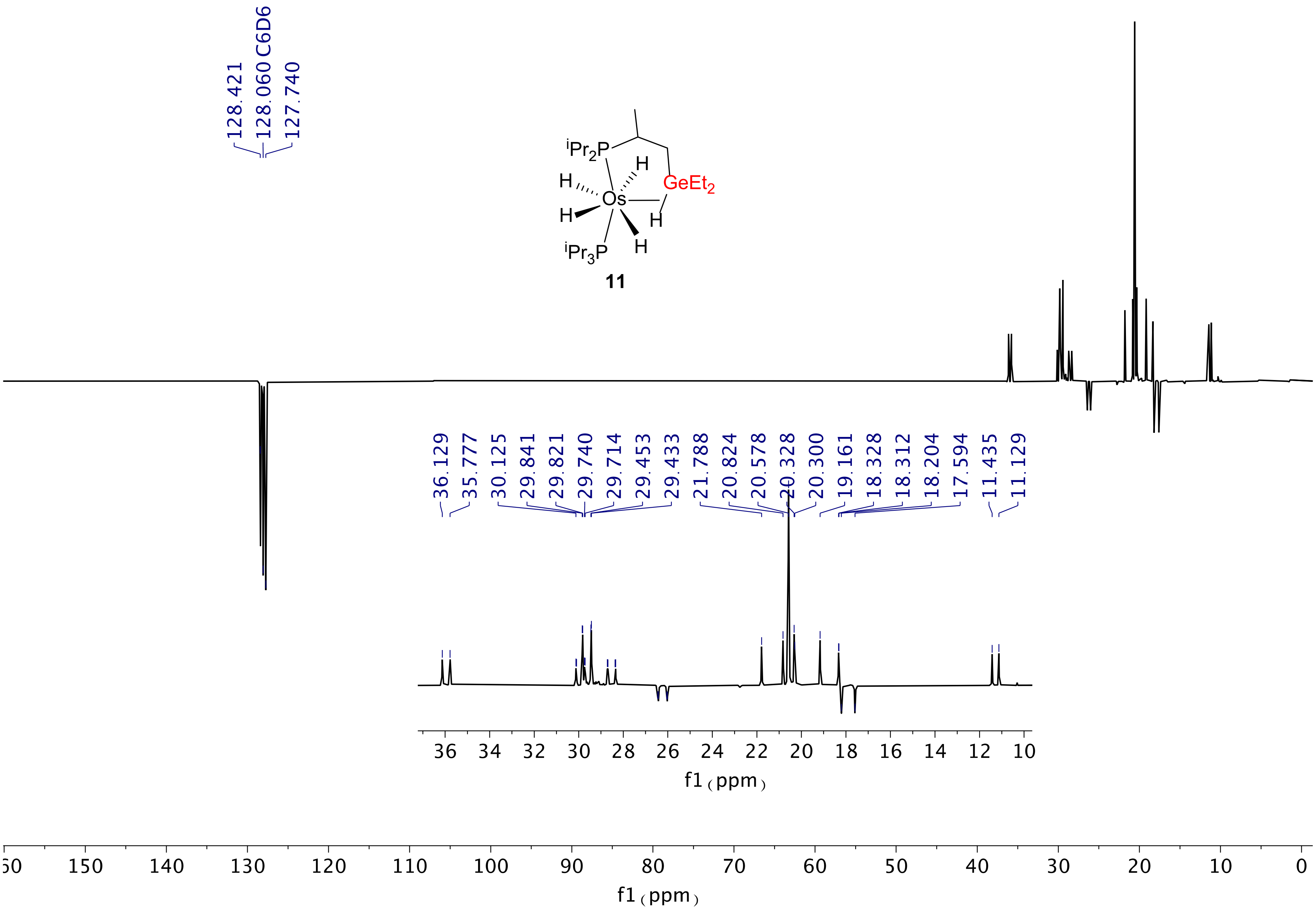

Figure S48. $^{13}C\{^{1}H\}$-apt NMR (75 MHz, C_6D_6, 298 K) spectrum of **11.**
